# Biomass-Based Hydrogen Extraction and Accompanying Hazards—Review

**DOI:** 10.3390/molecules30030565

**Published:** 2025-01-26

**Authors:** Mariusz J. Nieścioruk, Paulina Bandrow, Szymon Szufa, Marek Woźniak, Krzysztof Siczek

**Affiliations:** 1 Mjniescioruk AEI, Traktorowa Str. 55/34, 91-111 Lodz, Poland; mj@niescioruk.eu; 2 Faculty of Civil and Transport Engineering, Poznan University of Technology, Piotrowo Str. 3, 61-138 Poznań, Poland; 3 The Szewalski Institute of Fluid-Flow Machinery Polish Academy of Sciences, Fiszera 14 St., 80-231 Gdańsk, Poland; paulina.bandrow@bader-leather.com; 4 BADER Polska Sp. z o.o., Mostowa 1 St., 59-700 Bolesławiec, Poland; 5 Faculty of Process and Environmental Engineering, Lodz University of Technology, Wolczanska 213, 90-924 Lodz, Poland; 6 Department of Vehicles and Fundamentals of Machine Design, Lodz University of Technology, Stefanowskiego Str. 1/15, 90-537 Lodz, Poland; marek.wozniak.1@p.lodz.pl (M.W.); ks670907@p.lodz.pl (K.S.)

**Keywords:** hydrogen, biomass, hazards

## Abstract

Nowadays, there is an increased demand for energy, the access to which, however, is limited due to the decreasing of fossil sources and the need to reduce emissions, especially carbon dioxide. One possible remedy for this situation is using hydrogen as a source of green energy. Hydrogen is usually bound to other chemical elements and can be separated via energy-intensive few-step conversion processes. A few methods are involved in separating H_2_ from biomass, including biological and thermochemical (TC) ones. Such methods and possible hazards related to them are reviewed in this study.

## 1. Introduction

Substances like coal, oil, and natural gas (NG) and their derivatives are and will be for some time the most extensively utilized energy source worldwide [1]. Their combustion is associated with inherent CO_2_ emissions related to fossil fuels, including coal (38%), NG (25%), and crude oil (23%) [2]. The improvement of such a bad situation requires substituting fossil fuels with green (environmentally friendly) energy sources, including H_2_. 

However, about 48% of H_2_ in the industry comes from synthesis gas, which is produced through the thermal conversion of NG CH_4_ with H_2_O or O_2_ from the air. For CO_2_-rich NG, the main technique is CO_2_ thermal conversion (dry reforming) to generate synthesis gas and H_2_. This technique often utilizes catalysts to lower the energy barrier for the related chemical reactions during this process [3].

Lately, significant focus has been directed toward high-yield biogas with low energy consumption, achievable via the biocatalytic process resulting from the anaerobic transformation of various wastes, including biological ones. Besides H_2_ and various impurities, it includes above 50% CH_4_ and can substitute NG as a H_2_ production source. Unlike direct biocatalytic H_2_ production using bacteria, cyanobacteria, and green microalgae, the hybrid method of thermochemical H_2_ generation from biogas is of high interest today. Direct H_2_ production via the biocatalytic acid generation of organic waste requires the fulfillment of several strict conditions to sustain a specific consortium of microorganisms; alterations in external factors may redirect biochemical reactions toward products that hinder the process, like fatty acids. Their buildup in the working medium changes the pH of the medium toward acidification, thus diminishing H_2_ production. The functioning of methanogenic consortia is influenced by the substrate type, biogas plant material, and operational conditions like temperature, pH, and hydrodynamics. However, they exhibit greater resilience to external factors and environmental components compared to H_2_-producing monocultures. In contrast to pilot bioreactors for H_2_ generation, anaerobic biogas facilities are already integrated into numerous industrial, municipal, and agricultural establishments linked to waste management [3].

Additionally, H_2_ production from other renewable sources became important, as they are C-neutral because the CO_2_ released during their combustion is applied by plants for photosynthesis [4]. Renewable H_2_ (green H_2_) is a pure form of H_2_ obtained from biomass [5]. Of course, Greenhouse Gas (GHG) emissions accompany renewable energy generation due to the small existing effect of the energy incorporated into the materials applied to construct infrastructure for renewable energy [6]. Biohydrogen (bio-H_2_) production is an option to supersede fossil fuels in an environmentally friendly manner [7]. Biomass conversion techniques like gasification (GA) and pyrolysis (PY) have restrictions and consume much energy [4]. Among PY, hydro-pyrolysis (H-PY), reforming, liquefaction, and GA, the last approach is a good thermochemical (TC) method to produce syngas and hydrocarbons from biomass. H_2_ output and C neutralization have been achieved via physicochemical (PC), TC, or biological processes, especially in the case of lignocellulose as a second-generation biomass feedstock [8].

Besides the application of biomass for the production of various types of biofuels, especially biodiesel from various first-generation or edible biomass feedstocks [9], second-generation or non-edible feedstocks [10,11,12,13,14], and third-generation feedstocks [15,16,17], green H_2_ can also be obtained from biomass (e.g., from C_3_H_8_O_3_), with better fuel properties than the other biofuels [18,19].

Recently, various C capture and storage technologies have been integrated with H_2_ production processes to raise the environmental sustainability of H_2_ as a pure energy carrier [20]. However, such integration is accompanied by various risks and hazards.

To explain, a hazard is a potential source of harm or adverse health effect on a person or persons. Risk is the likelihood that a person may be harmed or suffer adverse health effects if exposed to a hazard. The terms hazard and risk are often used interchangeably. The level of risk is often categorized based on the potential harm or adverse health effect that the hazard may cause, the number of times people are exposed to it, and the number of people exposed to it. Control measures (hierarchized) comprise possible actions lowering the potential of exposure to the hazard, removing the hazard, or lowering the likelihood of the risk of exposure to that hazard being realized. The (overall) risk assessment is performed by various methods where the hazard severity and its potential outcomes are considered together with the exposure level, the number of persons exposed, and the risk of that hazard being realized. The residual risk after implementation of control measures should be “as low as is reasonably possible” (ALARP). One must confirm that the expense of lowering the risk further would be exceedingly disproportionate to the advantages obtained [21].

Equipment for extracting H_2_ from biomass is often made of stainless steel [22,23,24,25]. Stainless steel is not hazardous in its solid form. Some processes like cutting, milling, grinding, melting, and welding can cause some unsafe materials to be emitted. In particular, metal fumes may be generated. Cr(VI) carcinogens may result from pickling stainless steel. The mentioned unsafe materials may induce cancer, allergies, or asthma signs or breathing troubles if inhaled. Despite being harmful if swallowed, they probably damage the unborn child and induce damage to organs via prolonged or repeated exposure. Such materials induce eye irritation and may induce allergic skin reactions and respiratory irritation [26]. Employees employed in the extraction of H_2_ from biomass are exposed to hazards occurring when working with equipment specific to process engineering and are to obey basic health and safety requirements [27]. The risks associated with biomass processing are quite comparable to those encountered in the biotechnology sector. Chen and Reniers [28] observed that industrial biotechnology shares several risks with chemical technology but also faces biological dangers associated with biological agents. Workers in the biotechnology sector face health hazards due to various forms of exposure to harmful substances. The outside environment might also be impacted by these agents in instances of unintended release. Various conventional risk assessment techniques can be applied in industrial biotechnology, considering the distinctions between conventional incidents (such as fires, explosions, and toxic emissions) and challenges in industrial biotechnology and chemical technology. Moreover, the evaluation of risks concerning occupational health and safety linked to biological threats can be conducted through exposure analysis and risk characterization. A two-step risk evaluation approach is suggested to evaluate environmental and ecological hazards in industrial biotechnology. Different protective strategies have been examined in [29,30,31].

The goal of this study was to review various hazards and risks related to various methods of H_2_ extraction from biomass.

## 2. Methods

The literature from electronic databases such as “ISI Web of Science”, “Scopus”, and “Google Scholar” was retrieved via “logical” database searches using various keywords, such as: “hydrogen”, ”biohydrogen”, ”biomass”, “hazards”, and ”risk”. The sources for the extraction of H_2_ from biomass and associated risks covered the years 1998–2024. As English-language publications are commonly accessible to readers worldwide, mainly those published in that language are featured. Similarly to [32], this review explores the techniques employed to transform biomass into hydrogen through electrochemical, biological, and thermochemical processes. The advantages, disadvantages, progress, and risks associated with each method are examined. Moreover, a techno-economic analysis was performed, and an evaluation of the environmental impacts of these methods was carried out. The expenses associated with H_2_ processes, the challenges of techno-economic commercialization, and the financial evaluation of various H_2_ production methods were also considered.

## 3. Various Hazards Related to Hydrogen, Biohydrogen, and Biomass

H_2_ is a pure option for CH_4_, also known as NG. It contributes about 75% of the mass of the universe. It can be obtained from NG, nuclear power, biogas (BG), and renewable power like solar and wind. The challenge is harnessing H_2_ as a gas on a large scale [33].

H_2_ production from fossil fuels demands greater energy and liberates CO_2_, mainly enhancing the greenhouse effect. Such production from a renewable source is a sustainable and environmentally friendly process. Bio-H_2_ is a clean and C-free fuel. It is widely applicable in transportation to the generation of electricity. Bio-H_2_ is obtained by fermentation. The commonly used bacterial species are *Clostridium* sp. and facultative anaerobes of the Enterobacteriaceae family. Agro-residues are good sources for producing bio-H_2_. Primarily, pretreated lignocellulosic biomasses are utilized to produce bio-H_2_. Bio-H_2_ production is also possible from non-pretreated lignocellulosic biomass [23].

Biohydrogen as a fuel exhibits some advantages, including its C-neutral or C-zero nature, ready renewability, environmentally effective generation via biological routes, eco-friendly transformation, and maximum energy content compared to other fuels [24].

Bio-H_2_ is a clean, non-toxic energy carrier [25].

Being a clean, C-zero option to non-renewable fossil fuels, bio-H_2_ possesses a great energy yield of 122 kJ/g [34].

“Biomass” relates to the unfossilized and biodegradable organic substances from plants, animals, and microorganisms. Biomass comprises byproducts, residues, and waste from farming, forestry, and associated industries. It also comprises unfossilized and biodegradable organic fractions of factory and municipal wastes, gases, and liquids obtained from decomposing unfossilized and biodegradable organic matter [27].

It is a renewable organic complex originating from plants and animals. It can be burned directly for heat or transformed into liquid and gaseous fuels via diverse processes [33].

Biomass sources for energy include the following [33]:

- Wood and its processing waste—firewood, wood pellets, and chips; lumber and furniture mill sawdust and waste; and black liquor from pulp and paper mills.
- Farming crops and waste materials—corn, soybeans, sugar cane, switchgrass, woody plants, algae, crop, and food processing residues, applicable to biofuels’ production.
- Biogenic substances in municipal solid waste-paper products; cotton and wool products; and food, yard, and wood waste.
- Animal manure and human sewage for BG production.

Biomass transformation to energy is realized via diverse processes, like the following [33]:

- Direct combustion (burning) to produce heat.
- TC transformation for producing solid, gaseous, and liquid fuels.
- Chemical transformation for producing liquid fuels.
- Biological transformation for producing liquid and gaseous fuels.

### 3.1. Various Hazards Related to Hydrogen and Biohydrogen

H_2_ energy features comprise a lack of local pollution during combustion and H_2_O as the sole byproduct. Compared with CH_4_ and gasoline, H_2_ has several advantages [35,36]:

▪ During accidental outdoor operation, H_2_ disperses faster than the other fuels.
▪ H_2_ has the greatest flammability range in the air (4–77% by volume) with no safety issues.
▪ The H_2_ flame speed (346 cm/s) exceeds that of CH_4_ 8-fold (43.25 cm/s).
▪ H_2_ burns with low docility, so its fast consumption is accompanied by little damage to the adjacent elements.
▪ The H_2_ explosion range exceeded those of the other two fuels but H_2_ deflagrate at higher volume concentrations.
▪ The H_2_ energy per unit mass (LHV = 120 MJ/kg) thrice exceeds that from gasoline combustion (40 MJ/kg).

The primary risks associated with H_2_ storage are the following:

▪ Ability to ignite easily.
▪ Dangers posed by high-pressure and low-temperature storage conditions.
▪ Potential to penetrate small gaps or porous materials due to its small molecular size [37].

H_2_ can be explosive at concentrations of 18.3–59% in contact with air [38].

As a highly inflammable and explosive substance, H_2_ cannot be easily transported from one place to another compared to CH_4_ and gasoline [39].

A large spill of liquid H_2_ is like one of gasoline; however, it dissipates much quicker. Another virtual danger is an explosion of a boiling liquid expanding vapor when the pressure relief valve fails. H_2_ onboard a car may induce a safety hazard [40].

H_2_ is non-toxic and lighter compared to air; it dissipates suddenly when released, allowing for the relatively sudden dispersal of the fuel when a leak occurs. Therefore, additional engineering controls are required to provide for its safe use [41].

High H_2_ concentrations can induce an O_2_-scarce milieu. People inhaling such an atmosphere may experience signs like headaches, ringing in the ears, dizziness, drowsiness, unconsciousness, nausea, vomiting, and the depression of each sense [42].

While H_2_ production generates no GHG emissions, H_2_ combustion creates harmful pollutants like NO_x_. These are associated with smog, acid rain, and damaging health influences such as asthma and respiratory infections [43].

If not produced using renewable sources, H_2_ pollutes. More than 96% of the H_2_ used is gray, highly affecting the environment, as 10 kilos of CO_2_ is produced for every kilo of H_2_ obtained [44].

During bio-H_2_ production, the H_2_ yield is low due to byproduct formation and the complex nature of biomass [45].

The use of green H_2_ is accompanied by the virtual risk of H_2_O scarcity. The generation of green H_2_ needs a high H_2_O amount, and in some areas where H_2_O is already scarce, such an enhanced demand could exacerbate present H_2_O shortages [46].

During H_2_ generation from biomass, usually, various gases can be released, including mainly CO, CO_2_, N_2_, NO_x_, CH_4_, and C_2_H_6_ [39].

CH_4_ is an extremely combustible and explosive gas that ignites rapidly when subjected to heat, sparks, or flames. When CH_4_ builds up to 5–15% by volume in an air mixture, it creates an extremely explosive gas. Above 15% (150,000 ppm), there is a lack of sufficient O_2_ in the air (danger of asphyxiation) [47].

CH_4_ additionally functions as an asphyxiant. The level of exposure and symptoms can vary from mild to severe and from acute to chronic, depending on the concentration and duration of exposure. CH_4_ gas can be deadly when present at high concentrations over extended periods [48].

The mitigation of hazards comprises the following measures [48]:

- When handling CH_4_, all risks of ignition and explosion must be removed from the surrounding area, and smoking is prohibited nearby.
- When CH_4_ might exist in an enclosed area, its levels ought to be regulated and assessed alongside O_2_ levels.
- CH_4_ contained in a cylinder needs to be safeguarded against harm and maintained in an upright stance. When stored in facilities, CH_4_ must be placed in a cool, well-aired area away from direct sunlight and other potential ignition sources.
- If there is a suspicion of a CH_4_ leak, it is essential to clear the area right away.
- If a person is exposed to CH_4_, wearable units can notify them to seek fresh air to avoid methane gas poisoning.

C_2_H_6_ is an extremely flammable gas, which may form explosive mixtures with the air. C_2_H_6_ gas stored in a tank under pressure may explode if heated. It may also displace O_2_ and cause rapid suffocation. Exposure to swiftly expanding gas can lead to burns or frostbite [49].

The mitigation of hazards comprises the following measures [49]:

- Attempting to heat frozen tissues and obtaining medical assistance.
- Staying clear of heat, hot surfaces, sparks, open flames, and other sources of ignition. No use of tobacco products.
- Do not attempt to extinguish a fire caused by leaking gas unless the leak can be safely closed. Remove all ignition sources if it is safe to do so.
- Shielding tanks containing C_2_H_6_ from sunlight and keeping them in a properly ventilated area.

### 3.2. Various Hazards Related to Biomass Storage

The storage of biomass carries several significant concerns as well as health and safety problems, including self-ignition and mold inhalation. Both bacterial or fungal diseases and biomass fires will probably occur, especially in large biomass facilities. The primary health concern is linked to hazardous molds found in wood chips. Normally, Aspergillus Fumigatus leads to severe health issues such as Aspergillosis, various respiratory problems, pulmonary and cardiac infections, asthmatic issues, etc. Aspergillosis is seen as uncommon. Another dangerous situation arises when biomass produces CO in storage rooms because of inadequate air circulation [50]. 

Biomass creates significant fire and explosion hazards. In addition to the risks to personnel, there may be significant environmental risks related to fire fighting and the pollution effects arising from fire H_2_O run-off. The size of the potential fire area can be great, so it can become an issue [51].

A great deal of energy is wasted because of large amounts of biomass waste. Not only does the waste pollute the environment by infiltrating soil, H_2_O, and air, but certain pollutants, such as heavy metals, are also absorbed by and build up in living organisms, leading to significant harm to their systems [52]. The primary issue linked to landfills is the generation of leachate and BG. Leachate is the waste H_2_O produced as a result of biochemical activities in waste substances [53]. It has a significant quantity of organic materials and contaminants, which taint land, ground H_2_O, and H_2_O bodies. Landfill BG primarily consists of CH_4_ (approximately 40–70% by dry volume), which has 20–25 times more potent warming effects compared to CO_2_ [54]. In addition to landfilling, burning waste results in CO_2_, ash, H_2_O, and various other byproducts. In contrast to PY, incineration occurs in an O_2_-rich environment [55].

Accelerated industrialization, the ongoing application of heavy metals in manufacturing techniques, and inadequate industrial waste management have led to higher metal concentrations in the environment [56]. The buildup of heavy metals in soil impacts fertility and leads to the creation of polluted land, which in turn alters the quality of food [57]. The buildup of heavy metals in H_2_O and air leads to significant harm to the species that are exposed to them. Potential health concerns are identified, including mental disorders, developmental irregularities, neuromuscular issues, changed metabolic functions, infertility, organ dysfunction, and cancer development in humans [58], whereas plants experience cellular harm, ionic balance disruption, O_2_ pressure, and the inhibition of crucial enzymes, microelements, and pigments, as well as interrupted photosynthesis and respiration [59].

Prolonged exposure to environments with elevated levels of heavy metals threatens the variety and lifespan of all living organisms [60].

Strategies for the mitigation of hazards related to biomass storage include processing biomass into hydrogen and other materials using technologies such as pyrolysis (PY). Additionally, biochar, obtained through PY and co-PY, can be used for soil remediation to stabilize heavy metals, thus reducing the phytotoxicity and bioavailability of heavy metals [61]. Biochar promotes the transformation of heavy metals into residual fractions, thus decreasing the accumulation of heavy metals in plants [62]. Moreover, the higher pH value of biochar also favors the immobilization of heavy metals [63].

## 4. Methods for Renewable-H_2_ Production from Biomass

Among various renewable energy sources, H_2_ is deemed the purest energy generator since its only byproduct is H_2_O, resulting in genuinely zero pollutant emissions. Consequently, creating effective H_2_ production technologies that make use of biomass resources and guaranteed clean energy with minimal C emissions is essential in combating global warming and achieving waste recovery [64].

H_2_ is usually bound to other chemical elements and can be separated via energy-intensive few-step conversion processes. For example, Gladchenko et al. [3] optimized the thermophilic anaerobic bioconversion of wheat distillery vinasse, either pure or linked with cow and chicken manure, focusing on the amount of CH_4_ and BG as a potential source for H_2_ production.

**Figure 1:**
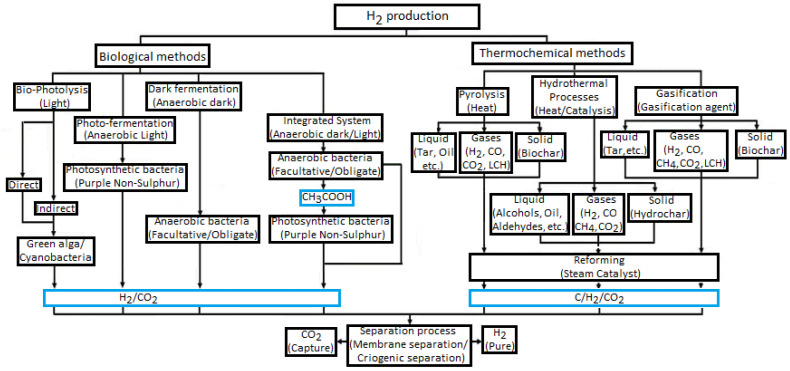
Methods for separating H_2_ from biomass (based on [39]).

Additionally, there are two methods for separating H_2_ from biomass: biological and TC ones. Renewable H_2_ relates to bio-H_2_ from microorganisms and H_2_ produced via the TC conversion of biomass (Figure 1) [39]. Recently, electrochemical processes have also started to be applied for the conversion of biomass into hydrogen [65]. The final product of such methods needs to be subjected to a separation and purification process to provide the high-quality H_2_ needed by an efficient power system [66].

### 4.1. Biological Methods Used for Hydrogen Production from Biomass and Hazards Related to It

According to [67], the production of H_2_ through the conversion of biological biomass may play a key role in future biorefinery systems. Biological methods for producing hydrogen are considered limitless and advantageous for the environment. It allows for producing renewable and C-neutral H_2_. Numerous biological methods for H_2_ production have been developed and applied, such as fermentation, bio-photolysis, enzymatic reactions, and microbial electrolysis. Fermentation for the generation of H_2_ or H_2_-enriched gases can be categorized into two types according to the requirement for light in the process, specifically dark fermentation (light-independent) and photo-fermentation (light-dependent). In the photo-fermentation process, photosynthetic bacteria use solar energy to generate H_2_ from organic materials. In addition, bio-photolysis can be categorized into direct and indirect types.

#### 4.1.1. Bio-Photolysis

H_2_ production via bio-photolysis relies on photosynthetic O_2_-producing microorganisms like green microalgae (such as *Chlamydomonas reinhardtii*, *Chlorella vulgaris*, *Scenedesmus obliquus*, and *Chlorococcum minutum*) and cyanobacteria (such as *Nostoc* sp. and *Cyanothece* sp.) [68,69,70,71,72,73]. 

These microorganisms use sunlight to power photosynthesis, which splits H_2_O and creates H_2_ with the help of the enzyme hydrogenase (H_2_ase) as a catalyst [74].

Bio-photolysis helps direct the reductants from splitting H_2_O for H_2_ production without utilizing the Calvin cycle or pentose phosphate pathway, entailing photochemical oxidation in the thylakoid membrane of algae and cyanobacteria. In these organisms, two functional assemblies are formed by light-absorbing pigments: photosystems (PSs) I and II. In milieus without O_2_ or with high energy intake, specific microorganisms direct extra electrons toward hydrogenase, transforming proton (H^+^) ions into sustainable H_2_. The electrons and protons combine in this process to produce 98% pure renewable H_2_ via the chloroplast enzyme dehydrogenase. Bio-photolysis falls under the direct bio-photolysis (DbP) or indirect bio-photolysis (i-DbP) category [75,76].

**Figure 2:**
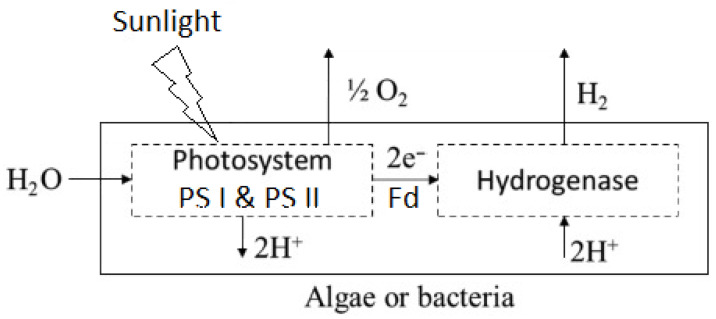
The flowchart of DbP (based on [76]).

In the absence of O_2_, photoautotrophic organisms in DbP utilize the enzyme hydrogenase to transform H_2_O molecules into molecular H_2_ and O_2_ with the help of light absorbed by PSII. These organisms use light energy to remove electrons and protons from H_2_O molecules. The captured electrons and protons enable the reduction of ferredoxin (Fd) and nicotinamide adenine dinucleotide phosphate (NADP) [77]. Figure 2 shows the flowchart of DbP (based on [76]).

Formulas (1) and (2) [49] outline the general reactions of DbP.

(1) $$H_{2}O\overset{Light}{\to }2H^{+}+2e^{-}+0.5O_{2},$$

(2) $$2H^{+}+2e^{-}\overset{Hydrogenase}{↔}2H_{2}$$

Even though DbP has a high yield, its production is hindered by the O_2_ released by photosystem II (PSII), which significantly restricts H_2_ases and weakens H_2_ production [39].

In the i-DbP process, cyanobacteria and microalgae produce H_2_ and O_2_ separately in two steps. During the initial phase, carbohydrates are created with the help of light energy, CO_2_ is trapped, and O_2_ is produced. H_2_ is produced from the synthesized carbohydrates via cell metabolism in the anaerobic milieu in the second step [78,79].

**Figure 3:**
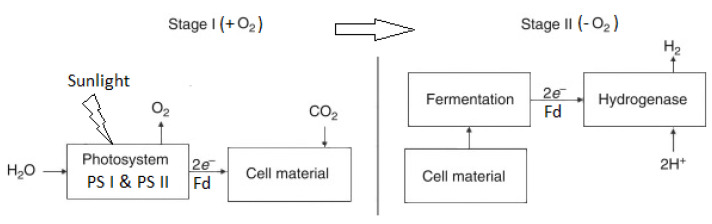
The flowchart of i-DbP.

Cyanobacteria are predisposed to be involved in i-DbP because they employ nitrogenase (N_2_ase) and H_2_ase enzymes for H_2_ production, unlike microalgae, which only use H_2_ase. N_2_ase operates in one direction, whereas H_2_ase works in both directions. The process of H_2_ formation by cyanobacteria is explained via Formulas (3) and (4) [80].

(3) $$6CO_{2}+6H_{2}O\overset{Light}{\to }C_{6}H_{12}O_{6}+6O_{2},$$

(4) $$C_{6}H_{12}O_{6}+6H_{2}O\overset{Light}{\to }12H_{2}+6O_{2}$$

i-DbP starts with cyanobacteria fixing CO_2_ and uses sunlight for the production of cellular components and O_2_ [81]. NADPH produced by metabolism moves to the plastoquinone pool (PQ) and PSII. Ferredoxin transports the electrons produced by PSII via both PSII and PSI to H_2_ase. The latter facilitates the reaction in which H^+^ is converted into sustainable H2 [78]. Figure 3 shows the flowchart of i-DbP (based on [79]).

i-DbP has disadvantages, such as poor effectiveness, restricted catalyst supply, sensitivity to the milieu, and technological hurdles [73,77,82].

N_2_ fixation is carried out by the H_2_ase uptake, consisting of small (hupS) and large (hupL) subunits. N_2_ase can reabsorb the H_2_ released by H_2_ase while also removing O_2_ from the system. This process helps to safeguard enzymes sensitive to O_2_ and results in an indirect boost in H_2_ production. Certain filamentous cyanobacteria, such as Anabaena, Calothrix, and Nostoc, have produced heterocysts that support N_2_ fixation and can increase H_2_ production.

The second phase of i-DbP shares some characteristics with anaerobic fermentation processes. The benefit of i-DbP, in contrast to DbP, is that the O_2_ generation phase is distinct from the H_2_ evolution phase, preventing O_2_ from inhibiting H_2_ evolution [83,84].

Rey et al. [39] stated that H_2_ production in bio-photolysis is primarily influenced by temperature, pH, O_2_ levels, brightness, N_2_ and S constraints, the availability of organic C sources, cell concentration, and growth stage.

S is a combustible material and poses a fire and explosion hazard at temperatures above 232 °C. Harmful gases generated in fire include H_2_S, SO_2_, and SO_3_ [85].

S has a low level of toxicity to humans. Nevertheless, consuming excessive S might lead to a burning feeling or diarrhea. Inhaling S dust may irritate the respiratory passages or lead to coughing. It may also cause irritation to the skin and eyes [86].

The dangers of H_2_S arise during the storage or transport of molten sulfur. H_2_S may build up in enclosed areas [87].

The mitigation of hazards comprises the following measures [87]:

- Keeping solid S in a ventilated space, away from substances that may react with it.
- Implementing suitable engineering measures or respiratory safeguards.
- It is advised to wear safety goggles when exposed to high levels of dust.
- Wearing a face shield for safety from molten S.
- Steering clear of continuous or extended skin contact.
- To safeguard against molten S, it is advised to use gloves and skin protection made from leather or heat-resistant materials.

C is highly flammable in the powder form. The latter is combustible. Finely dispersed particles form explosive mixtures in the air [88].

The mitigation of hazards comprises the following measures [88]:

- Avoiding open flames, sparks, and smoking.
- Storing in a closed system, dusting explosion-proof electrical equipment and lighting, and preventing the deposition of dust.
- Preventing the buildup of electrostatic charges (e.g., by grounding).
- Wearing safety goggles.

Information on the hazards related to bio-photolysis is shown in Table 1.

The effects of the main parameters on H_2_ yield in bio-photolysis are shown in Table 2.

Cyanobacteria and microalgae have received significant attention among the microorganisms that can generate H_2_ [89].

Essential research focused on enhancing the efficiency of H_2_ production through photosynthetic cultures and accelerating the reaction rate of this process is necessary to address issues related to the concurrent emission of O_2_ and H_2_ while ensuring the process remains highly sensitive to O_2_. Within this framework, studies focused on increasing the H_2_ production rate by photosynthetic bacteria are currently very significant.

In principle, transformation technology such as chemical catalysis, biocatalysts, or a combination of both can produce 12 mol of H_2_ for every mole of glucose derived from biomass sugars. Nonetheless, the majority of experimental studies employing those methods did not achieve optimal H_2_ production due to the formation of undesired products and severe reaction conditions [90]. Scientists also explored an enzymatic method to enhance H_2_ efficiency from biomass. Zhang et al. [91] showed the excellent performance of the enzymatic process utilizing starch and H_2_O, with H_2_ production being approximately 70% of the theoretical prediction. Additionally, 13 enzymes aided the study, which occurred at a temperature of 30 °C.

H_2_ can be generated by transforming substrates with specific enzymes that do not yield undesired byproducts. The required H_2_ gas can then be conveniently obtained from the reactor, enabling sustained production. Since the enzymatic process does not involve cellular membranes that can impede mass transfer, unlike the microbial process, it can operate more rapidly to enhance H_2_ production [92]. This method yields more than dark fermentation (DF—described further), boasts a faster production rate, and can expedite non-natural processes. Several studies on enzymatic hydrogen production have been carried out by scientists [92].

During bio-photolysis, Song et al. [93] obtained a H_2_ yield range of 140–160 (mL/L) utilizing *Chlorella* sp. as a microorganism and 30 mM glucose as a substrate. The process was realized in the temperature range of 25–42 °C, with a light intensity (LI) of 120 μmol/m^2^/s, an incubation time (IT) of 70 h, and a pH of 8.6, using MA as a medium and a serum bottle reactor (Table 1).

In a similar process, Sengmee et al. [94] produced a H_2_ yield of 11.65 (mL/L) using *C. vulgaris* as a microorganism and crude C_3_H_8_O_3_ as a substrate. The process was realized at a temperature of 30 °C, with an LI of 48 μmol/m^2^/s, an IT of 72 h, and a pH of 6.8, using modified Tris-Acetate-Phosphate (TAP) as a medium and a 1 L bioreactor (Table 1).

Then, Pyokim et al. [95] obtained a H_2_ yield of 225 (mL/L) utilizing *C. reinhardtii* as a microorganism. The process was realized at a temperature of 25 °C, with an LI of 200 μmol/m^2^/s, an IT of 140 h, and a pH of 7.2, using TAP as a medium and 250 mL Erlenmeyer flasks as reactors (Table 1).

Also, during bio-photolysis, Bala Amutha and Murugesan [96] produced a H_2_ yield of 220 (mL/L) using *C. vulgaris* as a microorganism and corn stalk as a substrate. The process was realized at a temperature of 30 °C, with an LI of 108 μmol/m^2^/s, an IT of 144 h, and a pH of 7.0, using modified Blue-Green (BG-11) as a medium and a 500 mL bioreactor (Table 1).

Then, Chader et al. [97] obtained a H_2_ yield of 147 (mL/L) utilizing *C. sorokiniana* as a microorganism and acetate as a substrate. The process was realized at a temperature of 30 °C, with an LI of 120 μmol/m^2^/s, an IT of 222 h, and a pH of 7.2, using BG-11 as a medium and 500 mL Erlenmeyer flasks as reactors (Table 1).

With the same reactor, Hong et al. [98] obtained a H_2_ yield of 118 (mL/L) using *C. reinhardtii* as a microorganism and starch as a substrate. The process was realized at a temperature of 28 °C, with an LI of 50 μmol/m^2^/s, an IT of 144 h, and a pH of 7.5, using TAP-C as a medium (Table 1).

In a similar process, Huesemann et al. [99] produced a H_2_ yield of 115 (mL/L) utilizing *P. boryanum* as a microorganism and 3-(3,4-dichlorophenyl)-1,1-dimethylurea (DCMU) as a substrate. The process was realized at a temperature of 22 °C, with an LI of 50 μmol/m^2^/s, an IT of 188 h, and a pH of 7.5, using 0.5 mM N as a medium and Roux bottles as reactors (Table 1).

During their study, Vargas et al. [100] obtained a H_2_ yield of 61.7 (mL/L) using *C. reinhardtii* as a microorganism. The process was realized at a temperature of 24 °C, with an LI of 60 μmol/m^2^/s, an IT of 204 h, and a pH of 7.2, using TAP-S as a medium and 500 mL Duran glass bottles as reactors (Table 1).

Then, Vargas et al. [101] produced a H_2_ yield of 13.15 (mmol H_2_/mg Chla) utilizing *Anabaena* sp. as a microorganism and glucose as a substrate. The process was realized at a temperature of 24 °C, with an LI of 4400 lux, an IT of 156 h, and a pH of 9.2, using BG-11 as a medium and 500 mL Duran glass bottles as reactors (Table 1).

Also, using the cyanobacterium *Anabaena* sp., Vargas et al. [102] generated H_2_ through Ni_2_ limitation in two phases of growth. They found that improving biomass during the initial culture phase increased production by 18.3%, and heterocyst formation was 3.4 times more significant in N_2_-deficient conditions.

During their study, Raksajit et al. [103] obtained a H_2_ yield of 3.61 (mmol H_2_/mg Chla) using *Arthrospira* sp. as a microorganism and 0.1% glucose as a substrate. The process was realized at a temperature of 30 °C, with an LI of 40 μmol/m^2^/s, an IT of 156 h, and a pH of 9.0, using Zarrouk medium [104] without nitrate (ZN_0_) as a medium and 500 mL Duran glass bottles as reactors (Table 1).

With the same reactor, Vargas et al. [105] obtained a H_2_ yield of 9.23 (mmol H_2_/mg Chla) utilizing *Chlamydomonas* sp. as a microorganism. The process was realized at a temperature of 24 °C, with an LI of 60 μmol/m^2^/s, an IT of 372 h, and a pH of 7.2, using TAP-S as a medium (Table 1).

Kossalbayev et al. [89] investigated the production of biological H_2_ from various strains of cyanobacteria, such as *Synechocystis* sp. PCC 6803, *Desertifilum* sp. IPPAS B-1220, *Synechococcus* sp. I12, and *Phormidium corium* B-26. *Synechocystis* sp. produced H_2_ accumulation. PCC 6803 reached a peak of 0.037 μmol/mg Chl/h after 120 h in darkness. The native, filamentous, non-heterocystous cyanobacterium *Desertifilum* sp. IPPAS B-1220 achieved a peak of 0.229 μmol/mg Chl/h in the gas phase after 166 h of light exposure, matching the highest yield documented in existing research. DCMU at a concentration of 10 μM enhanced H_2_ production by *Desertifilum* sp. IPPAS B-1220, which increased by 1.5 times to 0.348 μmol H_2_/mg Chl/h. *Desertifilum* sp. IPPAS B-1220 produced H_2_ in the light at a 20-fold greater rate than in the dark during studies aimed at discovering new cyanobacterial strains that can generate and enhance conditions for H_2_ production. The cyanobacteria could effectively transform solar energy into molecular H_2_.

A significant challenge in bio-H_2_ production is the presence of O_2_ generated during the process, which poses a major barrier to obtaining H_2_ from biomass and organic solid waste [106]. In the presence of O_2_, the activity of enzymes, transcription processes, and protein maturation may be suppressed [107]. Scientists are working to enhance H_2_ production from biomass and organic solid waste while tackling the issue of O_2_ molecule presence. Melis [108] showed that H_2_ase enzyme activity in algae cultures, like *Chlamydomonas*, requires the absence of O_2_. To enhance H_2_ production from green algae, Paramesh and Chandrasekhar [109] employed O_2_ scavengers like Na_2_SO_3_, Na_2_S_2_O_5_, and Na_2_S_2_O_4_. They found that all three scavengers could enhance H_2_ production. Due to its elevated O_2_ usage, Na_2_SO_3_ yielded the best results. NaHSO_3_ has been utilized before to enhance H_2_ production in *Chlamydomonas reinhardtii* [110]. The findings indicated that a minor amount of sodium bisulfite in the examined algae could effectively extract O_2_. Surzycki et al. [111] studied the application of O_2_ blockers with Cu to enhance H_2_ production in algae farming. 

Efficient bioreactors and genetic engineering are essential elements in creating a sustainable biological method for H_2_ production. The latter is considered pioneering research regarding the enhancement of H_2_ production during the process [106]. Certain photosynthetic bacteria, including cyanobacteria and *Rhodobacter sphaeroides*, have undergone genetic engineering [112,113]. Since most studies are conducted on a laboratory scale, the reactor’s efficient design needs to be developed on a larger scale. When designing bioreactors, crucial factors to consider include temperature regulation, the stirring mechanism, bioreactor efficiency with identical volumes but different area–volume ratios, and the capacity to manage consortium organisms [114].

**Table 1:** Hazards related to bio-photolysis used for H_2_ production during diverse studies.

Microorganism/Substrate/Operating Conditions	Information of Hazards	Refs.
*Chlorella* sp.; 30 mM glucose; T: 25–42 °C; LI: 120 μmol/m^2^/s; IT: 70 h; pH: 8.6; Medium: MA; Reactor type: serum bottle reactor.	MA-Medium for fresh-H_2_O, terrestrial, hot-spring, and salt-H_2_O algae. Some types of algae are harmful when they grow too quickly or make toxins. Toxins from algal cells or those released into H_2_O can make people and animals ill when they encounter these toxins via food or H_2_O. At times, algal blooms can grow so thick that they block sunlight, preventing other aquatic plants and animals from obtaining the amount needed for their survival. Thick blooms can similarly obstruct the gills of fish, shellfish, and other creatures, hindering their ability to breathe. When a bloom fades away, the decomposition process can consume all the O_2_ in the H_2_O, leading to the suffocation of other aquatic life. When a bloom deteriorates, it might emit gases like CH_4_ and H_2_S that can pose dangers to humans. The mitigation of hazards comprises the following measures: Restrict nutrient pollution or fertilizers in H_2_O to prevent the growth of harmful algal blooms. Decrease the quantity of nutrients that enter H_2_O via the use of the proper quantity of fertilizer and maintain the septic system.	[115,116,117]
	*Chlorella* sp.—The cells of Chlorella sp. do not create any harmful substances and are regarded as safe for people to eat (when used as food supplements based on such microalgae). There are serious worries about the quality of products made from such microalgae because they may be contaminated with toxic metals, inorganic arsenic, or cyanotoxins. The mitigation of hazards comprises the following measures: Use only premium Chlorella products and make sure that the food is well formulated to prevent any possible negative impacts on fish health and productivity.	[118]
	*Chlorella* sp. has no negative impact on fish health, growth, or immune system function, but more research is necessary to understand its long-term effects on fish.	[119]
	Glucose is a safe substance or mixture under Regulation (EC) No 1272/2008 [120].	[121]
	Serum bottles are commonly made of transparent polyester (PET) material (inedible). This substance is harmless, has no flavor, and is highly transparent.	[122]
	Serum bottles made from Wheaton 400 borosilicate molded glass can also meet USP Type I requirements.	[123]
	Cyanobacteria, also known as blue-green algae, induce most toxic algal blooms in fresh H_2_O. Diatoms or dinoflagellates (red tides) are responsible for inducing the most harmful algal blooms in salt H_2_O. Prevention: Similar to the case of MA.	[116]
	Excessive algal growth deprives or restricts other types of marine life and obstructs the sunlight needed for their rapid proliferation. Issues with taste and smell in drinking H_2_O and fish deaths are linked to high levels of planktonic algae blooms. Prevention: Similar to the case of MA	[124]
*C. vulgaris*; Crude glycerol; Temp.: 30 °C; LI: 48 μmol/m^2^/s, IT: 72 h; pH: 6.8; Medium: Modified TAP; Reactor type: 1 L bioreactor	Administering *Chlorella vulgaris* orally to mice in both acute and multiple doses did not result in any toxicity or adverse reactions.	[125]
	No physical or behavioral changes were observed with different doses of *C. vulgaris*, and there were no signs of pain or distress, suggesting that C. vulgaris is not toxic. A study based on OECD Guideline 420 found no acute liver damage in female SD rats when given *Chlorella vulgaris* at 2000 mg kg^−1^ BW. Therefore, C. vulgaris falls into the unclassified category within the classification of GHS.	[126]
	Crude C_3_H_8_O_3_/Glycerin can induce skin irritation (H315) and organ damage (H370) and is dangerous if ingested (H302). Excessive mist buildup can cause irritation of the respiratory tract. It can cause temporary eye discomfort (burning, stinging, and tearing). Possible health impacts relate to the following: Eyes: Contact can lead to slight eye discomfort. Skin: Exposure might lead to skin irritation. Ingestion: It is of minimal toxicity. It could be dangerous if swallowed. It can result in nausea, headaches, and diarrhea. Inhalation: Due to its low vapor pressure, it is improbable that vapor would be inhaled at room temperature. Breathing in mist can lead to irritation of the respiratory system. The mitigation of hazards comprises the following measures: P260: Avoid inhaling dust/fume/gas/mist/vapors/aerosol. P264: Clean well after contact. P270: Avoid eating, drinking, or smoking while using this product. P280: Utilize protective gloves/protective clothing/eye safety/face protection. P301 + P312: If ingested, contact a poison control center or medical professional if feeling unwell. P302 + P352: In case of skin contact, rinse thoroughly with soap and H_2_O. P309 + P311: If exposed or feeling unwell, contact a poison center or a doctor/physician. P330: Wash out the mouth. P332 + P313: In case of skin irritation, seek medical advice/assistance. P362: Remove soiled clothing and launder it before wearing it again. P405: Store secured. P501: Dispose of the contents/container according to local, regional, national, and international regulations.	[127,128]
*C. reinhardtii*; -; T: 25 °C; LI: 200 μmol/m^2^/s; IT: 140 h; pH: 7.2; Medium: TAP; Reactor type: 250 mL Erlenmeyer flasks	Dried biomass powder of *C. reinhardtii* (THN 6) did not show mutagenic properties in the bacterial reverse mutation test at the highest recommended concentration for soluble non-cytotoxic substances and did not exhibit clastogenic effects in the chromosomal aberrations test at the maximum cytotoxic concentration. The micronucleus test showed that THN 6 dried biomass powder did not exhibit genotoxic effects in vivo when tested at the maximum dose. In the end, the administration of 6 dried biomass powders via gavage to male and female HSD showed no specific organ effects or toxicity. Han Wistar rats were fed 4000 mg/kg bw/day doses.	[129]
	Erlenmeyer flasks made of PTFE: According to CLP regulation (EC) No. 1272/2008 [120], this product is not classified as an unsafe substance/unsafe mixture. Products can induce burns in hot conditions. Heating PTFE above 400 °C can induce unsafe vapors. The latter can induce irritation in the eyes, nose, throat, and lungs. The mitigation of hazards comprises the following measures: If the product becomes too hot, ensure there is sufficient ventilation or exhaust when the product is heated. Utilize respiratory protective equipment if the ventilation is inadequate. An evaluation of occupational exposure is needed to identify appropriate eye/face protection. If hot PTFE contacts your eyes, rinse them with cold water for a minimum of 15 min. Do not attempt to take out such hot material. Seek medical advice/attention without delay. Steer clear of touching the skin. In the case of contact with hot PTFE, rinse the skin right away with cold water for a minimum of 15 min. Avoid attempting to take out the hot material. Dress the wounded area with sterile bandages. Seek medical advice or attention right away. Wearing chemically resistant protective gloves is not essential. When working with hot PTFE, use thermal-insulating gloves to prevent burns. When PTFE products are utilized in compliance with regulations, there is no need for respiratory protection. If inhaled, promptly relocate to an area with fresh air. Seek medical guidance if you experience discomfort.	[130]
	TAP-TAP (Tris-Acetate Phosphate) Medium induces skin irritation (H315). It also induces serious eye irritation (H319). In a blend including, i.a., CuSO_4_.5H_2_O and ZnSO_4_·7H_2_O, it might be harmful to the user or the environment or may be presumed to be so. The mitigation of hazards comprises the following measures: P280: Use protective apparel/protective gloves/eye safety gear. P305 + P351 + P338: In case of eye contact, gently rinse with water for a few minutes. Take out contact lenses if they are in place and simple to remove. Keep rinsing.	[131]
*C. vulgaris*; Corn stalk; T: 30 °C; LI: 108 μmol/m^2^/s; IT: 144 h; pH: 7.0; Medium: Modified BG-11; Reactor type: 500 mL bioreactor	*C. vulgaris*—as mentioned.	
	Corn stalk-The cornstalk plant is moderately toxic to pets. In dogs, it induces signs of gastrointestinal upset with vomiting and diarrhea. Mitigation of hazards: Avoid contacting pets with the corn stalk. If necessary, contact a veterinarian.	[132]
	Corn plant sap is toxic, especially to inquisitive kids and pets. Consuming the corn plant may result in nausea, vomiting, and diarrhea. The intensity of these symptoms frequently depends on the quantity ingested. Continuous contact with the toxins in the corn plant can lead to long-term health problems. The mitigation of hazards comprises the following measures: It is vital to recognize the dangers and refrain from excessive consumption of corn plants or its products. If ingestion happens, it is crucial to obtain medical help quickly to reduce possible long-term consequences.	[133]
	Cornstalk plants are toxic to dogs, cats, and horses. It can cause vomiting (seldom with blood), depression, anorexia, hypersalivation, and dilated pupils in cats.	[134]
	Modified BG-11 (Blue-Green) Medium can harm fertility or the developing baby (H360). It causes irritation of the skin (H315) and eyes (severely) (H319). It can cause irritation of the respiratory system (H335). The mitigation of hazards comprises the following measures: P221: Take all necessary measures to prevent contact with flammable materials. P280: Use protective clothing/gloves/eye protection. P305 + P351 + P338: If it contacts the eyes, rinse gently with water for a few minutes. Take out contact lenses if in place and simple to remove. Keep rinsing. P308+P313: If exposed or worried, seek medical advice/assistance. P405: Store secured. P501: Dispose of contents/container in compliance with federal, state, and local environmental laws.	[135]
*C. sorokiniana*; Acetate; T: 30 °C; LI: 120 μmol/m^2^/s; IT: 222 h; pH: 7.2; Medium: BG-11; Reactor type: 500 mL Erlenmeyer flasks	*Chlorella sorokiniana* can contain heavy metals (Hg, Pb, Cd) if its culture medium is not strictly controlled, such as culture in a non-glass tube. The composition of samples of material containing *Chlorella sorokiniana* should be systematically monitored.	[136]
	Some people may experience gastrointestinal signs such as nausea, diarrhea, gas, or abdominal pain. The mitigation of hazards comprises the following measures: People who are allergic to I_2_ or have thyroid problems and troubles need to be extremely careful or avoid chlorella containing naturally present I_2_. *Chlorella* should also be avoided by people with autoimmune diseases and by pregnant and breastfeeding women.	[137]
	Ethyl acetate is an extremely flammable liquid and vapor (H225). It causes severe irritation of the eye (H319). It can cause drowsiness or dizziness (H336). Repeated exposure can cause skin dryness or cracking (EUH066). The mitigation of hazards comprises the following measures: P210: Avoid exposure to heat, hot surfaces, sparks, open flames, and other sources of ignition. No tobacco use. P233: Ensure the container is securely closed. P240: Ground and connect the container and receiving apparatus. P241: Utilize explosion-safe electrical/ventilation/lighting equipment. P242: Utilize tools that do not create sparks. P305 + P351 + P338: If in contact with eyes, gently rinse with water for a few minutes. Take out contact lenses if in place and simple to manage. Keep rinsing.	[138]
	BG11 Broth-An oxidizer able to amplify fire (H272). It causes severe irritation of the eye (H319). The mitigation of hazards comprises the following measures: P210: Avoid exposure to heat, hot surfaces, sparks, open flames, and other sources of ignition. No tobacco use. P220: Stay clear of apparel and other flammable substances. P264: Cleanse skin thoroughly after contact. P280: Use protective gloves/protective clothing/eye protection/facial protection. P305 + P351 + P338: In case of eye contact, rinse gently with water for a few minutes. Take out contact lenses if they are in place and easy to remove. Keep rinsing. P337 + P313: If eye irritation continues, seek medical advice/attention.	[139]
	Erlenmeyer flasks-as mentioned.	
*C. reinhardtii*; Starch; T: 28 °C; LI: 50 μmol/m^2^/s; IT: 144 h; pH: 7.5; Medium: TAP-C; Reactor type: 500 mL Erlenmeyer flasks	*C. reinhardtii*—as mentioned.	
	Starch is not an unsafe substance or mixture. It may form an explosible dust–air mixture if dispersed. The mitigation of hazards comprises the following measures: When the product is managed correctly, dangerous effects are not probable. Respiratory protection is not necessary, but it is important to avoid inhaling the dust since even harmless dust can adversely affect respiratory function. Use appropriate body armor. To safeguard the skin, use gloves when handling the material. Wear appropriate safety goggles that have side protection. Avoid allowing the product to go into drains.	[140]
	Starch has the potential to create flammable dust levels in the air (while being processed). The mitigation of hazards comprises the following measures: Prevent material from reaching children. Avoid eating, drinking, or smoking while using this product. Ensure adequate exhaust ventilation. Under normal usage conditions, respiratory protection is not necessary. Utilize appropriate respiratory protection when high levels are present or when aerosol or mist is created. In cases of spills, it might be wise to use respiratory protection. To safeguard the skin, the glove material must be waterproof and durable against the product, substance, or formulation being used or handled. Eye safety requires the use of safety glasses with side shields or goggles. Standard safety precautions must be followed when working with chemicals. Maintain distance from food, drinks, and feed supplies. Promptly take off all dirty and contaminated garments. Clean hands before taking breaks and after finishing work. Avoid breathing in gases, fumes, dust, mist, vapor, or aerosols. Steer clear of contact with the eyes and skin.	[141]
	TAP—as mentioned. However, TAP-C—no information.	
	Erlenmeyer flasks—as mentioned.	
*P. boryanum*; DCMU; T: 22 °C; LI: 50 μmol/m^2^/s; IT: 188 h; pH: 7.5; Medium: 0.5 mM N; Reactor type: Roux bottle	Administration of lyophilized microalgal biomass suspension of *P. boryanum* at doses of 300 and 2000 mg.kg^−1^ showed no toxicity signs, indicating its safety according to its OECD classification as “Minimal Toxicity or Secure”.	[142]
	DCMU is toxic when ingested (H302). It is believed to cause cancer (H351). Prolonged or repeated inhalation of the substance may induce harm to organs (blood) (H373). It has high toxicity to aquatic organisms with long-lasting effects (H410). The mitigation of hazards comprises the following measures: P202: Do not touch until all safety measures have been read and comprehended. P260: Avoid inhaling dust. Respiratory protection is necessary when dust is produced. P264: Cleanse skin thoroughly following handling. Utilize appropriate gear for skin safety. P273: Prevent discharge into the environment. Avoid letting the product go down the drain. P301 + P312: If ingested, contact a poison center/doctor if you feel unwell. P308 + P313: If exposed or worried, seek medical advice/attention. Wear protective clothing and suitable gear for eye safety.	[143]
	Roux bottle—Roux laboratory bottles made of Borosil are chemically resistant and stable.	[144]
*C. reinhardtii*; -; T: 24 °C; LI: 60 μmol/m^2^/s; IT: 204 h; pH: 7.2, Medium: TAP-S; Reactor type: 500 mL Duran glass bottles.	*C. reinhardtii*—as mentioned.	
	TAP—as mentioned. However, TAP-S—no information.	
	Duran glass bottles are made from 3.3 borosilicate glass, exhibiting exceptional thermal shock resistance and chemical compatibility, ensuring safe handling of diverse substances.	[145]
*Anabaena* sp.; Glucose; T: 24 °C; LI: 4400 lux; IT: 156 h; pH: 9.2; Medium: BG-11, Reactor type: 500 mL Duran glass bottles.	*Anabaena* sp. is non-toxic and not pathogenic (hazard class: 1).	[146]
	*Anabaena* spp. can generate anatoxin-a, anatoxin-a(s), saxitoxin, and microcystins. Anatoxin-a affects nerve synapses. It works as a postsynaptic cholinergic nicotinic agonist, inducing a depolarizing neuromuscular blockade.	[147]
	Glucose—as mentioned.	
	BG-11—as mentioned.	
	Duran glass bottles—as mentioned.	
*Arthrospira* sp.; 0.10% glucose; T: 30 °C; LI: 40 μmol/m^2^/s; IT: 156 h; pH: 9.0; Medium: ZnO.	*Arthrospira* sp. is safe to ingest by animals and humans.	[148]
	ZN_0_—a complex medium, no information about hazards.	[148]
	Glucose—as mentioned.	
*Chlamydomonas* sp.; -; T: 24 °C; LI: 60 μmol/m^2^/s; IT: 372 h; pH: 7.2; Medium: TAP-S; Reactor type: 500 mL Duran glass bottles.	*Chlamydomonas* sp. is non-toxic.	[149]
	TAP—as mentioned. However, TAP-S—no information.	
	Duran glass bottles—as mentioned.	

Abbreviations: Tris-Acetate-Phosphate (TAP), Blue-Green (BG), 3-(3,4-dichlorophenyl)-1,1-dimethylurea (DCMU).

The operational conditions should be optimized to enhance H_2_ generation via bio-photolysis using microalgae or cyanobacteria [39].

Microorganisms used during bio-photolysis usually exhibit no or low toxicity or pathogenicity. Only for *Anabaena* spp., information was found indicating that they can produce several kinds of toxins. Materials used as reactors exhibited no toxicity. Materials used as substrates or media can exhibit different levels of toxicity depending on the type and application. For ZN_0_, being a complex medium, no information was found about hazards. However, it is expensive, and various cheaper substitutes for ingredients [104] are used instead of the original ones. The resulting toxicity level of the resulting mixtures remains unknown and requires further research.

**Table 2:** Effects of the main parameters on H_2_ yield in bio-photolysis.

Parameters	Effects on H_2_ Yield in Bio-Photolysis	Refs.
Temperature	The ideal temperature for H_2_ production significantly differs between species.	[39]
	The peak H_2_ yields for cyanobacteria are found at temperatures between 30 and 40 °C; however, certain strains show maximum production within a cooler temperature range of 20–25 °C.	[150,151]
pH	Cyanobacteria demonstrated the greatest H_2_ production at a pH of 8.0, while an acidic pH of 4.5 led to an 83% decrease in H_2_ output. The ideal pH range for achieving peak H_2_ production in most species lies between 6.0 and 8.0. H_2_ production significantly declined at a starting acidic pH (5.0) and gradually increased under alkaline conditions, attaining peak yield at pH 9.0.	[150]
O_2_ content	O_2_ in the milieu hinders renewable-H_2_ production by the enzyme hydrogenase, and production stops entirely when O_2_ levels completely deactivate the catalytic activity. Producing O_2_ and H_2_ simultaneously necessitates the extraction of O_2_ from the milieu to maintain process efficacy.	[89]
Light intensity	H_2_ production by *C. reinhardtii* demonstrated a gradual increase in yield from 60 to 200 µE m^−2^ s^−1^, ultimately achieving peak production. Raising the light intensity to 300 µE m^−2^ s^−1^ showed light saturation accompanied by a reduction in H_2_ production.	[95]
N_2_ and S limitation	It was reported that ongoing H_2_ production from cyanobacteria achieves the greatest yield when deprived of N_2_ and S.	[103]
	The S absence creates an anaerobic setting, boosting the capacity of microalgae to generate H_2_.	[152]
Organic carbon	Organic carbon sources enhance the growth of mixotrophic cyanobacteria and aid in H_2_ production by fostering an anaerobic milieu suitable for achieving greater process efficacy.	[101]
Cell density and culture age	Elevated cell density within the photobioreactor limits light access for each cell, diminishing photosynthesis and elevating respiration. Even younger cultures with lower biomass demonstrate greater H_2_ production because the exponentially growing cells are more metabolically active than the older cells.	[101]

Loyte et al. [153] noticed that methods like enhancing growth conditions, genetic modification, employing microalgal consortia, creating advanced photobioreactor designs, and utilizing enzymatic processes are utilized to gain a clearer understanding and consequently devise strategies and techniques to boost H_2_ production from microalgae. Obstacles like inadequate production rates, significant expenses associated with various stages of cultivating the desired traits of microalgae, and understanding the evolutionary trends under production conditions, environmental influences, and numerous difficulties in harvesting have presented serious challenges, highlighting the urgent need for progress in genetic engineering, photo-biochemistry, and photobioreactors. Economic feasibility is yet another challenge that can be addressed through enhancements in comprehension of the existing generative model. Methods that are still uncertain with today’s technology but show significant potential for future progress in the present model of inference and synthesis encompass artificial photosynthesis, synthetic biology, improved materials for photobioreactors, enhanced microalgal consortia, the application of nanomaterials, and sophisticated process control systems. 

Bio-photolysis is in the developing stage, and its cost is 2.13 USD/kg. The process drawbacks include the high O_2_ sensitivity of hydrogenase and low light conversion efficacy. The remedy for them is the requirement for proper enzymes. The process efficiency is in the range of 10–11% [154].

No matrices of risks related to any solutions for hydrogen extraction from biomass using bio-photolysis were found in the literature. Thus, more studies are needed in this area.

#### 4.1.2. Photo-Fermentation

H_2_ is generated in photo-fermentation (PF) during the breakdown of organic compounds by photosynthetic bacteria like purple non-sulfur bacteria (PNS), such as *Rhodo-bacteria sphaeroides*, *Rhodobacter capsulatus*, *Rhodobacter sulfidophilus*, *Rhodopseudo-monas palustris*, and *Rhodospirillum rubrum*. Light energy assists N_2_ase in catalyzing this process [155,156]. The tricarboxylic acid (TCA) cycle metabolizes organic substrates to produce the necessary reducing power and C intermediates for H_2_ generation [157]. H_2_ production by PNS bacteria via the TCA cycle involves multiple biochemical processes. 

Firstly, the C source is oxidized to generate CO_2_, H^+^ ions, and electrons. N_2_ase is supported by the oxidation/reduction of electron carriers such as NAD(P)H and ferredoxin (Fd) during the transfer of the latter. At that moment, PSI in the photosynthetic membrane uses light energy to produce the ATP required for N_2_ase activity in collaboration with ATP synthase. Furthermore, N_2_ase converts protons (H^+^) into molecular H_2_. Additionally, H_2_ase enzymes are involved in metabolism, transforming H_2_ into electrons and protons in specific circumstances [158]. According to Formula (5), CH_4_ production results in the creation of one H_2_ molecule for every N_2_ molecule fixed [159].

(5) $$N_{2}+8e^{-}+8H^{+}+16ATP\to 2NH_{3}+H_{2}+16ADP+16P_{i},$$

In the absence of N_2_, N_2_ase does not convert N_2_ but instead facilitates a different reaction producing H_2_, as shown in Equation (6).

(6) $$8e^{-}+8H^{+}+16ATP\to 4H_{2}+16ADP+16P_{i}$$

Under these circumstances, bacteria in the peripheral nervous system (PNS) use organic acids like acetic acid (C_2_H_4_O_2_), butyric acid (C_4_H_8_O_2_), or lactic acid (C_3_H_6_O_3_) to generate H_2_ [160]. They can also use monosaccharides, such as glucose, and polysaccharides, such as starch, to produce H_2_ [161]. Nevertheless, this response requires a high consumption of energy within the cell, specifically in the form of ATP molecules. Despite being energy-intensive, this process is very efficient for producing H_2_ because all protons can be converted to renewable H_2_ [159]. 

The PF process yields 4 moles of H_2_ gas per mole of glucose, with CH_3_COOH being the result. Yet, the yield decreases to 2 mol H_2_/mol glucose if butyric acid is the result, as outlined in Equations (7) and (8) [161,162].

(7) $$C_{6}H_{12}O_{6}+2H_{2}O\to 2C_{2}H_{4}O_{2}+4H_{2}+2CO_{2},$$

(8) $$C_{6}H_{12}O_{6}\to C_{4}H_{8}O_{2}+2H_{2}+2CO_{2}$$

In the absence of O_2_, the thermodynamics of acid production promotes the formation of CH_3_COOH and C_4_H_8_O_2_, leading to their presence in the metabolites of the ultimate PF output. Thus, the H_2_ production is consistently less than 4 mol H_2_/mol glucose [163].

**Figure 4:**
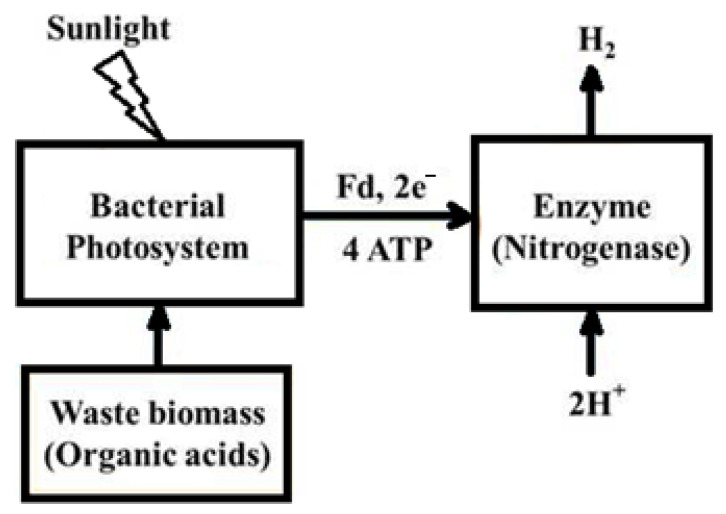
The flowchart of PF (based on [156]).

Figure 4 shows the flowchart of PF (based on [156]).

Similar to anaerobic conversion, the PF process involves the fermentative transformation of organic substrates into bio-H_2_ by various photosynthetic bacteria through a sequence of three biochemical steps [67]. 

Photosynthetic bacteria such as *Rhodospirillum*, *Rhodobacter*, *Rhodobium*, and *Rhodopseudomonas* can produce H_2_ via their nitrogenases. Due to its significant advantages of abundant material resources and comprehensive substrate utilization, PF H_2_ production has garnered interest in recent years [79].

The rate of H_2_ production rises with higher light intensity, while the efficiency of light conversion decreases. When compared to cyanobacteria, utilized in indirect bio-photolysis, the photosynthetic bacteria in the PF pathway demonstrate a higher efficiency in light conversion [67].

Basak et al. [164] studied purple non-S bacteria cultivated in an O_2_-poor milieu employing N_2_ase and an organic acid as a reducing agent for H_2_ production. This method has multiple shortcomings, outlined as follows:

- Minimal efficiency in converting solar energy (3–10%).
- The requirement for extensive anaerobic photo-bioreactors.
- The reliance on nitrogenase enzymes, which require significant energy for activation.

The activity of N_2_ase is essential for H_2_ production in photosynthetic bacteria. Hydrogenase functions in both H_2_ production and H_2_ uptake under identical conditions. The primary benefit of this approach is the elevated concentration of H_2_ in the resulting gas stream [67]. Tao et al. [165] indicated a concentration of approximately 96% H_2_ in the product gas stream.

Another benefit of this technique is that the organic acid used as a substrate is present in the wastewater discharge of various industries. The effluent from DF can be utilized as a substrate for PF [166,167]. This method could be utilized to generate H_2_ from various types of biomass waste [168]. 

Utilizing only C sources, H_2_ production was demonstrated to rise by 2–3 times [169]. 

Fedorov et al. [170] introduced the bacterial system and the efficiency of H_2_ conversion for different feedstocks.

The effectiveness of PF in producing H_2_ depends on anaerobic milieus, light intensity, temperature, pH levels, light wavelength, the concentration of the substrate, and the type of substrate [67]. 

N_2_ase is very responsive to O_2_ and is permanently deactivated by it [159]. Therefore, increasing the production of H_2_ is achieved by creating a low-O_2_ milieu in the reaction mixture [158]. For effective H_2_ production, the ideal conditions are temperatures between 31 and 36 °C, LI between 6 and 10 klux, pH levels between 6.8 and 7.5, and a wavelength between 400 and 1000 nm. Another important factor is the amount and composition of the substrate [171]. Bacteria from the PNS group produce more H_2_ when utilizing fatty acids such as short-chain and volatile fatty acids (acetate, butyrate, lactate, malate, etc.) compared to when using sugar substrates [77,109].

Numerous research efforts focused on photosynthetic bacteria have been recorded in the literature; however, the PF process has never been found to be an economically viable method. PF techniques provide various benefits, such as utilizing a significant amount of feedstock and harnessing abundant heat from sunlight. The problems that still need to be addressed include the lower volumetric flow rate of H_2_, the efficiency of conversion, and the requirement for a large surface area [67].

Details regarding hazards associated with PF are displayed in Table 2.

In the process of PF, Cheng et al. [172] achieved a total H_2_ yield of 463 (mL H_2_/g vs) by utilizing *C. butyricum* and *R. palustris* as starter cultures and rice straw as the substrate at 30 °C, pH 7.0, and LI conditions of 6000 lux illuminance (Table 2).

In a similar manner, García-Sánchez et al. [173] achieved a total H_2_ yield of 260 (mL H_2_/L) by employing *R. pseudopalustris* DSM 123 as an inoculum and tequila vinasses as a substrate at a temperature of 30 °C, a pH of 7.0, and LI from an LED lamp (13.5 W/m^2^). The inoculum was in the form of a cell suspension with a concentration of 3.3 g/L (Table 2).

In a study by Yue et al. [174], a total H_2_ yield of 74.0 (mL H_2_/g TS) was achieved with an inoculum containing HAU-M1 (*R. sphaeroides* (9%), *R. palustris* (28%), *R. rubrum* (27%), *R. capsulata* (25%), and *R. capsulatus* (11%)), along with corn stover as the substrate. The inoculum contained 30% TS with 150 mg/g, and 100 mg/L of TiO_2_/activated carbon fiber was added. The procedure was carried out at a pH of 7.0 and an LI of 3000 lux (Table 2).

Lu et al. [175] achieved a total H_2_ yield of 68.4 (mL H_2_/g DM) under the conditions of 30 °C temperature, pH 7.0, and an LI of 3000 lux using an identical inoculum and substrate with a concentration of 25 gDM/L (Table 2).

Likewise, Budiman and colleagues [176] achieved a total H_2_ yield of 14.4 (mL H_2_/mL medium) by using *R. sphaeroides* NCIMB8253 as the inoculum and a substrate consisting of a mixture of palm oil (25%, *v*/*v*) and pulp and paper (75%, *v*/*v*) mill effluents. The mixed substrate consisted of 25% POME and 75% PPME. The procedure was carried out at a temperature of 30 degrees Celsius and a light intensity of 7000 lux (Table 2).

PF is in the developing stage and is characterized by a cost of 3.7 USD/kg. PF has drawbacks such as low H_2_ yield and an efficiency equal to 11.9% (after using a catalyst). The remedy for them is utilizing appropriate catalysts [177,178].

No matrices of risks related to any solutions of H_2_ extraction from biomass using bio-photolysis were found in the literature. Thus, more studies are needed in this area.

#### 4.1.3. Dark Fermentation

**Figure 5:**
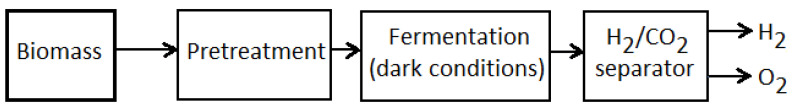
The flowchart of DF (based on [180]).

DF primarily uses anaerobic microorganisms such as *Enterobacter cloacae*, *Escherichia coli*, *Klebsiella pneumoniae*, and *Bacillus subtilis* (facultative anaerobes) and *Clostridium butyricum*, *Clostridium acetobutylicum*, *Clostridium thermocellum*, and *Thermoanaerobacterium thermosaccharolyticum* (strict anaerobes) in the absence of light at temperatures ranging from 25 to 45 °C for mesophilic conditions, 45 to 80 °C for thermophilic conditions, and above 80 °C for extreme thermophilic conditions. Additionally, gases like CO_2_, CO, and H_2_S may also be produced under these conditions. Glucose molecules in carbohydrates and other feedstocks are the main source of H_2_ [179,180]. Figure 5 shows the flowchart of PF (based on [180]).

The metabolic pathways utilized by microorganisms are the primary factors influencing the H_2_ yield and effluent composition in dark fermentation (DF). Facultative anaerobes utilize the first pathway, which involves pyruvate formate lyase (PFL). The second one, which is used by strict anaerobes, includes pyruvate ferredoxin oxidoreductase (PFOR) [181]. In the process of generating H_2_ from glucose-rich substances, intricate compounds are initially broken down into simpler molecules, such as pure glucose. Afterward, it is broken down without O_2_ to create NADH, pyruvate, and ATP [163]. During the PFL pathway, pyruvate converts to formate and acetyl-CoA with the help of coenzyme A (CoA-H) present. Formate is oxidized to CO_2_ and H_2_ via either the formate-H_2_ lyase (NiFe-H_2_ase) pathway or a formate-dependent [FeFe] H_2_ase pathway. In the PFOR route, pyruvate is converted into reduced ferredoxin and acetyl-CoA through the actions of ferredoxin oxidase and CoA-H. Ferrodoxin that has been reduced is subsequently oxidized to produce hydrogen gas using a ferredoxin-dependent enzyme called H_2_ase (Fd-[FeFe]) [182].

Additionally, H_2_ can be produced by NADH via the reduction of ferredoxin, the reduction of a H_2_ase (NADH-[FeFe]), or the oxidation of NADH by Fd-NADH-[FeFe]. Acetyl-CoA has the potential to be converted into CH_3_COOH, C_4_H_8_O_2_, or CH_3_CH_2_OH with the help of NADH, resulting in a variety of PF liquid byproducts. Additional types of liquid waste, including propionate, butanol, and lactate, may also be produced [163].

Hydrolysis, acidogenic fermentation, acetogenesis, and methanogenesis make up the anaerobic digestion (AD) process [183]. Both fermentation and anaerobic digestion (AD) are processes that occur without O_2_. 

Ellofy et al. [67] explained that the DF process consists of the initial two stages of AD (hydrolysis and acidogenesis) to generate H_2_, while in anaerobic AD, the goal is to create BG that can be subsequently refined into bio-CH_4_. The primary distinctions between them lie in their operational parameters. To generate H_2_ in an anaerobic setting, it is necessary to inhibit methanogens (H_2_ users). Extracellular hydrolytic enzymes assist in breaking down complex organic molecules that are not directly consumable by bacteria into soluble monomers like monosaccharides, amino acids, and other basic organic components in the initial phase. Obligate anaerobes like Bacteroides, Clostridia, and various facultative bacteria perform this stage. In the hydrolysis process, the degradation rate is primarily influenced by the type of substrate. The breakdown of hemicellulose and cellulose takes place at a slower rate than protein decomposition [184]. Acidogenic fermentation bacteria convert the products of hydrolysis into short-chain organic acids like butyric acid (C_3_H_7_COOH), propanoic acid (CH_3_CH_2_COOH), and acetic acid (CH_3_COOH), along with alcohols, H_2_, and CO_2_ during the second stage. During this phase, simple sugars, fatty acids, and amino acids are typically converted into acetate, CO_2_, and H_2_ (70%), along with volatile fatty acids (VFAs) and alcohols (30%) [67].

DF’s effectiveness in transforming substrates into H_2_ is restricted by the presence of other microorganisms that consume H_2_ in the mixed microbial community. This includes homo-acetogens, methanogens that use H_2_, bacteria that reduce sulfate, bacteria that reduce nitrate, and producers of propionate, all of which decrease the overall production of H_2_.

There is no evidence in the literature of any risks associated with homo-acetogens impacting humans or the environment.

Methanogenic Archaea contributes to the development of periodontal disease in specific patients by acting as a hydrogen sink, allowing pathogenic secondary fermenters to grow more extensively than they would without the presence of the archaea.

Methanogens, archaea that thrive in O_2_-free milieus, convert bacterial fermentation byproducts into CH_4_ through the reduction of CO_2_, acetate fermentation, or the breakdown of CH_3_OH or methylamines. Euryarchaeota, such as *Methanobrevibacter smithii*, *Methanobrevibacter oralis*, *Methanobrevibacter arbophilus*, *Methanobrevibacter massiliensis*, *Methanomassiliicoccus luminyensis*, *Methanosphaera stadtmanae*, and Ca, are the only methanogens found in the human microbiota. *Methanomethylophilus alvus* and Ca. are both mentioned. M. intestinalis is a type of *Methano-massiliicoccus* found in the intestines. Methanogens are recently discovered harmful organisms associated with abscesses in the brain and muscles. They were involved in the imbalance of the oral microbiota, as well as periodontitis and peri-implantitis [185]. They have also been linked to imbalances in the gut microbiota, which are associated with metabolic disorders such as anorexia, malnutrition, and obesity, as well as digestive tract damage like colon cancer [186]. The mistaken introduction of methanogens from the system for PF into the digestive system could lead to the progression of the mentioned illness.

CH_4_, the main result of carbohydrate fermentation by methanogens, has traditionally been believed to induce no harm in humans other than causing bloating. Yet, recent findings have associated the production of CH_4_ with the development of constipation and irritable bowel syndrome (IBS), as well as obesity [187].

Sulfate-reducing bacteria use sulfate for respiration instead of O_2_ in anaerobic conditions. The toxic H_2_S from sulfate-reducing bacteria has a significant impact on gut health by harming intestinal epithelial cells and causing DNA damage in human cells [188].

They are found naturally in O_2_-deprived marine and fresh H_2_O milieus and can generate hydrogen sulfide gas, which is harmful to humans and animals. SRB can cause corrosion of metals through the release of acidic compounds like H_2_SO_4_ [189].

Sulfate-reducing bacteria typically inhabit the human gut and play a role in inflammatory bowel diseases and colorectal cancers [190].

No dangers have been discovered in the literature concerning the effects of nitrate-reducing bacteria on humans or the environment.

The elevated levels of propionate may be attributed to the *Bacteroidetes* group, which are the primary producers of propionate in the human intestinal tract. In older adults, there is an increased presence of the *Bacteroidetes phylum* [191].

Propionate has the potential to function as a “metabolic disruptor”, which could elevate the chances of diabetes and obesity in humans. One of the primary focuses of the research was propionate, a short-chain fatty acid found in nature that aids in preventing the growth of mold on food products [192].

DF also generates waste streams containing high levels of volatile fatty acids (VFAs), which require costly treatment prior to being released [163].

Among biological methods for H_2_ extraction from biomass, DF is the most comprehensively understood and promising [193,194]. Though its yields are relatively low (up to 4 mol-H_2_/mol-glucose and 6 mol-H_2_/mol-sucrose), DF requires straightforward reactor designs, which makes it attractive for hydrogen production. Additionally, extensive studies have been conducted to enhance H_2_ production efficiency with hybrid systems involving DF, including microbial electrolysis cells (MECs), PF, anaerobic digester, and DF combined with a cell-free enzymatic system [195].

The DF process can be utilized to directly transform biomass-derived resources into H_2_ through either a one-stage or two-stage method. The most straightforward method is the one-stage AD process, which permits all stages (hydrolysis, acidogenesis, and acetogenesis) to occur within a single reactor. The hardest part of this approach is preserving the microbial balance throughout the entire process. The two groups of organisms vary considerably concerning physiology, nutritional needs, growth rates, and vulnerability to environmental influences, as previously noted [196]. A multistage process utilizes two or more reactors for digestion to distinguish the hydrolysis/acidogenesis and acetogenesis phases. Acidogenic bacteria produce organic acids and multiply faster than methanogenic bacteria. The multistage approach is implemented to enhance every phase of digestion, leading to more stable operations, a greater organic loading capacity, and heightened resistance to toxic substances and inhibitory compounds [197,198,199]. Consequently, in comparison to a single-stage method, this approach requires a higher initial investment and continuous operating costs.

The DF pathway can occur at temperatures between 26 and 40 °C, as in several studies presented in Table 3, or in hyper-thermophilic milieus (temperatures exceeding 70 °C). Due to the uncertain advantages of functioning outside mesophilic conditions (26–40 °C), the majority of DF operational research is conducted at approximately 35–37 °C [200]. At temperatures ranging from 29 to 70 °C, variability was noted to be greater during the decomposition of various biomasses and organic solid wastes [201]. To achieve optimal conditions during DF, researchers examined not only temperature but also various key environmental factors, including the pretreatment, pH, hydraulic retention time (HRT), organic loading rate (OLR), biomass composition, partial pressure, and type of reactor. On a broader level, the choice of raw materials is just as important. Due to their biodegradable substrates, the DF process emphasizes carbon sources that include monosaccharides, like glucose, and disaccharides, such as lactose or sucrose [67].

Numerous studies have highlighted the utilization of biomass and organic solid waste for H_2_ production. Nowadays, further investigation is required to identify the optimal reactor design, enhancements for microbial immobilization, innovative microbial strains, reductions in inhibitory effects, and a comprehensive fermentation process that might effectively convert biomass into H_2_ [202]. 

DF is in the developing stage and is characterized by a cost of 18.7 USD/kg. PF has drawbacks such as a low H_2_ yield and the necessity of a large reactor. The remedy for them is the requirement for solid-state anaerobic digestion (SS-AD), a cell-free enzymatic system, a microbial electrolysis cell, and an anaerobic digester. The process efficiency is in the range of 60–80% [177,178].

Table 3 displays details about the hazards associated with DF.

Kim et al. [203] achieved a cumulative H_2_ yield of 0.9 (mol H_2_/mol substrate) in a fermentation process utilizing sludge from an anaerobic digester of a waste-H_2_O treatment plant as an inoculum and food waste as a substrate at 35 °C, pH 5.3, and HRT 36 h (Table 2).

Likewise, Kumar et al. [204] achieved a cumulative H_2_ yield of 0.259 (mol H_2_/mol substrate) using *Escherichia coli* XL1-Blue/*Enterobacter cloacae* DSM 16657 as an inoculum and beverage waste H_2_O as a substrate at 37 °C and pH 6.5 (Table 2).

In a study by Yang et al. [205], a fermentative consortium MC 1 (composed mainly of *Firmicutes* and *Bacteroidota phyla*) was used as an inoculum along with food waste containing Fe-modified biochar as a substrate. The cumulative H_2_ yield achieved was 74.9 (mL H_2_/g vs) at 55 °C and pH 7.0. The ratio of inoculation was 10% by volume (Table 2).

Zhao et al. [206] achieved a cumulative H_2_ yield of 82.4 (mL H_2_/g TS) using activated sludge from a waste-H_2_O treatment plant and corn stover with thermally modified maifanite as a substrate at 35 °C (Table 2). 

Likewise, Chantawan et al. [207] achieved a cumulative H_2_ yield of 225.2 (mL H_2_/g vs) by utilizing anaerobic granules from a waste-H_2_O treatment plant’s digester as an inoculum and cassava processing wastes (cassava pulp and cassava processing waste H_2_O) as a substrate. The experiment was conducted at 35 °C, pH 6.0, and an HRT of 132 h (Table 2).

During their study, Kazemi et al. [208] achieved a cumulative H_2_ yield of 82.4 (mL H_2_/g TS) by using anaerobically digested sludge from a primary anaerobic digester as an inoculum and pruning wastes with food-rich MSW as a substrate. This was carried out at a temperature of 37 °C, pH levels of 5.0 and 7.0, and an HRT of 72 h (Table 2).

Likewise, Ban et al. [209] obtained a cumulative H_2_ yield of 101.8 (mL H_2_/g VS) by utilizing granular sludge from an upflow anaerobic sludge blanket that treated papermaking waste H_2_O as the inoculum and by using corn straw alongside excess sludge as the substrate, all at a temperature of 35 °C, a pH of 7.0, and an HRT of 17 days (Table 2).

In a subsequent study, Hussien et al. [210] achieved a cumulative H_2_ yield of 275.6 (mL H_2_/g vs) by utilizing anaerobic sludge from a digester and swine manure mixed with food waste as substrates under conditions of 35 °C, pH 5.5, and an HRT of 4 days (Table 2).

The dangers associated with using PF for extracting H_2_ from biomass are greater and more intricate compared to PF. Their reliance is influenced by the type of inoculum and substrate, as well as their level of interaction with the fermentation conditions.

Plant stalks, farming waste, and cheese waste are examples of substrates containing starch and cellulose that can be used to facilitate both DF and PF processes. Yet, their intricate composition prevents them from being directly used as substrates, so various technologies must be used to pretreat them before extracting fermentable sugars [211].

**Table 3:** Hazards related to fermentation types applied for H_2_ extraction from biomass during diverse studies.

Fermentation Type	Inoculum/Substrate/ Operating Conditions	Information of Hazards	Refs.
PF	*C. butyricum* and *R. palustris*; Rice straw; T: 30 °C; pH 7.0; LI: 6000 lux	*C. butyricum*—The presence of *Clostridium* bacteria in food products threatens human health and life. There are many poisonings and deaths due to the ingestion of *Clostridium* spp. toxins. The mitigation of hazards comprises the following measures: Regularly clean areas that may be contaminated with C. difficile. Wear protective clothing and gloves when it is essential to have direct skin contact with infected materials or animals. Eye protection should be worn when there is a recognized or possible risk of splash exposure. Any procedures that could generate aerosols or that entail high concentrations or substantial volumes must be performed within a biological safety cabinet (BSC). The use of needles, syringes, and other sharp instruments must be highly restricted. Extra measures should be considered when dealing with animals or large-scale operations.	[212,213]
		Some rare strains of *Clostridium butyricum* comprise the gene encoding the botulinal type E neurotoxin and promote hazards in certain types of food. The control of toxigenic *C. butyricum* in the food industry needs to allow for the great pH tolerance of this species. *Clostridium butyricum*—German TRBA Risk group: 2. (Agents that are associated with human disease that is rarely serious and for which preventive or therapeutic interventions are often available. They pose a medium risk to a person but a minimal risk to the community.)	[214]
		*R. palustris*’s metabolic effectiveness and growth rate are truly low. Very few genetic manipulation tools are achievable for *R. palustris* to raise its performance.	[215]
		Rice straw—Besides inducing air pollution, burning paddy straw leads to the loss of soil organic matter and essential nutrients, lowers microbial activities, and makes the land more vulnerable to soil erosion. Mitigation of hazards: Avoid burning paddy straw.	[216]
		Post-harvest straw is often burned, releasing several pollutants into the environment. CO_2_ dominates at 70%, accompanied by CH_4_ at 0.66%, CO at 7%, and N_2_O at 2.09%. Mitigation of hazards: Avoid burning post-harvest straw.	[217]
		*Rhodopseudomonas palustris*—German TRBA Risk group: 1. (Agents that are not associated with disease in healthy adult humans. This group has a record of animal viral etiologic agents in common use. They represent no or little risk to an individual and no or little risk to the community).	[218]
	*R. pseudopalustris* DSM 123; Tequila vinasses; T: 30 °C; pH 7.0; LI: LED lamp (13.5 W/m^2^); Inoculum: 3.3 g/L cell suspension	*Rhodopseudomonas pseudopalustris* (DSM 123)—German TRBA Risk group: 1.	[215]
		*Tequila vinasses* pose a significant threat to surface aquatic ecosystems when discharged without proper treatment or with insufficient treatment. The disposal of highly concentrated *Tequila vinasses* poses an ecological risk. Mitigation of hazards: Utilizing AD methods to decrease organic material while generating BG.	[219,220,221]
		Improperly disposing of untreated *Tequila vinasses* (TVs) can result in significant environmental harm to soil and H_2_O sources and the production of elevated levels of GHG emissions. Using TV for field fertilization may not always be effective since not all the nutrients (N, P, K) on TV are readily available for crops. Mitigation of hazards: TV adjustment to crop parameters.	[222]
	HAU-M1 (*R. sphaeroides* (9%), *R. palustris* (28%), *R. rubrum* (27%), *R. capsulata* (25%) and *R. capsulatus* (11%); Corn stover; The 1st study: pH 7.0; LI: 3000 lux; Inoculum: 30% 150 mg/g TS; TiO_2_/AC fiber addition of 100 mg/L. The 2nd study: T: 30 °C; pH 7.0; LI: 3000 lux; Substrate concentration: 25 gDM/L.	*Rhodobacter sphaeroides*, *Rhodopseudomonas palustris*, *Rhodospirillum rubrum*, and *Rhodopseudomonas capsulata*—German TRBA Risk group: 1.	[215]
		Corn straw roots can easily absorb Cu and Zn in the soil, which can be harmful to human health, especially to children. The levels of heavy metals in soil and flue gas from burning corn straw have reached a very high ecological risk. The mitigation of hazards comprises the following measures: Systematic control of samples of corn straw roots. Avoid burning corn straw.	[223]
		When crop straw is added to PAH-contaminated agricultural soils, particularly corn straw, the accumulation of polycyclic aromatic hydrocarbons (PAHs) in winter wheat, as well as the ecological and human health risks, seems to decrease due to increased PAH dissipation in the rhizosphere soil.	[224]
	*R. sphaeroides* NCIMB8253; Combination of palm oil (25%, *v*/*v*), pulp and paper (75%, *v*/*v*) mill effluents; T: 30 °C; LI: 7000 lux; Combined substrate (25 vol.% POME and 75 vol.% PPME).	*Rhodobacter sphaeroides*—German TRBA Risk group: 1.	[215]
		Inhaling palm oil could be dangerous. It can cause irritation of the respiratory tract and skin. Absorbing it via the skin could be dangerous. The mitigation of hazards comprises the following measures: Relocate to an open area if vapors or decomposition products are accidentally inhaled. In case of skin contact, rinse thoroughly with soap and abundant H_2_O. In case of eye contact, rinse the eyes with a specialized eyewash solution. The consumption of safe vegetable oil is non-harmful. Make sure there is sufficient dry storage space with good ventilation, maintained between 10 and 50 degrees C. Address any spills right away. Avoid open flames, hot surfaces, and ignition sources.	[225]
		Palm oil contains high levels of saturated fat, which can increase LDL cholesterol levels and increase the likelihood of developing heart disease. The oil may be associated with inflammation, specific cancer risks, and type 2 diabetes. It is damaging to the environment. The sector has a record of unsustainable farming methods, is known for unjust labor practices, and has led to extensive deforestation.	[226]
		Palm oil, often grown on plantations after rainforests are flattened and burned, is environmentally destructive. Following some initial advances, the reemergence of deforestation linked to palm oil production in Indonesia is evident once more.	[227]
		The pulp and paper industry uses a significant quantity of energy and releases pollutants and GHGs into the atmosphere. The waste produced by the pulp and paper industry causes significant damage to aquatic life, disrupts the food chain, and leads to various health issues. This garbage creates significant issues for both H_2_O- and land-dwelling creatures. Health risks associated with waste H_2_O range from skin irritation to genetic abnormalities. Toxic substances in waste H_2_O demonstrate mutagenic and genotoxic effects. Mitigation of hazards: The waste from the pulp and paper industry must be directed (under strict control) to appropriate treatment plants.	[228]
DF	Sludge from an anaerobic digester of a waste-H_2_O treatment plant; Food waste; T: 35 °C; pH 5.3; HRT: 36 h	Toxic substances in the AD system, whether from influent waste streams or bacterial metabolism, can hinder the digestion process. Mitigation of hazards: Systematic control of sources of consumed substances.	[229]
		Wasted food releases damaging gases like CO_2_, H_2_S, CH_4_, N_2_O, and PM2.5, which are harmful to human health. Mitigating hazards: Provide necessary ventilation for the zone with people present.	[230]
	*E. Coli* XL1-Blue/*Enterobacter cloacae* DSM 16657; Beverage waste H_2_O; T: 37 °C; pH 6.5	*E. coli* XL-1-Blue—no known hazards.	
		*Enterobacter cloacae*—There is a risk to immunocompromised patients when in direct or indirect contact with contaminated persons/objects. Pathogens can be transmitted via contaminated infusion solutions or blood products. Mitigation of hazards: Avoid contact with contaminated people. Use proper protective clothing, gloves, masks, and glasses.	[231]
		*Enterob. cl.*—a biosafety level 1 organism in the USA/level 2 in Canada.	[232]
		*Enterobacter* infections are serious ones with a high mortality rate, even with appropriate treatment. Enterobacter species induce many nosocomial infections and, less frequently, community-acquired infections, including urinary tract infections (UTIs), respiratory infections, soft tissue infections, osteomyelitis, and endocarditis, among many others.	[233]
		Waste H_2_O in the beverage industry contains raw materials used in beverage fabrication, such as diverse sugars, ethanol, fruit concentrates, malts, hops, syrups, acids, and mineral salts. Due to the raw materials used in beverages, the concentration of organic matter is high in the waste H_2_O. Mitigation of hazards: Waste H_2_O from the beverage industry must be directed (under strict control) to appropriate treatment plants.	[234]
	Fermentative consortium MC 1 (*Firmicutes* and *Bacteroidota phyla*); Food waste + Fe-modified biochar; T: 55 °C; pH 7.0; Inoculation ratio: 10 vol.%	*Firmicutes*—A higher ratio of Firmicutes is tied to Type 1 and Type 2 diabetes, heart disease, certain cancers, Alzheimer’s, and obesity.	[235]
		*Firmicutes* and *Bacteroidetes* can influence diseases related to obesity, which are also risk factors for breast cancer. It may be necessary to adopt an appropriate diet and change your lifestyle habits toward being more active.	[236]
		Unsafe waste can contaminate the soil, H_2_O, and air, disrupting ecosystems and harming wildlife. The mitigation of hazards comprises the following measures: Limit unsafe waste production. Store such waste only in designated zones.	[237]
		Human exposure to unsafe waste can lead to acute or chronic health issues, ranging from respiratory problems to cancer. Mitigation of hazards: Avoid contact with unsafe waste.	[238]
		Food waste—1/3 of all human-caused GHG emissions and 8% of GHG annually, a significant waste of fresh H_2_O and ground H_2_O resources. Emissions from food waste, like H_2_S, CH_4_, and volatile organic carbons, can affect human endocrine, respiratory, nervous, and olfactory systems. The mitigation of hazards comprises the following measures: Avoid food waste. Provide enough ventilation in zones with people present.	[230]
		Fe-modified biochar feedstock and temperature mainly affect biochar (BC) contamination and toxicity. PAHs, heavy metals, pH, and EC affect BC toxicity. Mitigation of hazards: Systematic control of biochar parameters.	[239]
		Biochar is a flammable material (H228). After inhalation (H333) or swallowing (H305), it may be harmful. It induces eye irritation (H320). If heated, it may induce a fire (H242). Excessive C dust from handling biochar may produce allergenic responses in a few sensitive individuals. Overexposure to biochar dust may cause skin/eye and upper respiratory tract irritation, allergenic responses, and asthma. The mitigation of hazards comprises the following measures: Avoid heat/sparks/open flames/hot surfaces and do not smoke (P210). Refrain from inhaling dust (P261). Utilize personal protective gear as needed (P281). Do not keep the material under conditions with moisture levels under 10% or at temperatures exceeding 400 °C. Suitable personal protective gear should be used to reduce irritation caused by dust.	[240]
		The production of *Pinus patula* raw biochar—the source of energy utilized during this process—accompanied by the generation of gases and polycyclic aromatic hydrocarbons. For Fe-modified biochar, the potential environmental effects differed only in the stage of biomass modification with the metal. They depend on the extraction of Fe and the generation of waste H_2_O.	[241]
	Sludge from waste-H_2_O treatment facility; Corn stover+ therm.-modified maifanite; T: 35 °C	Corn stover—as mentioned	
		Thermally modified maifanite—no hazards found.	
		The MicroStart waste-H_2_O bacterial inoculum is harmful if swallowed. It can induce harm if it contacts the skin or is breathed in. It may induce eye irritation. The mitigation of hazards comprises the following measures: Use gloves for protection/clothing for protection/eye safety/face safety. If ingested, provide plenty of H_2_O to dilute. Seek medical advice without delay. If on the skin or clothes, promptly rinse the skin with H_2_O. If discomfort continues, consult a healthcare professional. If in the eyes, promptly rinse them thoroughly with H_2_O for a minimum of 15 min. If discomfort continues, consult a medical professional. Avoid extreme temperatures. Steer clear of potent acids and alkaline substances. Steer clear of carbon and nitrogen oxides. Disposal must adhere to federal, state, and local regulations.	[242]
	Anaerobic granules from an anaerobic digester of a waste-H_2_O treatment plant; *Cassava* pulp and processing waste H_2_O; T: 35 °C; pH 6.0; HRT: 132 h	Organoarsenic feed additives (roxarsone) induce organoarsenicals in livestock waste H_2_O and anaerobic waste-H_2_O treatment systems.	[243]
		AD—as mentioned.	
		Cassava production worsens soil fertility via crop removal of nutrients, a more serious and long-term effect on environmental erosion. Mitigation of hazards: Limit areas for cassava production.	[244]
		*Cassava* leaves contain toxins from cyanogenic glucosides (which may cause cyanide poisoning, resulting in signs like headaches, nausea, dizziness, diarrhea, vomiting, and potentially fatal outcomes) as well as antinutritional components (such as high fiber, tannins, polyphenols, and phytic acid) that lower the absorption and digestion of nutrients. Mitigation of hazards: Avoid eating improperly prepared *Cassava* leaves.	[245]
		Improperly cooked *Cassava* may have substances that change into cyanide in the body. This could induce cyanide poisoning and result in specific paralysis conditions. Some individuals may experience an allergic response to cassava. Mitigation of hazards: Follow good *Cassava* cooking practices.	[245]
	Sludge from primary anaerobic digester; Pruning wastes + food-rich MSW; T: 37 °C; pH 5.0 and 7.0; HRT: 72 h	AD—as mentioned.	
		Burning waste from tree pruning outdoors decreases air quality and adds to the greenhouse effect. Mitigating hazards: Avoid burning waste from tree pruning.	[246]
		Food waste—as mentioned	
	Sludge from upflow anaerobic sludge blanket treating papermaking waste H_2_O; Corn straw + excess sludge; T: 35 °C; pH 7.0; HRT: 17 days	Toxic pollutants in papermaking waste H_2_O have carcinogenic, genotoxic, and mutagenic effects. Gaseous compounds are toxic to human health and the environment. The mitigation of hazards comprises the following measures: Waste H_2_O from the paper industry must be directed (under strict control) to appropriate treatment plants. Provide enough ventilation for zones with people present.	[247]
		Corn straw—as mentioned.	
		Anaerobic sludge—A problem with AD occurs since the feedstock may contain heavy metals or persistent organic pollutants (POPs). Heavy metals cannot be destroyed by digestion. The mitigation of hazards comprises the following measures: Systematic control of AD parameters. Limit its contact with people. Proper personal protective gear should be used.	[248]
	Anaerobic sludge from an anaerobic digester; Swine manure + food waste; T: 35 °C; pH: 5.5–6; HRT: 4 days	AD—as mentioned.	
		The concentration of Cu in swine manure exceeded the Cu limit; likewise, the concentration of Zn in swine manure exceeded the Zn limit. All livestock manure showed elevated levels of Zn, Cu, and Cr, suggesting possible ecological hazards. Swine manure exhibits the maximum potential ecological risk for agronomic application. Swine manure poses a non-carcinogenic risk to kids and an unacceptable carcinogenic risk to kids. The mitigation of hazards comprises the following measures: Swine manure should be stored only in determined zones. Limit its contact with people. Proper personal protective gear should be used. The swine manure content must be controlled before its application to the soil.	[249]
		Food waste—as mentioned.	

Abbreviations: Volatile solids (VSs), dry matter (DM), Total Solids (TSs), Palm Oil Mill Effluent (POME), Pulp and Paper Mill Effluent (PPME), hydraulic retention time (HRT).

No matrices of risks related to any solutions for hydrogen extraction from biomass using the dark fermentation process were found in the literature. Nevertheless, Lubentsov et al. [250] presented the choices and rationale for priority control tasks employed in the automation of biogas plants (BPs), considering the risk of losing control. The various kinds of technological risks involved in managing fermentation processes in methane tanks were examined, resembling DF utilized for H_2_ extraction from biomass. The authors performed a well-supported examination of quantitative risk evaluations by employing the pairwise comparison technique. Utilizing the expert opinion consistency method within an algorithm for addressing the selection and justification problem enabled a thorough analysis of the coherence of expert opinions and helped establish whether the resulting estimates were random. Utilizing the acquired data models for the anaerobic fermentation process parameters, the rationale for creating an improved control system for optimal substrate heating temperature, temperature regulation within the methane tank, and efficient timing for the conclusion of the fermentation process was established.

H_2_ yields in PF and DF depend on the inoculum, temperature, pH, and substrate type and concentration [39]. Such effects are presented in Table 4.

**Table 4:** Effects of the main parameters on H_2_ yield in PF and DF.

Parameters	Effect on H_2_ Yield in PF and DF	Refs.
Inoculum	In PF, incorporating purple non-S bacteria such as *Rhodobacter* sp. and increased light intensity can boost H_2_ production, while using mixed strains can further enhance the yield. In DF, H_2_ production relies on the use of strict (e.g., *Clostridium* sp.) or facultative anaerobic bacteria and can be improved through methods like immobilization and the addition of metal ions or oxide nanoparticles (NPs).	[155]
Temperature	The fermentative bacterial community generates H_2_ across a broad temperature spectrum, with mesophilic (35–40 °C) and thermophilic (50–60 °C) conditions frequently utilized due to their effects on pH and VFA generation.	[251]
pH	In PF, an acidic pH promotes H_2_ production, while in DF, a nearly neutral pH is more effective for H_2_ generation. Raising the pH might improve the capacity of H_2_-generating bacteria; nonetheless, elevated pH levels could lower H_2_ production.	[252,253]
Type of substrate	The selection of the substrate is based on factors like expense, accessibility, carbohydrate levels, and ease of fermentation. Although glucose is widely used, solid waste and industrial waste H_2_O present attractive options for economic and sustainability factors, with little pretreatment, affecting the best substrate choice for H_2_ generation.	[254]
Substrate concentration	Optimal H_2_ production is positively associated with the substrate concentration, ensuring adequate nutrition for photosynthetic bacteria to sustain H_2_ production.	[175]
	In PF, a high substrate concentration can greatly elevate butyric acid levels, which reduces the pH and halts H_2_ production.	[255]

Research conducted by Salma Aathika and the team also confirms that the appropriate selection of process parameters allows for an increase in H_2_ yield. The optimization of the waste mixing ratio, pH, and temperature resulted in a 92.3% reduction in volatile solids, while the H_2_ yield itself increased eightfold [256].

#### 4.1.4. Integrated Systems (ISs)

DF exhibits a high H_2_ generation rate but a low yield, whereas PF and microbial electrolysis cell technology have a slower generation rate but a higher yield. Hence, it is recommended to incorporate varied technologies [257].

Combining DF and PF processes enhances efficiency and sustainability in H_2_ production. This integration can be accomplished in one step by co-cultivating H_2_-producing bacteria in DF with photosynthetic bacteria in PF or in two steps by using the DF effluent as a substrate for the PF process, raising renewable-H_2_ generation. Because the single-step process has low yields, the two-step process is preferable. In the second system, in the first step, glucose is broken down by DF bacteria to produce H_2_ and CH_3_COOH acetic acid as intermediate products (Formula (7)), which are then transformed to H_2_ by PF bacteria in step II (Formula (9)) [89].

(9) $$2C_{2}H_{4}O_{2}+4H_{2}O\overset{Light}{\to }8H_{2}+4CO_{2}$$

**Figure 6:**
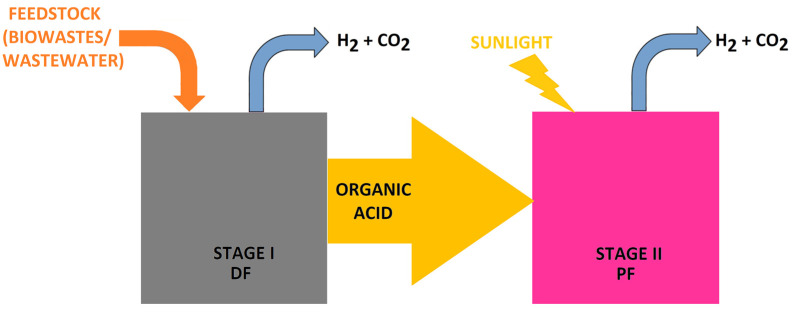
A two-stage bioreactor with integrated DF and PF systems.

Theoretically, in pure DF with glucose as the substrate, it is possible to achieve a H_2_ yield of up to 12 mol H_2_/mol substrate, with CH_3_COOH being the main metabolite [163]. Nevertheless, tweaking the concentration, composition, and pH of the effluent produced during DF, as well as the selection of PF bacteria, is crucial for achieving maximum generation efficiency. A two-stage bioreactor with integrated DF and PF systems is shown in Figure 6.

To summarize the pros and cons of the biological method used for H_2_ extraction from biomass (Table 5—based on [39]), it can be noted that DbP exhibits excellent energy efficiency, achieving as much as 12.2% in green algae [258]. In comparison, DbP usually exceeds i-DbP (maximum efficiency 4.1%), PF (maximum efficiency 8.5%), and DF (maximum efficiency 12.0%) [182,258]. Although DbP is efficient, the O_2_ generated during PSII significantly inhibits hydrogenases, subsequently decreasing H_2_ [259]. Mitigation methods like inert gas purging, O_2_ production inhibitors (e.g., Cu), and S scarcity have been investigated [260,261].

i-DbP provides benefits including renewability, sustainability, scalability, and the ability to integrate with current infrastructure. Nonetheless, it faces issues with low efficacy, the restricted availability of catalysts, sensitivity to the environment, and technological hurdles, requiring additional research and development for enhanced performance and cost-effectiveness [81,262,263].

DF is a recognized technique for producing renewable H_2_, utilizing various substrate sources and functioning continuously without light. It guarantees consistent and dependable hydrogen generation at a reduced price [264]. However, its effectiveness in transforming substrates into H_2_ is restricted by competition from H_2_-utilizing microorganisms in the mixed microflora. These consist of homo-acetogens, hydrogenotrophic methanogens, sulfate-reducing bacteria, nitrate-reducing bacteria, and propionate producers, which lower overall H_2_ production. Additionally, DF produces effluents high in volatile fatty acids (VFAs), requiring expensive treatment prior to release [163]. Incorporating other biological techniques such as PF presents possible ways to improve total hydrogen production and address effluent issues. In this regard, integrated systems that incorporate DF and PF demonstrate promise for enhancing H_2_ production via synergistic interactions [158]. Nevertheless, additional research and technological progress are essential to improve their efficiency and scalability, given that deploying integrated systems can be costly and demand specialized infrastructure, which are major drawbacks [161].

Additionally, Ghasemi et al. [32] noticed that certain studies have utilized microbes to aid in the biological transformation of biomass, primarily through dark fermentation methods. The primary advantages are discovered in situations of moderate use. The slow conversion rate and low production volumes of these technologies are the main constraints hindering progress. The capital expenses of these systems are elevated due to the requirement for expensive bioreactors and separation methods. Pretreatments are necessary for biomass that resists treatment, as this leads to the production of inhibitors and reduced operational and capital expenses. The primary objectives of future developments should be the development of new bacterial strains, improved bioreactors, and localized small-scale production facilities. Economic limitations to PF consist of higher yields accompanied by elevated energy expenses. To address these challenges, metabolic engineering can allow for considerable progress in the bio-H_2_ process. The effects of nutrient limitations and substrate utilization were examined to recognize genes in microalgae that enhance H_2_ production. The design of photobioreactors needs to be optimal. The inhibitory substances present during pretreatment create a significant barrier, representing one of the main technical challenges of integrated DF and PF. The substrate restricts either or both of the processes. The elevated feedstock expenses, the processing costs caused by the toxicity of waste-H_2_O treatment byproducts, the operation and upkeep of the sequential reactor, and the operational costs incurred during the pretreatment of DF effluent constitute further economic obstacles to this approach. 

#### 4.1.5. Projects Focused on Biological Technologies

The composition and attributes of biomass are influenced by various feedstocks that have different levels of carbohydrates, lipids, proteins, and lignocellulosic substances, with each necessitating particular pretreatment and conversion techniques. The first steps consist of deconstructing intricate biomass into basic sugars, which are subsequently fermented to produce hydrogen. Various factors, including substrate levels, microbial metabolism, and enzyme activity, can influence the effectiveness and rate of these processes [265]. 

Choosing microorganisms like bacteria and archaea is essential. Some microbes possess the natural capability to generate hydrogen from biomass, whereas others might require genetic alterations or co-culturing with different organisms. Thermophilic species flourish at elevated temperatures, whereas mesophilic species favor temperate conditions [266]. The pH level of the medium influences microbial activity and product yield, as various microbes thrive in specific pH ranges [265].

The design of the bioreactor, which encompasses a continuous stirred-tank reactor or an anaerobic baffled reactor, and its operational parameters, like gas removal, mixing, and retention time, are essential elements that influence the scalability and effectiveness of biomanufacturing procedures [265].

Utilizing the microalga *Chlorella* sp., H_2_ was generated by Giang et al. [267] via DF and anaerobic solid-state fermentation. The result was 16.2 mL/g of volatile solid (VS, or total organic material in the biomass). Pre-hydrolysis combined with simultaneous saccharification and fermentation enabled the attainment of up to 172 mL/g VS in production and 2.4 mL/g VS·h in productivity. Additionally, Wang and Yin [268] reported H_2_ production from various forms of biomass microalgae. The pretreatment enhanced the yield (reaching 958 mL H_2_/g VS from *Chlorella* sp. with heat pretreatment using HCl) through the disruption of the microalgal cells. Nevertheless, as the procedures have not reached the industrial scale, additional research is needed to enhance their feasibility.

Multiple strategies can improve this process, such as refining pretreatment methods, expanding operations, incorporating cutting-edge technologies, etc. A possible solution is to collaborate with different organizations that can support commercialization. The goal is to enhance productivity and hydrogen output while minimizing environmental effects. Whereas other studies focused exclusively on crop residues, Kumar et al. [269] assessed the production of hydrogen from various biomass types (lignocellulosic, waste H_2_O, and algae) through the dark fermentation process. The research evaluated different pretreatments for lignocellulosic biomass, such as alkali pretreatment to isolate lignin and generate fermentable sugars through enzymatic hydrolysis using cellulase and xylanase. The highest yield was achieved under slightly acidic conditions (pH 4.8, 0.2% HCl) utilizing 176 mL H_2_/g of cornstalk with cellulase.

Integrating various pretreatment techniques and focusing on affordable enzymes will enhance efficiency and address the expensive components of this process [265]. 

In a study, the C/N ratio, phosphate concentration, and substrate concentration for photo-fermentation (PF) were modified to develop a one-step approach that employs *Rhodobacter sphaeroides* to produce hydrogen from straw biomass. Initially, the biomass underwent hydrolysis in an acidic condition for 30 min at 118 °C using 5% HCl. Two *Rhodobacter sphaeroides* strains, HYO1 (wild type) and WHO4 (mutant), transformed the hydrolyzates in a balanced environment. Thanks to pH stabilization, they achieved a higher yield (for the WHO4 strain, the reducing sugar was 4.62 mol/mol) compared to what they could have obtained with direct sugar PF [270,271,272]. This streamlines the standard multistep procedure, thereby lowering expenses, intricacy, and material consumption. Nonetheless, options other than HCl can be investigated to enhance environmental sustainability and further reduce costs related to the acid for hydrolysis when considering scaling up for industrial applications [265]. 

**Figure 7:**
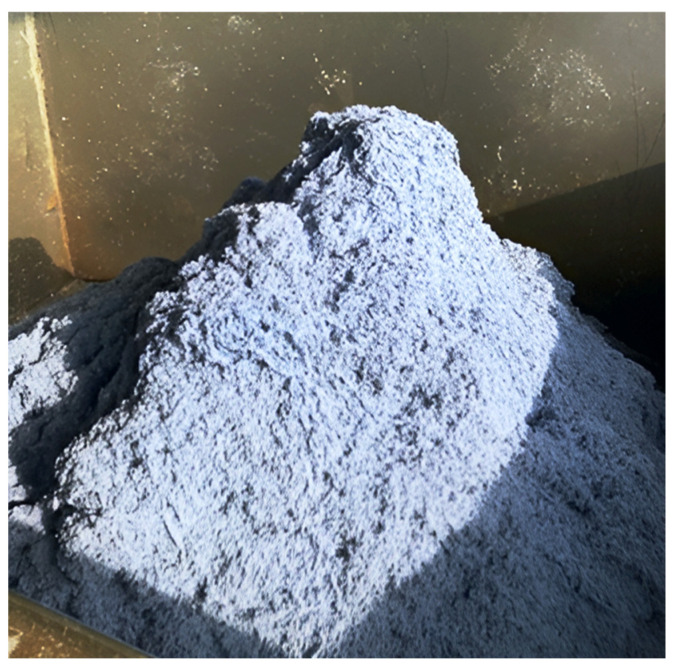
Chrome shaving waste—main substrate in dark fermentation.

As mentioned above, an essential aspect of DF is the selection of substrates. Substrates rich in starch and cellulose facilitate the progress of this process. A surprising yet promising substrate with potential for use in DF is waste from the leather tanning process—shaving waste. Shaving wastes are produced during the shaving process of the flesh side of preliminarily tanned leather to reduce its thickness. They appear as small shavings, as shown in Figure 7. This previously underutilized waste material presents a potential source of bio-H_2_ in the DF process.

**Figure 8:**
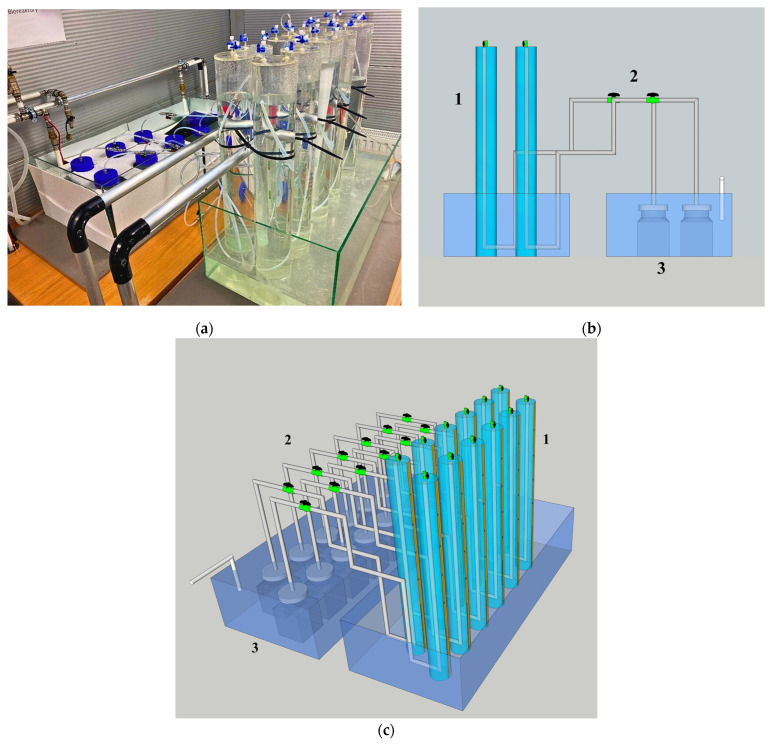
The installation used for the DF process: (**a**) Actual appearance of the installation; (**b**) installation scheme—plane view; (**c**) installation scheme—perspective view. 1. System to perform gas measurement; 2. A gas flow system from the reactors to the measurement system; 3. reactors with a temperature control system.

The installation for studying the DF process of tannery waste at a laboratory scale is a specially designed setup that allows for continuous gas production measurement and maintains stable temperature conditions. The schematic of this setup and its actual version are given in Figure 8.

**Figure 9:**
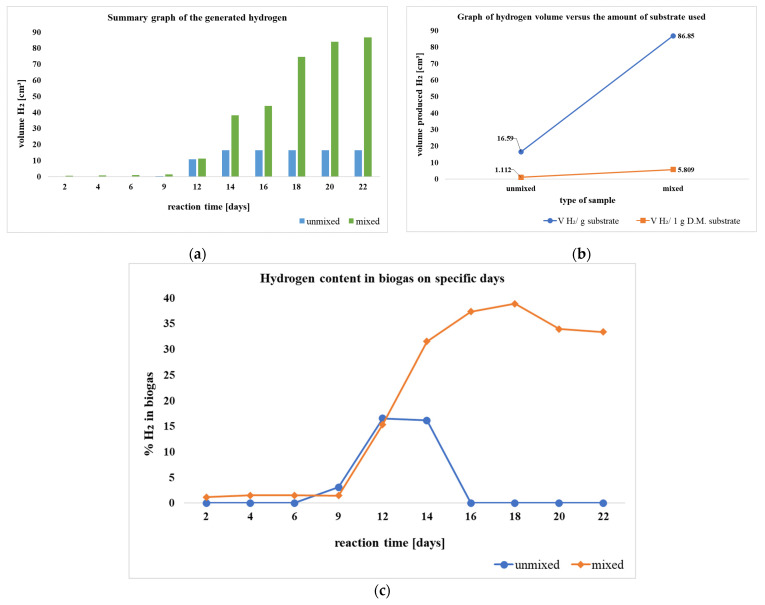
A comparison of the efficiency of generated H_2_ and the H_2_ content in BG in an experiment with mixing: (**a**) a summary graph of the generated H_2_; (**b**) a graph of H_2_ volume versus the amount of substrate used; (**c**) H_2_ content in BG on specific days.

Additionally, the DF process was intensified by the mixing process. The PF process was conducted at a temperature of 50 °C and a pH of 5.0. The mixing was carried out using a magnetic stirrer and manual mixing. Magnetic stirring was performed for 1.5 h per day, and manual mixing was performed twice for 5 min each per day. Mixing improves the homogeneity of milieu conditions and improves contact between microorganisms and the substrate. Preliminary results from the DF of chromium shaving waste with glucose addition clearly indicate that the mixing process can increase the H_2_ yield several times. Figure 9 shows a comparison of the total H_2_ yield in DF with and without mixing. The data indicate that, in the case of mixing, 5.81 cm^3^ of _H2_ was produced per 1 g of dry matter (DM) of the substrate, while in the process without mixing, only 1.112 cm^3^ of H_2_ per 1 g of DM was produced. The H_2_ concentration in BG is also more than twice as high for the DF process with mixing compared to the process without mixing. The mixing process contributes to a longer and more stable PF process by ensuring a constant supply of nutrients to microorganisms.

**Figure 10:**
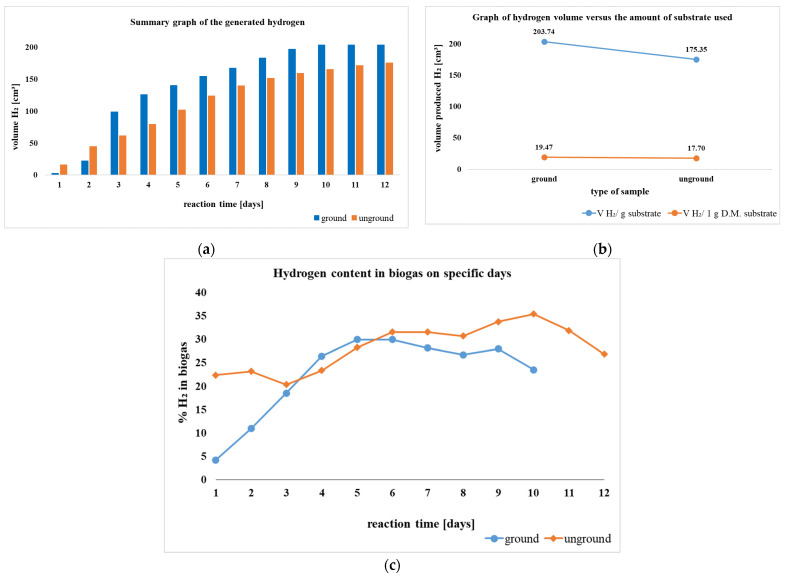
A comparison of the effectiveness of generated H_2_ and the H_2_ content in BG in an experiment with grinding: (**a**) a summary graph of the generated H_2_; (**b**) a graph of H_2_ volume versus the amount of substrate used; (**c**) H_2_ content in BG on specific days.

The process effectiveness is also affected by the method of substrate pretreatment. The pretreatment can include grinding, granulation, or chemical or thermal treatment. In the case of grinding substrates as a pretreatment method, a mixture of chrome shaving waste and a co-substrate in the form of husks (from buckwheat, variety VI) was ground to a powder below 1 mm. The process was conducted at a temperature of 50 °C and a pH of 5.0. The results presented in Figure 10 indicate that grinding increases the H_2_ yield by nearly 2 cm^3^ per 1 g of dry matter (DM) of the substrate. Reducing the substrate to a powder form allows for a 7% increase in H_2_ production efficiency in terms of the total H_2_ yield.

The conducted research on DF using Cr shaving waste confirms the potential of utilizing new substrate sources, such as the leather industry. The proper selection of parameters and the application of additional processes allow for a multifold increase in the H_2_ yield.

### 4.2. Thermochemical Methods for H_2_ Extraction from Biomass and Hazards Related to It

TC methods used for H_2_ extraction from biomass include PY, hydrothermal (HT) processes, and GA. The GHG emission intensities of biomass combustion systems (0.25–0.30 kg CO_2_-eq/MJ) highly exceed those of biomass GA systems (0.02–0.14 kg CO_2_-eq/MJ) and of the biomass PY systems (0.012–0.1 kg CO_2_-eq/MJ) [273]. For the comparison, the life cycle equivalent CO_2_ emissions for fuels utilized for transport equal 0.073 kg CO_2_-eq/MJ for 100% mineral petrol, 0.075 kg CO_2_-eq/MJ for 100% mineral diesel, and 0.065 kg CO_2_-eq/MJ for LPG [274]. Inexpensive and simple biomass combustion can provide heat for the other techniques used for biomass processing; however, it is in the developing stage. Its main drawback is the generation of CO_2_ and CO. The remedy for this is the requirement for post-combustion separation and CO_2_ storage [275].

According to OSHA, CO_2_ is considered dangerous at levels of 5,000 ppm (0.5%) during an 8 h work shift. Levels exceeding 40,000 ppm (4%) may pose a risk to life. Indicators of excessive exposure encompass headaches, lightheadedness, breathlessness, and disorientation. Elevated levels can replace O_2_, resulting in a lack of atmospheric O_2_. In limited areas, the buildup of CO_2_ is significantly dangerous. Because it is denser than air, it moves along the ground and accumulates in ditches and cellars. During a release, CO_2_ may create a noticeable vapor cloud, elevating the chances of injuries.

Effective strategies for CO_2_ management involve adequate ventilation, ongoing monitoring, and prompt response protocols, greatly improving safety throughout operations. When managing CO_2_, it is essential to recognize its possible dangers. The management of CO_2_ necessitates the use of personal protective gear, including gloves, goggles, and face shields. When elevated CO_2_ levels can occur, workers should employ proper CO_2_ sensors and alarm systems [276].

#### 4.2.1. Biomass Pyrolysis

PY resembles GA, but it can function at reduced temperatures and needs no oxidizing agent [277]. In certain situations, a small amount of an oxidizing agent may be used to improve heat generation [278]. During PY, biomass is heated in an O_2_-less milieu or partially combusted with a poor O_2_ supply, resulting in a hydrocarbon-rich gas mixture, an oil-like liquid, and a carbon-rich solid residue.

PY involves breaking down biomass into solid, liquid, and gas parts using heat in a non-reactive milieu [279]. In the PY process, the complex hydrocarbon compounds derived from biomass are transformed into simpler molecules through various reaction pathways. Initially, hemicellulose, a component of biomass, breaks down at temperatures between 220 and 315 °C, leading to the production of CH_3_COOH, various organic acids, sugars, and furans. Cellulose decomposition occurs between 315 and 400 °C, leading to the production of levoglucosan and other anhydrocelluloses [280]. 

Lignin, consisting of several aromatic rings, breaks down slowly, with decomposition occurring across a wide temperature spectrum (200–900 °C), producing various compounds, like oligomers, polysubstituted phenol monomers, H_2_, and CH_4_, as the main outputs [281].

PY can be carried out between 400 and 800 °C. Depending on the temperature, heating rate, and residence time, PY can be classified as slow, fast, or ultra-fast. Equation (10) outlines the overall pyrolysis process [282].

(10) $$Biomass+Heat\to H_{2}+CO+CO_{2}+CH_{4}+H_{2}O+Bio-oil+Char$$

Enhancing biomass characteristics, temperature, heating rate, residence time, and catalyst usage can improve the obtaining of H_2_ via PY [283].

A comprehensive review of biomass PY, including conventional and advanced technologies, reactor designs, product compositions, and yields, as well techno-economic aspects, was published by [284]. Based on the character of the biomass motion in the reaction zone, PY reactor types belong to the following categories: pneumatic bed reactors, gravity, stationary beds, and mechanical reactors. In particular, the development of hybrid reactors integrating multiple PY techniques can enhance product yields while lowering operational costs.

Various advanced biomass pyrolysis technologies are still being developed, including co-pyrolysis, catalytic pyrolysis, microwave pyrolysis, hydrothermal pyrolysis, and plasma pyrolysis. Such technologies differ to varying degrees in their mechanisms, advantages, and potential applications. Very promising is the integration of catalytic and co-pyrolysis methods [284].

Scaling up pyrolysis technologies from laboratory to industrial scales requires ensuring techno-economic feasibility and adaptation to regulatory issues with large-scale operations [284].

The composition and yield of pyrolysis products are affected by the type of biomass, heating rate (HR), hot vapor residence time (HVRT), solid residence time (SRT), particle size (PS), temperature (T), and type of reactor used [284].

Slow PY (carbonization) is mainly used to produce char as the primary product (yields in a range from 30 to 40 wt.%), along with bio-oil and non-condensable gases as secondary products. It occurs at temperatures ranging from 300 °C to 900 °C, with a heating rate (HR) of less than 0.4 °C/s and an extended solid residence time (SRT) of up to 12 h. The hot vapor residence time (HVRT) can vary from 1 s in reactors with a N_2_ flow during pyrolysis to 7200 s when N_2_ is solely utilized to eliminate air from the reactor prior to PY. The particle size (PS) of biomass employed in slow pyrolysis may vary between 0.075 and 19 mm. As this PY has the greatest SRT, a substantial PS is not an issue. All forms of biomass (irrespective of moisture levels) can be utilized in slow PY [284]. Performing slow PY at temperatures lower than 450 °C results in a significant charcoal content [67].

When N_2_ gas is stored in a pressurized tank, it can explode when exposed to heat. N_2_ may replace O_2_ and lead to quick suffocation [285]. The solution approach is similar to that of O_2_. 

Intermediate PY is characterized by a moderate heating rate (0.4–10 °C/s), brief heating times (0.5 to 40 s), reaction temperatures between 350 and 700 °C, and solid residence times ranging from 1.2 to 78 min. This procedure usually results in a liquid output of 40–50 wt.%, along with non-condensable gases and solid char, attaining values between those of the outcomes of slow and fast PY. The bio-oil produced via intermediate PY is notable for its lower viscosity and minimal tar content, predestinating it for immediate thermal applications. In addition, the generated char is dry and fragile, making it suitable for biofertilization and gasification. This approach likewise accommodates a diverse range of feedstocks, such as woody biomass, straws, grasses, and agricultural byproducts, without requiring significant grinding. The adaptability of intermediate PY enables the processing of larger feedstock sizes and contaminated biomass, increasing its usability [284].

H_2_ generation can be realized by fast or flash PY at high temperatures and appropriate residence time [286].

Fast PY enables the swift transformation of biomass into bio-oil, charcoal, and gas products. This method consists of heating the biomass to temperatures between 300 and 1400 °C at high heating rates of 10 to 1000 °C/s, with an extremely short high-velocity reaction time of 0.1 to 12 s and a solid residence time of 0.017 to 10 min. The main goal of fast pyrolysis is to enhance the production of bio-oil, which usually accounts for 50–75% by weight of the output, greatly exceeding the yields of char and gases. These yields are attained by raising the biomass to a temperature where thermal cracking takes place while reducing the exposure duration that encourages char formation. Essential factors vital for enhancing fast pyrolysis involve elevated heating rates, regulated temperatures near 500 °C, efficient char removal, and dry biomass input containing under 10 wt.% moisture. If the bio-oil has a low pH, it needs to be refined prior to use. Rapid PY is important not only for generating bio-oil but also for creating food flavors and specific chemicals [284]. According to [287], fast PY yields up to 75 wt.% bio-oil in a medium temperature range (450–600 °C), at a great heating rate (about 300 °C/min), and with a short residence time. 

**Figure 11:**
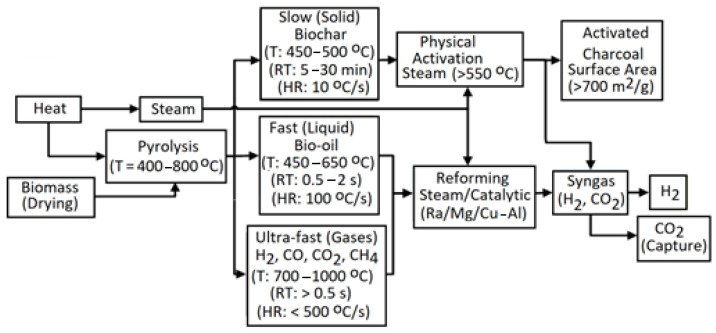
The flowchart of PF (based on [39]).

Flash PY enables the rapid conversion of solid organic materials into liquid or gas products. Functioning at temperatures between 300 and 1400 °C and with a swift heating rate of 1000–21,000 °C/s, biomass particles undergo a short heat pulse lasting only 0.015 to 2 s. This short exposure triggers the swift breakdown of organic macromolecules like cellulose and lignin, resulting in the release of volatile compounds that are swiftly removed from the high-temperature area and rapidly cooled to avert additional secondary reactions. The residence time for solid particles (ranging from 0.05 to 2 mm in size) is between 0.016 and 0.34 min. Difficulties in scaling flash pyrolysis involve reactor design to support extremely short residence times at high heating rates, with issues focusing on the stability and quality of bio-oil resulting from the catalytic impacts of char and ash residues. Even with these obstacles, flash pyrolysis shows potential for effective energy conversion, though it requires sophisticated technologies for refining products and eliminating pollutants, especially to address the corrosive and unstable characteristics of the resulting bio-oil [284]. A residence period (below 1 s) shorter than that of fast PY can maximize the gas output [288]. Figure 11 shows a flowchart of H_2_ production via biomass PY (based on [39]).

Following PY, a bio-oil resembling tar is formed by condensing oxygenated molecules (ketones, phenolic compounds, aldehydes, alcohols, and carboxylic acids), along with resulting H_2_O and ash. Only H_2_O-soluble fractions of bio-oils are suitable for H_2_ production, and a steam GA unit can be incorporated to enhance their yields. The generation of H_2_ via biomass PY is greatly influenced by the type of catalyst, process temperature, type of feedstock, and residence time [289].

The yield of H_2_ production is especially enhanced by an increase in pyrolysis temperature and is further boosted by a higher heating rate and extended residence time; at elevated temperatures, fast pyrolysis yields more H_2_ compared to slow PY [281]. Lignin is the most stable component that breaks down at elevated temperatures compared to cellulose, which decomposes between 310 and 400 °C, while hemicellulose (primarily xylan) decomposes in the 220 to 350 °C range [290]. In PY, studies on pine, cottonwood, and rice straw showed that initial H_2_ emissions began at 400 °C across all species, peaking between 650 and 750 °C. However, due to variations in biomass composition, rice straw exhibited a higher release rate than pine, while cottonwood had the lowest rate [290].

Solar pyrolysis can achieve extremely high temperatures reaching 2000 °C, which has been shown to enhance gas yield [291]. Additionally, research on hot radiofrequency plasma pyrolysis was able to explore a temperature range of 900–2000 °C and achieved a high productivity of syngas [291]. The complete breakdown of unstable compounds can be observed at pyrolysis temperatures exceeding 700 °C, leading to the formation of phenolic compounds [292].

Prasertcharoensuk et al. [293] noted that increasing the PY temperature from 600 to 700 °C resulted in minimal yield variations, but they did find a 23% increase in gas yield when the temperature reached 800 °C. This pattern was due to the full release of volatiles beyond the 700 °C range and their subsequent cracking and dry reforming at elevated temperatures. They typically noted an increasing trend in CO and the H_2_/CO ratio, while CO_2_ content exhibited a downward trend, which is explained by the ongoing utilization of the emitted CO_2_ in the Boudouard reaction occurring above 700 °C and the dry reforming of light hydrocarbons driven at temperatures over 640 °C, generating additional H_2_ and CO [293]. The content of H_2_ and CO increased from 48.8 to 67.2 mol% and 4.5 to 8.8 mol%, respectively, as the PY temperature rose from 600 to 900 °C. In general, the gas yield grew from 77.8 to 95.8 wt.%, while the solid residues and ash decreased from 16.3 to 0.4 wt.%. At every temperature, the liquids primarily contain aromatics, phenolics, and furans, which suggests that high temperatures are not adequate for their decomposition [293].

During the slow PY of sugarcane by Al Arni et al., the yield of syngas improved, with H_2_ notably rising from 7 to 28.8% as the temperature was elevated from 773 to 953 K, attributed to enhanced tar-cracking reactions, leading to an increase in gases at the cost of heavier hydrocarbons. Significantly, CO and CO_2_ gases were initially prevalent due to the simpler decomposition of hemicellulose and cellulose and then subsided past 400 °C, when the gradual breakdown of lignin commenced, leading to the release of H_2_ and CH_4_ [294].

A similar observation was noted, as H_2_ content rose notably from 1.13 to 16.5 vol% when the PY temperature of palm kernel cake was raised from 500 to 700 °C and from 2.03 to 20.36 vol% for cassava pulp residue [281]. A considerable rise in syngas yield was noted, from 26 to 46%, due to a temperature increase from 300 to 700 °C during the slow PY of *Salsola collina Pall*. A similar pattern was observed for the pyrolysis of pine wood and corn stalks [295]. Cheng et al. [296] observed a reduction in bio-oil yield and an elevation in gas yield after surpassing 600 °C during the PY of Crofton weed. The content of syngas and H_2_ was enhanced by elevating the temperature from 350 to 800 °C during the PY of Alcell lignin, achieving a H_2_ content of 31.5 mol%. A similar effect was observed with coconut shells at temperatures ranging from 500 to 900 °C, where the gas yield rose notably from 36.59 to 64.47 wt.%, and H_2_ levels increased from 3.56 to 15.04 vol% at 800 °C, ultimately reaching 33.49 vol% at 900 °C. In the inline PY–catalytic SR of pyrolysates from sawdust using 10 wt.% Ni/Al_2_O_3_, both gas production and H_2_ yield improved as the temperature increased, with peak H_2_ concentration occurring between 600 and 700 °C [281].

Syngas is a dangerous material that presents fire, explosion, and toxicity risks due to the physical and chemical characteristics of its components. The progression of an emergency situation involving syngas is influenced by system operating conditions, gas composition, and/or the onset of ignition [297].

The buildup of adequate levels of syngas can present a fire or explosion hazard when there is an ignition source and enough O_2_. This can happen in confined areas like dryers, dryer cyclones, combustion chambers, ducts, or pipes. The buildup of ample quantities of syngas may present a fire or explosion hazard if an ignition source and enough O_2_ are present. This can happen in confined areas like dryers, dryer cyclones, combustion chambers, ducts, or pipes [298]. 

Dangerous areas emerge around a compromised synthesis gas pipeline. The dimensions of the zones depend on the gas composition and are typically much smaller when there is a release of syngas produced from biomass gasification. It also depends on the extent of damage to the pipeline. The generated hazard zone is largest if the pipeline is fully ruptured [299].

Syngas possesses 50% of the energy density found in NG. It cannot be ignited directly yet serves as a source of fuel. Another application is as a precursor for manufacturing additional chemicals. The creation of syngas for use as a feedstock in fuel production is achieved through the gasification of coal or urban waste. In these processes, carbon interacts with H_2_O or O_2_ to produce carbon dioxide, carbon monoxide, and hydrogen. The safety requirements for syngas involve a mix of safety procedures for H_2_ and CO, as both are found in considerable amounts [300].

Typically, the heating rate determines the PY classification and influences the output of all products, which can be particularly important for the yield of gases. With a high heating rate, the gas yield would rise at the cost of oil and char yields due to the quicker depolymerization of complex structures into primary volatile matter and vapors, which undergo secondary cracking and decomposition at such elevated heating rates. Conversely, a reduced heating rate would hinder dehydrogenation and secondary reactions, resulting in increased oil and char production. Nonetheless, certain studies indicated that a limited range of heating rates analyzed, between 5 and 20 °C/min, had a minimal impact on gas yield, whereas a higher rate exceeding 30 °C/min would significantly influence it. In a study on the pyrolysis of refuse-derived fuel (RDF), it was noted that the gas yield rose from 14 to 47% when the heating rate was elevated from 5 to 350 °C/min, while there was a significant drop in bio-oil from 55 to 23% and a more gradual reduction in solid content. This trend was explained by the breakdown and conversion of both oil and solid materials [281].

As reported by Safdari M-S et al. [301], it is not an absolute trend, as they observed that elevating the rate from 30 to 1000 °C/min at a steady temperature of 765 °C resulted in a decrease in char and light-gas yields, while the tar content increased by 49–60%.

Concerning the residence time, a brief PY duration is inadequate for decomposition and fails to generate sufficient heat for the reactions to occur, resulting in incomplete pyrolysis. Typically, an elevated operating temperature, combined with a rapid heating rate and adequate residence time, propels the process kinetics to achieve a greater gas yield [281].

In slow PY with an extended residence time, repolymerization occurs, resulting in char production, while facilitating the conversion of liquids/tar into a greater gas yield. In a different study examining the pyrolysis of palm oil waste without a catalyst, the total gas yield initially rose with longer residence times, peaking at approximately 14 s, then slightly declined with additional increases in residence time. Specifically, a rise in residence time resulted in an initial abrupt decrease in H_2_ yield, whereas further extension of the residence time resulted in an increased H_2_ yield. Nevertheless, excessive prolongation of the residence time results in decreased H_2_ output, even though extending the residence time promotes the thermal cracking of heavier hydrocarbons, which in turn boosts the gas release rate. However, this also causes some H_2_ to be consumed in reactions and results in the generation of other gaseous products like CO_x_ and CH_4_ [302].

PY–SR is a modern two-step thermochemical technique that has garnered significant interest [303]. The catalysts employed are identical to those applied in the cracking stages of GA, and they, too, become deactivated due to C buildup (coke). A catalyst regeneration stage enables the conversion of coke into CO_2_ via combustion, thereby clearing the active sites [304]. Global H_2_ production rates are comparable to those achieved via SR through GA, falling within the range of 70% to 80% [305].

Isolating the reactors in the PY–steam reforming process prevents the formation of coke deposits that could hinder the reforming catalyst [306]. The method is easy to expand and can serve as an alternative to direct GA and the reformation of bio-oil [307].

Table 6 contains details about the hazards associated with the biomass PY process.

**Table 6:** Hazards related to TC methods used for H_2_ extraction from biomass.

Methods		Information of Hazards	Refs.
PY		Efficient separation and purification of end products are necessary because of high temperatures and long residence times, leading to high energy consumption.	[308]
		Plants that transform C-based substances into energy have the following hazards: The risk of fire and explosion is high due to the highly flammable gases (H_2_ and CO) present. The large amounts of CO could lead to a potentially serious toxic gas release. Biochar created after PY is harmful in soil applications, contributes to GHG emissions, hinders the efficacy of pesticides, and impacts soil microorganisms. The risks come from feedstock, feedstock contamination, and PY conditions. The presence of these harmful substances in food endangers human health.	[309]
		Certain biomass samples are not environmentally friendly during PY.	[310]
		Biomass dominated by lignin produces products dominated by char, significantly impacting two environmental categories. Cellulose-rich biomass has an impact on six other categories by generating oil-rich products. Biomass rich in hemicellulose produces gases with high levels and minimal environmental impacts.	[311]
HT processes	HTC	Process water (PW) with a short retention time contains toxic phenols, furfurals, and their derivatives, which enable AD to produce biogas (BG).	[312]
		The PW is contaminated by both organic and inorganic sources and requires treatment. The issues concerning the management of stable and toxic organic substances such as phenols, phenolic compounds, furfural, and 5-HMF remain unresolved. These compounds can sometimes be hard to break down through biological processes (having high COD-BOD5 ratios), which could create challenges in treating PW. Mitigation of hazards: Post-PW should be directed to an appropriate sewage treatment plant.	[89]
	HTL	The reactors, which are intricate and costly, operate at high pressures and have a high capacity for H_2_O management. It is challenging to measure the product yields in the HT reaction. It creates coke and tar. The reactor becomes blocked and obstructed as a result of organic salts precipitating together.	[313]
	HTG	High levels of energy consumption and technological requirements for the process are costly.	[314]
GA		The safety of startup processes is influenced by the temperature used for heating. The possibility of fire and explosion, along with the release of environmental pollutants through various routes, is a concern due to the presence of a flammable gas mixture with a high amount of H_2_ gas produced under high-temperature and high-pressure conditions.	[315,316]
		The specific dangers include the possible release of harmful gases such as CO, SO_x_, NO_x_, and particles.	[317]
		CO can enter the bloodstream and bind with hemoglobin to inhibit O_2_ absorption and circulation. Prolonged exposure to CO can lead to asthma, inflammation of the lungs, schizophrenia, and heart defects. Harmful gases such as SO_x_, NO_x_, and volatile organics can harm human respiratory, digestive, and skin systems.	[318]
		CO is an extremely poisonous gas. It is referred to as a toxic (blood) asphyxiant, indicating that it diminishes the blood’s ability to transport O_2_. Low-level doses of CO can lead to headaches and dizziness; however, if the person is taken to fresh air, no lasting harm will occur. Elevated levels, however, can saturate an individual’s blood within minutes and rapidly result in respiratory failure or demise. The existing allowable exposure limit for CO is determined by a Time-Weighted Average (TWA) of 30 ppm. Even though extremely high levels of CO can be acutely harmful, potentially causing immediate respiratory failure or death, it is the long-term health impacts from chronic exposure at lower concentrations that have the most significant effect on affected workers. Exposure levels are insufficient to cause immediate symptoms; however, frequent small doses gradually diminish the blood’s ability to carry O_2_ to dangerously low levels. The mitigation of hazards comprises the following measures: Be aware of the signs of CO poisoning: headaches, disorientation, tiredness, convulsions, lightheadedness or fainting, and queasiness. Seek fresh air and obtain medical assistance right away if exhibiting these symptoms. Set up and regularly check a battery-powered CO detector. Arrange periodic maintenance inspections for the engine and exhaust system conducted by skilled and trained professionals. Notice that hazardous levels of CO can build up in just seconds.	[319,320]
		In elevated amounts, gaseous SO_x_ can negatively impact trees and plants by harming leaves and inhibiting growth. SO_2_ and various sulfur oxides may lead to acid rain that can damage delicate ecosystems. SO_2_ may lead to respiratory issues like bronchitis and can irritate your nose, throat, and lungs. It can lead to coughing, wheezing, mucus production, and asthma episodes. The impacts are more severe during physical activity. SO_2_ has been associated with heart disease. The mitigation of hazards comprises the following measures: Systematic control of SO_x_ in air. Use of efficient ventilation in places where people are exposed to SO_x_. Use of respiratory protection equipment in places with increased SO_x_ concentrations. Developing plans to lower the amount of SO_2_ in the air. Utilize techniques to reduce SO_x_ production, including fuel purification systems and alterations in combustion processes. Whenever feasible and economical, opt for dry SO_x_ removal systems instead of wet ones.	[321,322,323]
		Low concentrations of NO_x_ in the atmosphere can irritate your eyes, nose, throat, and lungs, potentially leading to coughing and symptoms such as shortness of breath, fatigue, and nausea. Being exposed to low levels can also lead to fluid accumulation in the lungs one or two days following the exposure. Inhaling elevated amounts of nitrogen oxides may lead to quick combustion, spasms, and inflammation of the throat and upper respiratory area, decreased O_2_ supply to body tissues, fluid accumulation in the lungs, and death. Skin or eye contact with high levels of nitrogen oxide gases or liquid nitrogen dioxide would probably result in severe burns. Possible reduction in hazards: Reducing NO_x_ and N_2_O emissions is a significant issue since these compounds can inflict considerable harm to the atmosphere, soil, H_2_O, and human health. Currently, selective catalytic reduction is the most efficient and common technology for eliminating NO_x_ from flue gases. Additional approaches are utilized selectively, particularly those below: Catalytic breakdown of NO_x_ and N_2_O, where nitrogen oxides are broken down without other reactants, can be used. Selective non-catalytic reduction is commonly employed when catalytic beds cannot be used or are restricted. Electron beam irradiation and electrochemical reduction represent some of the most promising emerging technologies.	[324,325]
		Particle emission (PM2.5) induces cancer. Their ability to soak up various soluble organic compounds such as alkanes, carboxylic acid, and aromatic compounds can harm the lungs and livers of humans. Short-term exposure to PM2.5 (lasting up to 24 h) is linked to early death, heart or lung issues, both acute and chronic bronchitis, asthma episodes, respiratory problems, and limited activity. PM2.5 particles in the air can penetrate deeply into the respiratory system, reaching the lungs and leading to immediate health issues such as irritation in the eyes, nose, throat, and lungs, along with symptoms like coughing, sneezing, a runny nose, and difficulty breathing. The mitigation of hazards comprises the following measures: Ongoing enhancement of broad application and effective advancement of clean energy. Enhancing the dissemination of air quality monitoring and related data. Enhancing understanding of the dangers of air pollution and providing suggestions for safeguarding public health against air pollution. Enhancing the health safeguards for groups at risk from ambient air pollution. Crafting sensible outdoor travel plans, accurately donning masks that filter PM2.5 while outside, promptly and suitably opening windows for indoor ventilation based on air quality, and utilizing purification devices to lower indoor PM2.5 levels during periods of severe air pollution. Enhancing studies on air pollutant detection technology and surveillance systems to advance precise exposure evaluation.	[326,327,328,329]
		Environmental issues can arise when ashes and condensates produced from biomass gasification are not appropriately disposed of. Dealing with a toxic condensate that has a high tar content is challenging and presents greater hazards.	[330]

PY comes with challenges related to separating and purifying H_2_ from complex gas mixtures, reducing GHG emissions, and the expensive and complicated process of large-scale H_2_ production [265].

The weaknesses of biomass PY involve high energy usage and the requirement for the effective separation and purification of the final products. The high energy consumption is due to the use of high temperatures and long residence times in the process [308].

Conversion facilities that turn C-containing substances, such as biomass and municipal solid waste, into a gas product and PY exhibit the following hazards:

- Potential dangers of fire and explosion caused by the existence of extremely flammable gases (H_2_ and CO).
- Dangerous release of toxic gas, with potentially severe outcomes, caused by the abundant presence of CO [309].

The characteristics of the raw materials and the resulting biochar created during various pyrolysis methods impact their chemical, physical, and structural properties. Biochar use has been associated with certain dangers when used in soil, including biochar toxicity, reducing GHG emissions, diminishing pesticide effectiveness, and impacting soil microorganisms. Possible dangers arise due to the feedstock, tainted feedstock, and pyrolysis conditions that support the formation of characteristics and functional groups of this type.

The harmful chemicals created represent a danger to human health via the food web [310].

Biomass with a high lignin content results in products dominated by char, impacting two environmental categories, while biomass rich in cellulose mostly affects six other categories while producing products dominated by oil. Samples rich in hemicellulose produce products with high gas content and gentle environmental impacts. During the PY process, not every biomass sample is environmentally friendly [311].

Ndirangu et al. [310] highlighted the importance of assessing biochar risks and examined risk evaluation concerning the PY process, feedstock, and hazard sources in biochar, along with their possible impacts and the methods employed in risk assessment.

Cordella et al. [331] performed a comprehensive experimental study to collect detailed and quantitative data regarding the composition of bio-oils produced from biomass slow PY. Comprehensive composition data were collected for the primary components and for the polycyclic aromatic hydrocarbons (PAHs) found in bio-oils from corn stalks, poplar, and switchgrass. These data were utilized to evaluate the hazard profiles of the bio-oils. Particular screening methods were created, capable of generating hazard scores for the bio-oil characteristics derived from the individual bio-oil components. Their findings indicate the following:

- Risks to human health may be linked to long-term exposure to bio-oils.
- Severe harmful impacts on humans and eco-toxic impacts on H_2_O environmental ecosystems might also occur in the event of a loss of containment.
- A slight carcinogenic risk might arise from the existence of cancer-causing substances such as catechol and PAHs. Therefore, evaluating and managing the risks associated with bio-oils is a crucial factor to consider for ensuring the safe and sustainable utilization of products derived from biomass PY.

According to [332], bio-oil remains stable in typical ambient conditions, but it can react with strong acids or powerful oxidizing agents like chlorates, nitrates, peroxides, and others. When it comes into contact with the eyes, it can result in irritation. Frequent contact with the skin can lead to irritation. When heated, its vapors might induce nausea and irritation in the eyes and upper respiratory system.

The mitigation of hazards comprises the following measures [332]:

- Remove all ignition sources near the spilled material.
- Steer clear of heat, sparks, flames, and oxidizers.
- Steer clear of exposure to mineral acid/alkali.
- Halt the origin of the release if you can accomplish it safely. Perform release containment to avoid additional pollution of soil, surface H_2_O, or ground H_2_O.
- Use protective gear to avoid eye contact and skin exposure.
- Typically, no respiratory protection is needed. Nevertheless, utilize a positive-pressure air-supplying respirator in situations where air-purifying respirators are insufficient.

According to [60], in contrast to combustion, PY and co-PY produce fewer air pollutants and serve as alternative methods for hazardous material and biomass waste disposal instead of landfills and incineration.

Biomass PY is in the developing stage, and its cost is equal to 2.8 USD/kg. The process’s drawbacks include the production of tar, CO_2_, and CO. The remedy for them is the requirement for post-combustion separation, oxyfuel separation, and CO_2_ storage. The process efficiency is in the range of 35–50% [291,333].

#### 4.2.2. Hydrothermal Processes of Biomass

**Figure 12:**
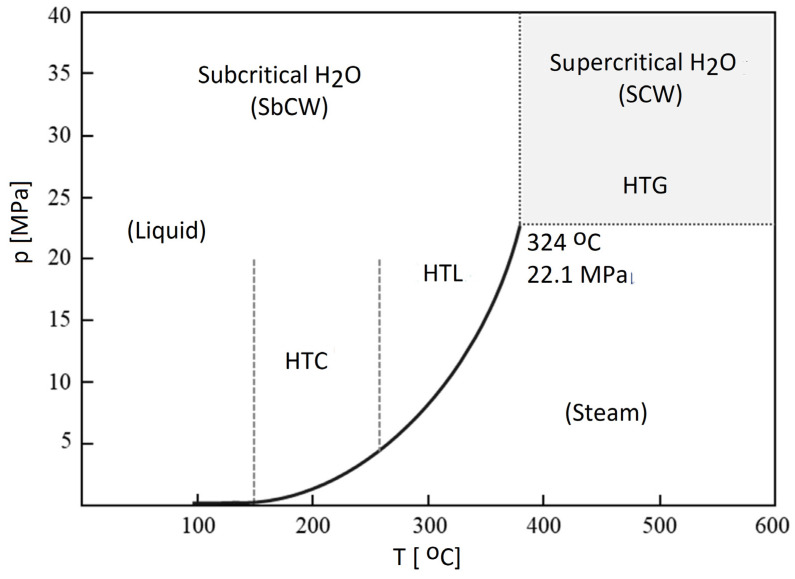
The phase diagram of H_2_O with the regions of HT processes, delineating the conditions for hydrothermal carbonization (HTC), hydrothermal liquefaction (HTL), and hydrothermal gasification (HTG). Based on [39].

HT procedures function at high temperatures and pressures that surpass the saturation pressure of H_2_O. These conditions cause various reactions that alter the physical and magnetic characteristics of H_2_O, such as its density, dielectric constant, and ionic product. Reactions like these influence the production of fuels with high energy content and valuable chemicals. Researchers classify hydrothermal (HT) processes into three main types, hydrothermal carbonization (HTC), hydrothermal liquefaction (HTL), and hydrothermal gasification (HTG), based on the temperature range and target products [334] (Figure 12 [39]).

**Figure 13:**
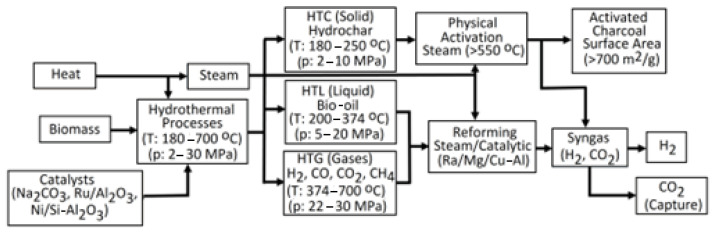
The renewable-H_2_ production process performed via biomass HT processes (based on [39]).

Figure 13 (based on [39]) shows the specific operating parameters mainly affecting the maximalization of biomass conversion into valuable gases and activated charcoal via HT processes. This figure presents the critical temperature and pressure settings needed to maximize efficacy and H_2_ yield in the biomass conversion process.

Producing hydrochar via biomass HTC is an effective and environmentally friendly process, creating a C-rich solid fuel that has a high energy density. This process converts various biomass materials into clean solid fuels without producing smoke, working at pressures between 2 and 10 MPa and temperatures between 180 and 250 °C [335]. Hydrochar can undergo additional processing by being physically activated with steam at high temperatures (500–900 °C) to produce activated carbon, resulting in surface areas exceeding 700 m^2^/g [336].

The HTL transforms biomass into biocrude, similar to pyrolysis bio-oil, via chemical processes at high temperatures (280–370 °C) and pressures (10–25 MPa). This process also produces solid, liquid, and gas byproducts with higher energy content and better heat recovery capabilities compared to other methods [337]. Additional treatment of biocrude using reforming methods can convert it into bio-CH_4_ and H_2_ [338].

HTG, or supercritical H_2_O gasification (SWG), makes use of H_2_O in its supercritical state as both a solvent and a reactant due to its unique properties. This process converts biomass into H_2_ and CO_2_ using catalytic cracking and steam under specific conditions (T ≥ 374 °C, P ≥ 22 MPa) [339]. In these circumstances, H_2_O acts as an oxidizing agent and engages in a steam reforming (SR) process with biomass, following Formula (11), to produce H_2_, CH_4_, CO, and CO_2_.

(11) $$Biomass+H_{2}O\to H_{2}+CO+CO_{2}+CH_{4}$$

After this initial reaction, similar gas–gas reactions occur, leading to the creation of H_2_ and CO_2_, as outlined in Equations (12)–(14) [339].

(12) $$CH_{4}+CO_{2}\to {2H}_{2}+2CO,$$

(13) $$CH_{4}+H_{2}O\to {3H}_{2}+CO,$$

(14) $$CO+H_{2}O\to CO_{2}+H_{2}$$

HTG and the other HT processes utilize H_2_O as both the reaction and reagent medium, without the necessity of drying the feedstock before the process, thus lowering energy consumption [340]. This method is beneficial for the drying of biomass with high levels of moisture. It also enables the attainment of high biomass conversion rates (up to 100%) and a volumetric H_2_ content of at least 50% in final gaseous products while producing less tar or other byproducts [341]. Optimizing the operating temperature and pressure, reagent concentration, and reaction time is necessary to achieve high yields. 

Some information regarding hazards accompanying diverse HT processes of biomass is presented in Table 6.

In HTC, biomass is treated with H_2_O at low temperatures (around 200 °C) and pressures for several hours [342]. This method consumes less energy compared to traditional carbonization and enables a greater product yield in a shorter period [343]. Its benefits encompass a low carbonization temperature, synthesis in the aqueous phase, and the utilization of biomass [343].

The primary benefit of HTC compared to PY is that the HTC process handles wet waste, enabling feedstocks to be processed without the need for pre-drying. Numerous feedstocks, such as aquatic biomass, agricultural byproducts, and industrial as well as animal wastes, are appropriate for HTC [344]. PW serves as an efficient medium for heat transfer in HTC; however, if the variation in feedstock particle size is excessive and the reaction time is insufficient, it can lead to mass transfer limitations. Therefore, the particle size must be consistent to guarantee even heat and mass transfer. The feed material introduced into the reactor is warmed to a designated temperature and maintained for a specified duration. During HTC, gases (mainly CO_2_) and a liquid mixture (predominantly PW with a small number of organics and solids) are generated. The liquid mixture is centrifuged or filtered to divide the PW from the solids (wet cake). The moist cake is subsequently dried to generate a carbon-dense hydrochar [345].

Various reactions take place during the HTC process, including hydrolysis, dehydration, decarboxylation, and aromatization. These reactions take place at elevated temperatures and pressures and are crucial for reducing the hydrogen to carbon (H/C) and O_2_ to carbon (O/C) ratios to yield carbon-dense hydrochar. Hydrochar is the main product due to its characteristics allowing its use as a solid fuel [345].

The hydrochar yield depends on the type of feedstock, the solids loading (the ratio of feedstock to H_2_O), as well as the process temperature and residence time. Overall, hydrochar production diminishes as process conditions become more severe, meaning that elevated temperatures and extended residence times break down a greater portion of the cellulosic and hemicellulosic components in the feedstock. Although the yield is low, the hydrochar exhibits a greater C content and higher heating value (HHV) when subjected to elevated temperatures and extended residence times [346].

The HTC hydrochar contains less moisture and is more hydrophobic compared to the raw feedstock [347]. Nevertheless, obtaining these characteristics necessitates the use of reactors and machinery, like a filter press, resulting in an energy-intensive procedure. HTC hydrochar and HTC PW can contain unwanted metals like Ni, Pb, Cd, and Cr, depending on the feedstock utilized, which are found in both solid and liquid fractions after the HTC process [345].

Depending on the feedstock, hydrochar possesses a calorific value ranging from 15 to 30 MJ/kg, which is marginally greater than the usual raw HTC feedstocks, which vary from 13 to 19 MJ/kg [345]. However, certain hydrochar possesses enough energy content to serve as solid fuel. Furthermore, hydrochar can be used as a feedstock for the production of liquid fuels (bio-oil, blend-stock fuel) and gaseous fuels (syngas) [348,349].

HTC does not necessitate the pre-drying of biomass and can use feedstocks with different moisture levels, resulting in savings on energy and drying costs prior to processing [350]. One of the primary advantages of HTC over other thermochemical processing techniques is that it does not need dry feedstocks to create char [351]. HTC increases the dewatering efficiency of raw materials by aiding in the release of bound H_2_O, making it extremely advantageous for biosolid management [352]. HTC can significantly reduce the environmental effects of waste biomass since it generates more energy and releases far fewer pollutants and odors compared to incineration, landfilling, and composting [353]. HTC can handle biomass without the need for pre-drying and is capable of using feedstocks with different moisture levels, resulting in energy and cost savings on drying prior to processing [350]. This is a significant advantage of HTC over other thermochemical processing techniques that need dry feedstocks to create char [351]. HTC can reduce the environmental effects of waste biomass by recovering greater energy and producing significantly fewer pollutants and odors compared to incineration, landfilling, and composting [353].

Yang et al. [354] explored the properties of the aqueous phases (APs) obtained from various agricultural residues (such as peanut and wheat straws) and the co-gasification of cotton stalk (CS) along with APs. Hydrothermal temperatures significantly affected the development of primary gaseous products. By experimenting and analyzing the co-gasification of CS and APs, it was revealed that the syngas product from co-gasification was primarily hydrogen. The hydrothermal temperature of the AP affected the spread and output of syngas products. In all experiments, the highest hydrogen content reached 57% (maximum syngas output, 1657 mL).

According to [345], the capital expenditure for an HTC system differs significantly based on the reactor size and operational scale. Operating expenses encompass elements like feedstock costs, HTC conditions (temperature and duration), and the characteristics of the intended product. Few studies exist regarding the costs associated with hydrochar production since the technology is not yet broadly commercialized. Nevertheless, according to the existing literature, the expenses of hydrochar generated from various feedstocks, including a coal–miscanthus mixture, compost, and grape marc, range from USD 106 to USD 170 per ton. The primary factors influencing the expense of hydrochar production include the size of the production facility, the characteristics of the feedstock, and the yield of hydrochar. There are still several uncertainties linked to heat transfer dynamics, product yields, and the expenses of large-scale hydrochar production. Broader research on various feedstock combinations, large reactors, and effective hydrochar production techniques may enhance the process and decrease resource needs and expenses.

Hydrothermal carbonization (HTC) is a TC pretreatment process in which biomass is treated with hot pressurized H_2_O to produce hydrochar. The latter is a persistent, hydrophobic, friable solid product, which has a fuel value close to that of lignite coal. The HTC process realized with a short retention time is accompanied by PW comprising potentially toxic phenols, furfurals, and their derivatives. This allows AD to generate BG [312].

If untreated, the process H_2_O (PW) from hydrothermal carbonization (HTC) would cause significant environmental contamination due to both organic and inorganic pollutants. During HTC, the creation of stable and harmful phenols, phenolic compounds, furfural, and 5-HMF causes issues because they can leach into the PW. Sometimes, these compounds are not easily broken down by biological processes (having high COD-BOD5 ratios) and can make the treatment of PW more complicated. The PW makeup is primarily influenced by the waste biomass used initially and the reaction conditions [355].

Usman et al. [356] noted in their review that HTL’s adaptability in transforming various biomass or waste products into biocrude is significant, allowing high biocrude yields (reaching 60–86% from assorted biomass types). Researchers investigated the impact of catalysts on HTL processes, the scalability of the technology, and the prospects for commercializing continuous HTL with aqueous-phase recycling. They additionally investigated the application of machine learning integration for HTL optimization aimed at improving process efficiency and the quality of output. They identified HTL and the following hydrotreatment as essential methods for converting biomass into a renewable fuel source.

The selection of the catalyst is vital in the HTL process, influencing biocrude production, its quality, and the efficiency of biomass transformation. Although alkaline catalysts, alkali salts, and metal-based catalysts typically demonstrate potential in improving biocrude production for certain feedstocks, their effects may differ based on the type of biomass. Conversely, acidic environments generally encourage repolymerization and condensation processes, leading to lower biocrude production. These insights highlight the importance of customized catalyst selection to enhance the advantages of HTL for various feedstock sources [356].

Research on continuous-flow HTL systems can be conducted using both lab-scale continuous-flow reactors and batch reactors with rapid heating. Nonetheless, creating a lab-scale continuous-flow system poses multiple challenges due to its complexity, safety factors, and the constraints of reducing its size, such as obtaining a lab-scale high-pressure pump [357]. Consequently, most studies on continuous-flow HTL systems utilize reactors that lie between laboratory and pilot scales, providing a feasible balance.

Continuous-flow HTL systems generally demonstrate reduced reaction times and greater biocrude outputs compared to results from commonly utilized batch HTL reactors. Importantly, even with considerable industry funding and various initiatives, there is still a notable lack of information on performance failures and issues faced during the design, construction, startup, and operational stages in the industrial setting concerning continuous-flow HTL systems [356].

The recycling of the H_2_O phase in HTL is seen as a hopeful method to boost biocrude production and increase energy recovery. Multiple studies mentioned earlier have shown substantial advantages, such as higher biocrude production and improved resource efficiency. Nevertheless, it is essential to recognize the related difficulties, including heavy-metal buildup, changes in biocrude characteristics, and concerns regarding nitrogen content. Tackling these issues will be vital for the effective execution of aqueous-phase recycling in HTL processes [356].

In their research on the TEA and LCA of HTL involving microalgae and the later hydrotreating of the generated biocrude, Masoumi and Dalai [358] indicated a minimum selling price (MSP) of USD 2.2 per liter to achieve an operational breakeven. Their LCA analysis showed a significant performance in GHG emissions, with an estimated decrease of −1.13 gCO_2_-eq per MJ, markedly less than that of traditional petroleum-based fuel production. A different analysis of TEA and LCA regarding HTL camel manure biocrude indicated that the upgraded biofuel had an MSP ranging from USD 0.87 to USD 0.91 per kg. Moreover, LCA showed a 7% decrease in GHG emissions when evaluating the biofuel generated from camel dung (84 gCO_2_/MJ) against commercial gasoline (90.2 gCO_2_/MJ), highlighting its ecological benefits [359].

A research project carried out in 2019 by de Rose et al. [360] evaluated the HTL of microalgae, revealing an MSF of USD 12.85/GGE for the biochemical pathway and USD 10.41/GGE for the thermal–chemical pathway. Their LCA showed a notably reduced global warming potential for the thermal–chemical method at 2 g CO_2_eq/MJ, in contrast to 111.2 g CO_2_eq/MJ for the biochemical method. The aforementioned studies suggest that the HTL of biomass is not only economically feasible but also environmentally advantageous, especially regarding GHG emissions. Nevertheless, it is crucial to recognize that the results of TEA and LCA can differ greatly based on several elements, like the kind of feedstock, its makeup, and operational parameters [361].

HTL presents several challenges [356]:

- A major challenge is the existence of heteroatoms, like sulfur and nitrogen, in HTL biocrudes. Effectively controlling these heteroatoms via desulfurization and denitrogenation processes is needed. Specifically, it is essential to create strong and economical techniques to lower the sulfur and nitrogen levels in biocrudes.
- Despite numerous HTL studies being performed at laboratory and pilot levels, moving to full-scale industrial operations is complicated. Difficulties involve creating sizable reactors, guaranteeing a steady supply of feedstock, and tackling financial factors. Research needs to concentrate on expanding HTL and hydrotreatment techniques for commercial use.
- While often challenging, securing a steady supply of sustainable biomass feedstocks is crucial. The sustained availability and continuity of feedstocks are essential for commercial viability and process consistency.
- Although different catalysts have demonstrated potential in enhancing biocrude yield and quality, obstacles persist in creating highly efficient, reusable, and feedstock-flexible catalysts. Catalyst innovation represents a crucial area of research focus.
- Enhancing the hydrotreatment process is essential for attaining optimal biocrude upgrading efficiency. Additional studies are necessary to enhance selectivity, yield, and energy usage in the hydrotreatment process.

Four hazards accompany HTL: reactors are costly and intricate, requiring high pressures and H_2_O-handling capacity; product yield measurement is challenging; coke and tar can form; and organic salts combined with coke can clog and block reactors [313].

HTL is in the developing stage and costs 2.24 USD/kg. The process drawback is the low efficiency in the range of 30–35%. The remedy for this is the requirement for the Fischer–Tropsch reactor path [362,363].

HTG has disadvantages like high energy consumption and technological demand for equipment, leading to substantial investment and maintenance expenses [198].

Yoshida et al. [364] explored the SCWG processes of cellulose, hemicellulose, and lignin. They discovered that the lignin content greatly influences the quantity and composition of the product gas. This is likely because cellulose or xylan acts as a hydrogen donor to lignin. The quantity and makeup of the product gas can be reliably forecasted using solely the lignin component as a variable. This validated the significance of the lignin fraction’s influence on the SCWG features.

Bircan et al. [365] indicated that producing H_2_ via HTG can occur without taking into account the toxicity of dioxins. Dioxins were identified, yet their levels were significantly lower than the environmental regulation standards. An additional advantage discovered was that this method addresses the issue of chicken manure disposal during H_2_ generation.

Antal et al. [366] documented the HTG of corn- and potato-starch gels, wood sawdust immersed in a cornstarch gel, and potato waste delivered to three distinct tubular flow reactors via a “cement” pump. When quickly heated to temperatures exceeding 650 °C at pressures greater than the critical pressure of H_2_O (22 MPa), the organic components of these feedstocks turned into vapors. A dense C layer in the reactor facilitated the GA of these organic vapors in the H_2_O; thus, the reactor’s H_2_O output was purified. The generated gas composition was significantly affected by the reactor’s peak temperature and the state of the reactor wall. Remarkable gas yields (>2 L/g) with a high H_2_ content (57 mol %) were achieved at the peak temperatures used. Regardless of the reactor design and heating technique, all three reactors became clogged after 1−2 h of operation with feedstocks that had 15 wt.% organic content. Reduced loadings of organics extended the duration prior to plugging happening. The plug was created from buildups of ash and minor quantities of char produced by coking reactions involving the vapors of biomass. A technique for extracting plugs from the reactor was created and applied during an 8 h GA operation using potato waste. Significant erosion of the inner wall of each reactor took place during these tests. Ni and various metals were extracted from the reactor and accumulated in the C catalyst. Ni alloy tubes are inappropriate for use in this application.

Deniz et al. [367] examined the HTG of *Posidonia oceanica* in a batch reactor without the use of any catalysts. The experiments took place within a temperature range of 300–600 °C, using varying biomass loading levels of 0.04–0.12 (g/mL) over a reaction duration of 1 h. The gas produced consisted of H_2_, CH_4_, CO_2_, CO, and a slight quantity of C_2_–C_4_ compounds. They discovered that raising the temperature and lowering the biomass loading improved the HTG yield and H_2_ production by accelerating the water–gas shift (WGS) and SR reactions. The highest molar fraction and H_2_ yield achieved were 62.51% and 10.37 mol/kg, respectively, at 600 °C with a biomass loading of 0.08 g/mL.

In contrast to PY, thermal GA, and PO processes, needing substantial energy, HTG is energy-efficient without needing extra resources [368]. Moreover, the dewatering and drying of biomass is not needed [351,352,353]. For instance, the GA of biomass with a minimum of 30% moisture demands less energy than drying because it does not involve the energy needed for the phase transition from liquid to vapor [369]. Moreover, HTG facilitates integrated energy recovery since the hot effluent stream from the reactor can be utilized to preheat the feed stream at ambient temperature [370]. In contrast, traditional biomass GA methods with drying operate at lower temperatures, which complicates energy recovery from the process streams. For HTG, energy efficiencies of the process are usually noted to be between 45% and 70% [371,372]. Ro et al. [373] determined that the HTG of biomass yields a net positive energy result when the feedstock contains more than 2% solids and effective heat recovery systems are implemented. In research examining the SCWG of corn starch at 745 °C and 280 bar, an overall process energy efficiency of 76% was noted [374]. In another related study on the HTG of chicken manure, a process energy efficiency of 70% was achieved with a biomass loading of 15%. The most efficient method for enhancing energy efficiency was increasing the biomass load. Nonetheless, the overall gas yields from SCWG may decrease with increased biomass loadings, highlighting the need for thorough process optimization [375].

HTG enhances gasification efficiency, boosts the gas yield, and lowers char/tar formation while achieving high residue yields [376]. Additionally, supercritical water (SCW) uses are viewed as “eco-friendly” substitutes for corrosive acids and organic solvents [377].

SCW serves as a reactant that facilitates hydrolysis and WGS reactions, exhibiting high diffusivity and density, making it an excellent solvent for generating industrially significant gases. Nonetheless, it has not been implemented on an industrial scale due to various drawbacks, including high pressure and temperature demands, clogging, corrosion issues, and material expenses [378].

Significant amounts of gases (mainly H_2_, CO_2_, CO, and CH_4_) produced during HTG have been shown in numerous lignocellulosic materials, including rice straw, rice husk, wheat straw, peanut husk, corn stalk, corn cob, sorghum stalk, and wood sawdust [22]. Temperature, biomass loading (or biomass concentration), and reaction duration are the key factors influencing gas production. A comparison of two methods for the HTG of biomass—low-temperature (374–460 °C) catalytic GA [379] and GA without a catalyst [380]—reveals that catalytic GA faces numerous issues, including reactor corrosion due to homogeneous catalysts and the recovery of the catalysts. Thus, it is crucial to create a GA process that produces a high H_2_ yield without the use of additional catalysts. 

HTG is an alternative to traditional steam GA methods, in which steam is employed as a gasifying agent across various reactor configurations (fixed bed, fluidized bed, entrained bed, vertical shaft, etc.). The major benefit of steam GA is its ability to deliver a higher heating rate (up to 1000 °C) with a shorter residence time (in minutes) compared to HTG. Nevertheless, the primary disadvantage of steam is its significant endothermic characteristics and substantial energy input needs. In an earlier report, legume straw produced 50.6% H_2_ and 21.2% CO at 850 °C, while pine sawdust yielded 44.0% H_2_ and 28.2% CO through steam gasification in a free-fall reactor [381].

Furthermore, H_2_ gas yields of 37% and 40% were achieved from pine sawdust in a conical spouted bed reactor [382] and from olivine-supported biomass [383] at 900 °C, respectively. The greater gas yields observed in their results were due to elevated operating temperatures. It was stated that at higher temperatures above 750 °C in the biomass GA process, the gas composition was mainly affected by the WGS reaction [384].

HTG represents a hopeful technology for H_2_ generation, especially given its capacity to directly gasify biomass that has a high moisture level. This approach demonstrates great reaction efficiency and results in significant H_2_ generation [385]. Nonetheless, HTG disadvantages include that its scalability is restricted, and no known large-scale commercial system utilizing this technology exists at present. All HT technologies require ongoing research and development to enhance their effectiveness for large-scale H_2_ generation.

#### 4.2.3. Biomass Gasification

In GA, a biomass substrate is converted into a flammable gas at temperatures between 700 and 1200 °C, without combustion. This procedure requires the application of a GA agent at a restricted level, such as air, O_2_, steam (Table 7—based on [67]), CO_2_, or their combinations.

GA’s primary output is syngas, a fuel in gas form composed mainly of H_2_, CO, CO_2_, N_2_, tars, char, ash, and particles. As a result, syngas produces a combination of flammable CO, H_2_, and C_n_H_m_ compounds [386]. General Formula (15) defines the biomass stream GA.

(15) $$Biomass+Steam\to H_{2}+CO+CO_{2}+CH_{4}+LHC+Tar+Char$$

**Figure 14:**
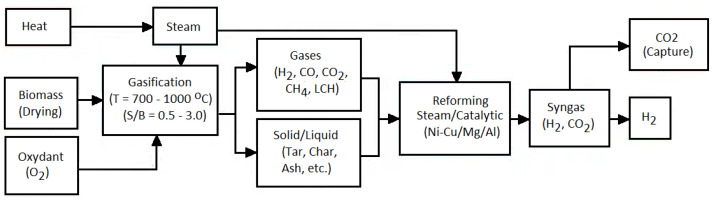
The flowchart of GA (based on [39]).

The type of biomass and the GA agent utilized can impact both the content and lower heating value (LHV) of syngas. When air is used as the gasifying agent, the resulting syngas usually has a lower heating value (LHV) between 4 and 8 MJ/Nm^3^, whereas if steam, O_2_, or a combination of both are used, the syngas produced has an LHV ranging from 8 to 20 MJ/Nm^3^ [387]. Figure 14 shows the flowchart of PF (based on [39]).

GA operations are categorized into different groups based on the type of gasifier and source of heat. Direct and self-sustaining GA involves using air or O_2_ for the partial combustion of biomass to generate process heat, while indirect and non-self-sustaining gasification uses steam along with external heat sources. Indirect and allothermal gasification is advantageous for the production of H_2_ due to its support of the SR process in syngas. This kind of response raises the production of H_2_ when compared to the gas yield from air, which primarily encourages burning and results in a larger amount of N_2_ in the syngas [388]. The SR process in GA decreases the carbon-to-hydrogen (C/H) ratio in the syngas, improving its quality by reducing the number of light hydrocarbons and tar. This improvement boosts the production of H_2_, reduces pipe clogging, and prevents corrosion caused by tar polymerization and condensation [282].

During steam GA, multiple extra reactions take place. Following the drying process, the biomass goes through a pyrolysis reaction that changes it into a gas containing CO, CO_2_, CH_4_, light hydrocarbons like C_2_H_4_, char, and tar [389], as shown in Formula (16).

(16) $$Biomass\to H_{2}O+CO+CO_{2}+CH_{4}+LHC+Tar+Char$$

Tar consists of a combination of polycyclic aromatic hydrocarbons (PAHs) and oxygenated compounds, such as alcohols, phenols, and furans, creating a complex and concentrated substance. Under high-temperature conditions (700–1000 °C) with steam, reactions such as cracking and SR occur, altering the structures of oxygenated molecules. In reforming reactions, tar decomposes into CO, H_2_, char, and polycyclic aromatic hydrocarbons (PAH) according to Formula (17) [390]. PAHs undergo thermal cracking at extremely high temperatures (~1250 °C) to generate CO and H_2_ as part of heterogeneous reactions.

(17) $$Tar+H_{2}O\to CO+CO_{2}+H_{2}+Char$$

The reforming reactions develop under a mixture of steam and CO_2_, transforming CH_4_ and C_2_H_4_ into CO and H_2_, as described by Formulas (18)–(22) [39].

Partial Oxidation of Methane (POM):

(18) $$2{CH}_{4}+O_{2}\to 2CO+2H_{2},$$

Dry Reforming of Methane (DRM):

(19) $${CH}_{4}+{CO}_{2}\to 2CO+2H_{2},$$

Dry Reforming of Ethylene (DRE):

(20) $$C_{2}H_{4}+2{CO}_{2}\to 4CO+2H_{2},$$

Steam Reforming of Methane (SRM)

(21) $$CH_{4}+H_{2}O\to CO+3H_{2},$$

Steam Reforming of Ethylene (SRE)

(22) $$C_{2}H_{4}+2H_{2}O\to 2CO+4H_{2},$$

Light HCs and CO_x_, as well as H_2_, are generated during tar cracking. These compounds remain stable during cracking/reforming conditions. Additionally, the WGS reaction changes CO when H_2_O is present, resulting in a combination of CO_2_ and H_2_, according to Formula (23) [39].

Water–Gas Shift Reaction (WGSR):

(23) $$CO+H_{2}O\to {CO}_{2}+H_{2},$$

Ellofy et al. [67] found that the biomass GA process entails a complex series of chemical reactions happening simultaneously and consecutively. Certain reactions are exothermic, releasing heat as they occur, while numerous reactions are endothermic, requiring heat to take place. The reactions may be classified as either homogeneous or heterogeneous reactions.

The introduction of steam into the GA process alters a reversible reaction to produce H_2_. Increasing the temperature of the WGS reaction, an exothermic process, favors the formation of CO and H_2_O by shifting the equilibrium toward the reactants, as stated by Le Chatelier’s principle. Finally, there are also various heterogeneous reactions observed, as defined by Equations (24)–(27), such as the oxidation of C and the Boudouard reaction, which involves the conversion of C generated during PY into CO and CO_2_ [282].

(24) $$C+O_{2}\to {CO}_{2},$$

(25) $$2C+O_{2}\to 2CO,$$

(26) $$C+{CO}_{2}\to 2CO,$$

(27) $$C+H_{2}O\to CO+H_{2}$$

Optimizing the biomass characteristics, operating temperature, steam–biomass ratio (S/B), and catalysts is crucial for achieving a high H_2_ generation yield via steam GA [283]. The effects of such parameters on the H_2_ generation yield in steam GA are shown in Table 8.

Table 6 shows details about the hazards associated with biomass GA.

**Table 8:** Effects of the main parameters on H_2_ yield in biomass steam GA.

Parameters	Effect on H_2_ Yield in Biomass Steam GA	Refs.
Temperature	Increasing the temperature encourages endothermic processes like hydrocarbon reforming (Formula (17)), methane reforming (Formula (21)), and carbon gasification (Formula (23)), leading to enhanced gas production and a higher volumetric fraction of H_2_ in syngas.	[391],
	Raising the temperature further reduces the tar content in syngas.	[392]
	Temperatures exceeding 950 °C can inhibit the WGS reaction (Formula (23)), an exothermic process, resulting in a reduction in the H_2_ content in syngas.	[393,394]
Steam to biomass ratio (S/B)	The S/B ratio plays a crucial role in determining the syngas composition and energy input in biomass gasification. A low S/B ratio results in the creation of solid C and CH_4_. Augmenting steam availability enhances the reforming processes of C and CH_4_ into CO and H_2_. An S/B ratio exceeding 1.3 leads to excess steam, which decreases solid C and CH_4_ while increasing H_2_ and CO_2_ production. Steam aids in lowering CO through the WGS (Formula (23)) and hydrocarbon reforming reactions (Formulas (17), (21), and (22)). Ideal S/B ratios of up to 1.3 promote H_2_ production, whereas ratios above 1.3 lower temperature and elevate tar creation.	[282,314,395]
Biomass characteristics	Biomass type: A composition high in cellulose and lignin increases the yield of gaseous products and the H_2_ percentage in syngas.	[314,396]
	Particle size: Reduced particle sizes enhance the surface area, facilitating better heat transfer, increasing gasification rates, and elevating H_2_ content while minimizing tar production. Particles less than 1 mm may raise energy usage.	[283],
	Moisture content: The ideal range is 10–15% by weight; levels over 40% result in lower temperatures, increased energy consumption, and decreased GA efficacy and H_2_ levels.	[395,397]
	Ash content: Elevated ash levels boost coke and particulate generation in syngas, requiring efficient gas-cleaning methods.	[396,397]
Catalysts	Boost H_2_ and CO yields by optimizing heat and mass transfer efficiency during the GA process (Formulas (23)–(27)).	[306]
	Catalysts additionally assist in tar elimination, enhancing H_2_ yield and overall GA efficacy, thereby augmenting renewable-H_2_ generation.	[282]
	Typical catalysts consist of Ni-based compounds, alkali metals (such as K_2_CO_3_, Na_2_CO_3_), alumina, aluminosilicate, ZnCl_2_, and dolomite.	[283]

The initiation of the heating process is crucial for the safety and stability of biomass GA, as it can impact the chances of fire, explosion, and the release of toxic gases. The startup process’s crucial factor is the heating temperature because it influences safety. The biomass GA process poses significant hazards, such as fire, explosion, and environmental emissions through numerous pathways [315,316].

One of the primary dangers is the possible release of harmful producer gas and particles. The incomplete combustion and oxidation of trace elements in feedstock are involved in the production of CO, SO_x_, NO_x_, and volatile organics [317]. CO, one of the most hazardous elements, can enter the bloodstream and bond with hemoglobin, preventing the absorption and distribution of O_2_. Prolonged CO exposure can result in asthma, lung inflammation, schizophrenia, and cardiac defects. Harmful gases such as SO_x_, NO_x_, and volatile organic compounds can also damage the respiratory, digestive, and skin systems of humans [318]. Particle emission (PM2.5) causes cancer. PM2.5 particles have the ability to absorb various soluble organic compounds like alkanes, carboxylic acid, and aromatic compounds, leading to harm to organs such as the lungs and liver [326]. If ashes and condensates from biomass GA are not disposed of correctly, they also add to environmental issues. Specifically, dealing with a toxic condensate containing high levels of tar is challenging and increases the likelihood of danger.

GA also poses a danger of fire and explosion. Since the GA system typically functions under high-temperature and high-pressure conditions, it also generates a flammable gas mixture containing a significant amount of H_2_ gas [330].

HT GA processes of biomass can be categorized into three main types based on the reaction conditions and desired main product gases [398]:

I. A process called aqueous-phase reforming (APR) involves converting biomass-derived compounds (C_6_H_12_O_6_, C_6_H_14_O_6_, C_3_H_8_O_3_, CH_3_OH, and C_2_H_6_O_2_) into mainly H_2_ and CO_2_ at temperatures between 215 and 265 °C with the help of a catalyst (initially Pt, later also Ni, Ru, Rh, Pd, and Ir).
II. The catalyzed near-critical GA of biomass or organic compounds at approximately 350 °C in the liquid phase or 400 °C in the supercritical state using a heterogenous catalyst converts them mainly to CH_4_ and CO_2_ for combustion.
III. Supercritical H_2_O gasification (SCWG) involves the gasification of biomass or organic compounds to produce mainly H_2_ as a burnable gas and CO_2_, without requiring the use of a solid catalyst or the presence of C or other solid catalysts.

Aqueous-phase reforming (APR) mainly converts O_2_-rich compounds into H_2_. In the aqueous phase, feedstock molecules dissolve and interact with H_2_O molecules at low temperatures (270 °C) and elevated pressures (up to 50 bar) [202,399]. APR techniques are suitable for biomass-based oxygenated hydrocarbons with a C/O ratio of 1:1 that can mix with H_2_O, including CH_4_O, C_2_H_6_O, C_2_H_6_O_2_, C_3_H_8_O_3_, glucose, or polyols such as sorbitol [400].

The primary drawback is the restricted range of efficiently soluble compounds altered by the process. Nevertheless, after a pretreatment phase, APR has the potential to transform pure cellulose and woody biomass [399]. This involves hydrolyzing cellulose and hemicellulose to generate monomers that can be used as feedstocks [401]. The reaction mechanism resembles that of SR and relies on the breaking of C–C bonds to generate H_2_. When ethylene glycol interacts with a catalyst’s surface, the C–C bonds break apart, producing CO. The catalyst further improves the WGS process, resulting in only 300 ppm of CO in the gas stream. Other reaction intermediates exist, promoting C–O bond breaking and leading to the formation of alkanes (CH_4_, C_2_H_6_) and a lower yield of H_2_ production [402].

The selection of a metal (M)-based catalyst is significantly affected by the stability of the M–C bond (which enhances the C–C cleavage route) and the M–O bond (which enhances the C–O cleavage pathway). Conversely, the expense of metal catalysts is prohibitively high for extensive development. In addition, the role of catalysts should be taken into account. Due to their greater selectivity for H_2_ and lower selectivity for alkanes, basic and neutral supports are preferred over overly acidic ones [67]. In acidic conditions, alkanes are generated via dehydrogenation, making it essential to choose a catalyst that does not selectively favor alkanes while functioning at an appropriate pH [202]. The selectivity of APR is also affected by the biomass feedstock and the parameters of the process [402,403]. Transforming intricate O_2_-containing compounds, like carbohydrates, is challenging due to their lower H_2_ output [404]. The uniform thermal breakdown of carbohydrates generates a significant amount of coke that hinders the catalyst and interferes with the reforming processes [67].

Ellofy et al. [67] found that the SR strategy represents a valuable supplementary technology that can be integrated with existing GA operations to reduce tar and char levels. To improve H_2_ production, the syngas discharged from the gasifier must exhibit a high concentration of H_2_ and a minimal tar output. Multiple factors, such as the temperature, gas flow rate, pressure, residence time, equivalence ratio, biomass characteristics, and gasifier design, can influence the quality of gas generated through biomass GA. The air GA of biomass produces syngas that has a low H_2_ content and low heating value. Steam GA generates considerably more H_2_ compared to PY or air GA, with the overall thermal energy-to-H_2_ conversion efficiency potentially reaching as high as 52%. Given the H_2_% in gas (40%), increased H_2_/CO ratio (1.6), and fewer contaminants relative to air GA, steam GA is the optimal method for generating H_2_. Steam GA is suitable for wet biomass (moisture content between 5 and 35 wt.%), whereas air GA requires dry feedstock [405]. Biomass GA with O_2_-rich air is an effective method for generating gas with a medium heating value; however, its high O_2_ production costs hinder its widespread use. Steam GA processes utilizing a fluidized-bed reactor, either with or without added O_2_, are effective in generating MHVs of 10–16 MJ N−1 m^3^ gas containing 30–60 vol% H_2_. This is due to the lack of N_2_ in air GA products and the potential for a homogeneous WGS reaction occurring early in the GA process to enhance H_2_ production. However, such a method needs a steam temperature above 700 °C, and high costs are incurred for high-performance steam generators.

The SR process is a concurrent purification method that reduces the carbon-to-hydrogen mass ratio (C/H) of syngas during steam gasification [67]. By fine-tuning the operational parameters of SR, the quantities of light hydrocarbons and tar that cause pipe corrosion and blockage due to polymerization and condensation have been reduced [406].

The partial oxidation (PO) scenario differs from the SR process. The reaction pathway remains the same as that of SR, with H_2_O replaced by O_2_ [407]. A characteristic is that reactions involving O_2_ are exothermic, which removes the need for an external energy source or the use of smaller reactors. Based on the O_2_ levels in the reactor, various reactions take place. PO is thoroughly documented for ethanol, the most comprehensively studied feedstock in the literature. As H_2_ is produced along with CO due to ethanol and diminished O_2_ concentrations, the reaction starts as endothermic and turns exergonic when the amount of O_2_ in the reactor increases. In this case, the PO reaction is auto-thermal, resulting in the generation of CO_2_. PO is conducted at reduced temperatures because of the highly exothermic nature of the process [408,409]. With reduced O_2_ levels, WGS processes might enhance the H_2_ yield from PO using steam. This reaction is slightly exothermic, and the H_2_O added to the system inhibits catalyst deactivation by decreasing coke formation, similar to SR [410,411]. Catalysts are also necessary in PO, similar to SR, though there is no external heat source in PO. 

Supercritical water gasification (SCWG) is a different thermochemical method that is more adaptable for various biomass types, including wet biomass (moisture > 35%), like carbohydrates and timber. To form a supercritical fluid, H_2_O needs to be heated above 374 °C and subjected to pressure exceeding 221.2 bars. Under such conditions, the dielectric constant of H_2_O and the number of H_2_ bonds both decrease. At elevated temperatures, organic compounds and gases mix well in supercritical H_2_O, speeding up their transformation. The entire procedure is endothermic and similar to reforming in the aqueous phase. In comparison to other GA methods, residence times may be quite brief (2–6 s), and the reaction can occur at a reduced temperature (600–650 °C) [412]. Nonetheless, maintaining these conditions demands significant energy levels, increasing costs and constraining scalability [304]. Consequently, no large-scale industrial system has been developed because the high pressure needed demands considerable capital and operational costs [413]. Specific technical issues (standardization of reactor design, waste neutralization, and comprehension of chemical dynamics) need to be tackled to generate commercially feasible SCWG systems [200]. SCWG systems operate within two temperature ranges: elevated temperatures, typically between 500 and 700 °C, and reduced temperatures, usually from 374 to 500 °C. PY techniques take place prior to gasification at elevated temperatures, breaking down the fuel without requiring a catalyst. Nevertheless, alkali catalysts may be utilized to enhance H_2_ generation by elevating WGS reactions. Hydrolyzed compounds are gasified and reduced at lower temperatures (374–500 °C) using alkali-based catalysts, transition-metal catalysts, and activated carbon, which are essential for efficient biomass-to-gas conversion [380,414]. Methanation occurs at lower temperatures and utilizes the H_2_ produced to form CH_4_, requiring charcoal or an alternative carbon-based catalyst to address this challenge.

Catalytic Supercritical Water Gasification (CSCWG) is also known as low-temperature catalytic gasification due to its operational temperature range of 350–600 °C. The catalyst, along with the reduced reaction temperature, minimizes the formation of char and tar. Resende et al. [415] explored and documented the use of metal catalysts for the SCWG of cellulose and lignin. Numerous researchers have performed laboratory tests, laying the foundation for the concept’s use at pilot and commercial levels.

Rauzan et al. [416,417,418] established the initial CSCWG pilot facility in San Diego at the General Atomics location to explore the previous findings from lab-scale research. It was found that the H_2_ production matched the results from laboratory experiments, yielding 10 g of H_2_ for every 100 g of feed [366]. Catalysts play a significant role in the H_2_O GA process, enhancing H_2_ production [419,420]. Watanabe et al. [421] found that the use of CSCWG resulted in a twofold increase in H_2_ production compared to non-catalytic supercritical gasification. For aromatic compounds, Park et al. [422] employed RuO_2_. This analysis demonstrated that RuO_2_ provided the complete activity mechanism for the K_2_CO_3_ catalyst. K_2_CO_3_ exhibited greater activity than Ca(OH)_2_ in the gasification of cellulose within CSCWG, even though it did not capture CO_2_ [423]. For many years, metallic Ni catalysts have been used in conventional fossil fuel GA and are now applied in biomass CSCWG [424]. The expense of H_2_ production is essential for technological advancement; a comprehensive assessment was performed considering all elements, and the price of H_2_ was determined to be 3.4 USD/kg for the hydrogasification of biomass wastes [425]. Zhang et al. [426] examined the literature concerning the energy efficiencies of the SCWG method and identified values between 0.04 and 42.05%.

The risk assessment for biomass GA plants was overviewed in [427], while explosion parameters and protection in such plants were discussed in [428].

Some information about hazards accompanying the use of catalyst materials is presented further when GSR technology is discussed.

According to [429], hazard evaluation is essential for a biomass GA facility, as it encompasses multiple dangerous elements. Set pair analysis (SPA) is a useful and efficient technique for hazard evaluation, yet it has drawbacks, such as the failure to show differences when data fall under the same hazard category, and the evaluation outcomes suffer from imprecision and inaccuracy. Not only are the generated biomass gas and biomass substances combustible, but the biomass gas also possesses explosive and toxic properties. This imposes multiple risks on the biomass GA facility that require evaluations. The authors developed an enhanced technique known as general set pair analysis (GSPA). The Connection Measure Degree (CMD), based on the cosine function, along with the Weighting Deviation Degree (WDD), Relative Membership Degree (RMD), and Comprehensive Index (CI), was introduced in GSPA. During GSPA, six assessment metrics were defined: the hourly production volume of biomass gas (m^3^/h), the CO volume fraction in the biomass gas (%), the lower explosive limit of the biomass gas (%), the air exchange rate (time/h), the pressure relief capacity (m^2^/m^3^) during incidents of fires and explosions at the biomass GA station, which depends on the pressure relief area (m^2^) and the biomass GA station volume (m^3^), and the volume of biomass materials stored at the GA station (m^3^). Nevertheless, the complete hazard causes of biomass GA facilities cannot be captured solely by the six evaluation indicators. Numerous factors can trigger fires, explosions, and poisonings in biomass GA facilities, including tar, human mistakes, and weather conditions, among others. Tar is particularly significant, as it represents the main challenge in biomass GA technology. However, the aforementioned causes were not the direct triggers of fires, explosions, and poisoning in biomass GA facilities. For instance, being one of the components of the generated biomass gas, tar can cause equipment blockages, which may subsequently result in equipment damage and gas leaks, and such gas leaks can immediately cause fires, explosions, and poisoning in biomass GA facilities. In summary, tar was an indirect factor in causing fires, explosions, and poisoning. Likewise, other factors like human mistakes and weather conditions are also indirect causes of fires, explosions, and poisoning. As noted earlier, the hazard evaluation of the biomass GA facility utilizing GSPA was conducted in relation to the direct causes of fires, explosions, and poisoning identified in this research, and this hazard evaluation can be viewed as a targeted assessment focused on the immediate causes. For continued research, indirect factors, such as tar, human mistakes, weather conditions, and others, can be taken into account to conduct a more thorough hazard evaluation for the biomass GA facility. A case study was conducted on two biomass GA facilities located in Shenyang City, Liaoning Province, in Northeast China. The results of the hazard assessment for the GSPA were compared to those of the SPA. The findings indicated that GSPA is a more efficient, precise, and accurate approach for the hazard evaluation of a biomass GA facility.

In the dry method, the existence of inorganic substances such as Cl in the gas phase results in significant corrosion issues. In the aqueous product phase of HT GA, these compounds can be found. This reduces the amount of cleaning required and minimizes corrosion issues with the product gases in subsequent processing. Nevertheless, achieving cost-effective isolation of these inorganic compounds to complete the nutrition cycle remains difficult.

One major downside of HT GA could be the requirement to heat up a large amount of H_2_O. The amount of heat needed to reach 600 °C might be more than the energy stored in the biomass if the H_2_O content is above 80% (g/g). This implies that a costly heat exchanger with a high level of effectiveness is necessary. This is especially relevant for supercritical H_2_O GA, where specific materials are necessary due to the elevated pressures and temperatures exceeding 600 °C [398].

The HT conditions and high pressure in APR make it difficult to advance aqueous-phase reforming. Nonetheless, the primary obstacles are low yields of desired products and rapid catalyst deactivation [430].

In the APR process, the alcohols are converted into adsorbed intermediates on the catalyst surface following dehydrogenation steps, and then the C-C or C-O bonds are broken. If unsuitable catalysts are used, the breaking of C-O bonds can produce persistent organic acids that are difficult to regenerate. These generated organic acids can cause damage to the equipment and deactivate the catalysts. Hence, catalysts that are efficient for the APR process need to show strong performance in breaking C-C bonds [430].

According to [431], the yield of the main PY and GA products is affected by the hemicellulose, cellulose, and lignin content. Increasing the cellulose content in biomass enhances the yield of the liquid pyrolysis product. The amount of pyrolysis gas depends largely on the proportion of hemicellulose, whereas the content of lignin conditions is the share of char in the total amount of PY products. Lowering the size of a biomass particle enhances the gas release and the concentrations of H_2_ therein. This increases the reaction surface area between the particle and a heated medium, thus intensifying heating and decomposition. Changing the biomass surface structure enhances the efficiency of C conversion and facilitates gas release. The particle size and surface structure have a less significant effect compared to temperature, catalyst addition, and steam/biomass ratio. H_2_ yield is enhanced with a temperature rise because of intensified GA and tar-cracking reactions. A steam medium was more efficient in maximizing the hydrogen yield than the air. Adding catalysts increased the H_2_ release and enhanced the gas quality by lowering the amount of H_2_O, removing O_2_, and cracking heavy aromatic structures.

Ghasemi et al. [32] noticed that the technical challenges of GA encompass product standardization, catalyst degradation, corrosion, blockage, and insufficient commercial application. Economic challenges to GA may involve substantial operating and investment expenses due to the requirement for elevated temperatures. Membrane reactors are capable of integrating H_2_ production methods, thus enhancing the efficiency of the TH process and alleviating these issues.

The most information about hazards related to the implementation of TC processes of biomass was found for GA, then pyrolysis, and the least for HT processes. In the case of the first two types, these hazards are related to the release of toxic gases. The last type of process may be related to the quality and quantity of post-process H_2_O and all of the processes with large amounts of energy involved.

Biomass GA is in the commercialized stage and its cost is in the range of 1.77–2.05 USD/kg. Biomass GA has drawbacks such as tar formation and feedstock impurity. The remedy for them is the requirement for carbonaceous materials. The process efficiency is in the range of 30–40% [180,432].

#### 4.2.4. Initiatives Focused on TC Methods

Thermochemical processing entails exposing biomass to particular temperatures and chemical environments, influencing both the quality and amount of H_2_ produced. The length of exposure is essential for total conversion and avoiding degradation. The type of biomass utilized has a significant effect on the efficiency of the conversion process. Feedstocks rich in carbohydrates, cellulose, and hemicellulose are favored because of their substantial H_2_ yields. Nonetheless, these feedstocks need pretreatment techniques like drying, size reduction, and catalyst incorporation to improve their reactivity and accessibility for the conversion process. Catalysts can be naturally present in the biomass or introduced from outside. The characteristics of the reactors have been shown to influence heat transfer, mixing, flow patterns, H_2_ yield, and its composition [182].

Li and Guo [270] investigated a co-precipitated Mg-promoted Ni/Al_2_O_3_ catalyst (Ni-Mg-Al) for the SCWG of various biomass models and real biomass, along with its stability and performance. The jointly participated catalyst demonstrated higher activity recoveries due to its stable Ni crystal size, based on their comparison of the Ni-Mg-Al catalyst and an impregnation method. Moreover, the research examined how SCWG variables influenced the gasification efficiencies of phenol and glucose, along with the non-catalytic and catalytic gas products. The results indicate that full carbon gasification of various organic substances, such as phenol and real biomass, was achievable with an adequate amount of Ni catalyst. Between 400 and 500 °C, the catalyst generated CH_4_, while between 500 and 600 °C, there were higher yields. Actual biomass was less effective than the co-precipitated Ni-Mg-Al catalyst in gasifying H_2_O-soluble organic materials.

This technology could be integrated with other renewable energy sources like solar and wind to form synergistic energy generation systems that utilize biomass resources for H_2_ production and energy storage. This procedure functions based on the principle of temperature regulation regarding certain product distributions [265]. Yao et al. [433] have demonstrated that biochar serves as an effective catalyst and gasification aid for biomass. The biochar produced from Corn Stover Pyrolysis (CSP) effectively interacts with volatiles because of the existence of alkali and alkaline earth metals, aiding in the conversion to H_2_. In SR, the introduction of Ni (Ni-based biochar) to a two-stage fixed-bed reactor at 800 °C led to a rise in H_2_ production from 45.91 to 92.08 mg H_2_/g biomass. 

Studies on gasification are ongoing, aimed at discovering the optimal catalysts and operating conditions to enhance gas quality and yield affordably. To promote tar conversion and prevent the formation of unwanted products, innovative and efficient catalysts are being implemented [265]. A variety of catalysts for thermochemical processes—especially gasification—have been analyzed and compared in research [282]. These typically consist of compounds reliant on nickel, olivine, potassium minerals, and dolomite [434]. Metal catalysts enhance the efficiency of gasification processes, leading to higher H_2_ yields from biomass. The selection of metal catalysts influences the conversion rates of biomass feedstocks, the generation of byproducts, and the effectiveness of H_2_ production. Fe-based composite catalysts in biomass steam gasification effectively generate H_2_-rich gas, underscoring the significance of choosing the right catalyst to optimize H_2_ production from biomass GA processes [435]. Metal catalysts, particularly Fe-based ones and other metals like Ni, Ru, Pt, Pd, Co, Cr, etc., significantly influence the H_2_ yield in the biomass hydrothermal gasification process by modifying reaction pathways, conversion rates, and the overall efficiency of H_2_ production.

Zhang et al. [395] found that employing limestone and dolomite at a 1:1 ratio as a catalyst yielded a peak output of 204.6 mL/kg of biomass within a temperature range of 450 to 850 ° C. Ma et al. [436] examined how different temperatures influence the H_2_ production of dolomite and olivine employed as catalysts. As the temperature rose from 700 to 900 °C, the tar production declined for dolomite, decreasing from 12.5 to 7.2 g/Nm^3^, and for olivine, from 15.9 to 9.1 g/Nm^3^. The tar conversion enhanced the H_2_ yield, rising from 36.2 to 46.4 g/kg and from 32.4 to 42.3 g/kg of biomass, respectively [395].

Chen et al. [437] examined Ni/CaAlO_x_ catalysts derived from wood sawdust through pyrolysis and SR techniques. Assuming a 1:1 molar ratio of Ca to Al, the catalysts generated 15.57 mmol of H_2_ for each gram of feedstock. Ca is a cost-effective alkaline metal that enhances CO selectivity, facilitating biomass conversion. A 90% concentration for CO was attained using a 3:1 Ca/Al ratio. Nonetheless, catalyst deactivation caused by coke deposition remains the restricting factor.

Xu et al. [438] investigated how alkaline earth metals affect GA efficacy. They discovered that calcined CaO containing 5-weight percent iron resulted in the greatest yield in a 700 °C fluidized-bed reactor for wood sawdust GA. Nonetheless, the catalyst Fe/CaO was not as efficient in CO_2_ capture and GA. Fe impregnation led to increased production, as it inhibits the deactivation of CaO. 

Some information about hazards related to the catalyst materials is presented further when GSR technology is discussed.

Integrating the enhanced GA method with biorefinery configurations for bio-based goods can assist in the sustainable generation of H_2_. Furthermore, it offers opportunities for resource recovery and waste management. Further research might enhance the ability to capture and utilize carbon dioxide, aligning with global efforts to decrease GHG emissions and tackle climate change [265].

### 4.3. The Hydrogen Extraction via Glycerol Steam Reforming and Hazards Related to It

C_3_H_8_O_3_, which is extensively produced as a byproduct in biodiesel production [439], is used in the production of H_2_ through the glycerol steam reforming (GSR) process [18], ref. [440] based on the stoichiometric point of view. GSR can produce 7 mol of H_2_ using 1 mol of C_3_H_8_O_3_. Thermodynamic and experimental research indicates that only 6–6.2 mol of H_2_ can be produced for each mol of C_3_H_8_O_3_ [441,442]. The manufacturing process of H_2_ relies on the choice of raw materials and techniques.

Current reforming systems suffer from disadvantages such as expensive production, reduced efficiency, GHG emissions, C deposition, and complexity [443].

Silva et al. [444] reported that intensive research regarding GSR thermodynamics, catalysts, reaction mechanisms and kinetics, and new reactor design (sorption-enhanced reactors (SERs) and membrane reactors (MRs)) has been conducted, focusing on enhancing the process efficacy. In their important review, the main challenges and strategies adopted for optimization of the GSR process were discussed, such as the GSR thermodynamic aspects and advances in catalysis and kinetics, as well as GSR performed in SERs and MRs.

Three studies were discovered in the literature concerning hazards associated with the utilization of C_3_H_8_O_3_ (Table 9).

Exposure to C_3_H_8_O_3_ may cause irritation of the skin and eyes. Breathing in Glycer-ol-alpha-Monochlorohydrin may cause irritation in the nose and throat, leading to coughing and wheezing. Exposure to Glycerol-alpha-Monochlorohydrin may lead to feelings of nausea, vomiting, dizziness, lack of coordination, and, in severe cases, even coma [445].

C_3_H_8_O_3_ is flammable and produces irritating or toxic gases when burned. It should not be used near open fires [446].

Ingesting C_3_H_8_O_3_ orally is considered safe for short-term usage. Nevertheless, potential side effects include headaches, dizziness, bloating, nausea, and diarrhea. Applying C_3_H_8_O_3_ to the skin is not harmful. It can cause redness, itching, and burning [447].

SR techniques for generating H_2_ can also be applied to various other biomass-based materials like vegetable oils, agricultural production waste, C_3_H_8_O_3_, and biomass pyrolysis oil [448].

The process of steam reforming typically involves the utilization of a variety of catalysts.

Traditional SR catalysts are fairly inexpensive, but they are prone to being affected by S-based catalyst poisons. 

The production of H_2_ by GSR is greatly impacted by the catalyst’s ability to break C–H, C–C, and C–O bonds, as well as by the processing conditions. Currently, there is a strong focus on creating an effective catalyst that can efficiently utilize raw C_3_H_8_O_3_ for high performance. Different catalysts using metals such as Pt, Rh, Re, Pd, Ru, Ir, Co, Cu, and Ni were examined. Nonetheless, the advancement of Ni-based catalysts, particularly those utilizing Ni-based alumina, has been more beneficial in recent times. Another intriguing possibility is the utilization of perovskite-type and hydrotalcite-based catalysts [449].

Naranje et al. [450] created and simulated an integrated method for producing biodiesel from used cooking oil while optimizing C_3_H_8_O_3_ by converting it to H_2_. They discovered that the biodiesel yield reached 92.5%, while the H_2_ output from the reforming process was approximately 13%.

A key challenge in producing H_2_ from C_3_H_8_O_3_ is attaining a higher yield. Numerous improvements have been made in addressing this issue, yet only a handful are prepared for market launch [451]. A study was conducted on a compilation of recoveries obtained through the SR method for H_2_ production from crude C_3_H_8_O_3_, revealing that multiple processes demonstrated recoveries of 3% [452]. The most significant study involved examining a commercial Ni catalyst that enabled a yield of 70% [453]. The majority of processes yielding over 70% are improved by significant quantities of catalysts, including Pt, Ru, Ni, Ce, and various other rare compound catalysts [452]. These need to be improved with various compounds, primarily metal oxides. Nonetheless, considerable effort must be invested to advance technologies for reforming crude C_3_H_8_O_3_ into H_2_ and their real-world commercialization.

#### 4.3.1. Catalysts

The literature identified four dangers related to the use of Pt and its complexes (Table 9).

While Pt metal itself is non-combustible, finely ground platinum powder can be both flammable and explosive, and toxic fumes are released if it catches fire. Coming into contact with platinum can cause irritation to the skin and eyes. Inhaling it may cause irritation in the nose and throat. Exposure to platinum might lead to a skin allergic reaction [454,455].

As documented in [456], Pt is classified as a flammable solid with hazard code H228.

Contact with H_2_O can cause Pt powder to react quickly or explosively. Friction, heat, sparks, or flames can all potentially trigger its ignition. The dust or fumes it produces could create explosive mixtures in the air [456].

Pt coordination complexes are capable of causing cancer and damaging genetic material in both mammalian and bacterial cells [457].

Additionally, four risks were identified in studies regarding dangers linked to the utilization of Rh and its substances (Table 9).

Rh is not flammable as a solid but becomes flammable when in dust or powder form, and toxic gases are emitted during a fire. Inhaling Rh powder can affect humans. Skin and eyes may become irritated when exposed to Rh powder. It could trigger a skin reaction. If an allergy emerges, even minimal future contact can result in itching and a skin rash [458].

All compounds containing Rh are extremely poisonous and can induce cancer. They leave a very noticeable mark on the skin. Flammability is a possibility. A dust explosion may happen when in powder or granular form and combined with air [459].

Improperly managed, Rh can pose risks to both human health and the environment. For example, rhodium solutions usually have rhodium sulfate, which is harmful and can lead to serious skin and eye irritation when touched, along with breathing issues if breathed in [460].

Rh is a catalyst and can react with various organic and inorganic substances, potentially leading to fire and explosion danger [461]. 

Four bits of data were found in the literature concerning risks associated with the usage of Re and its compounds (Table 9).

Exposure to Re may lead to skin irritation. The liquid form could cause skin and eye burns. Following consumption, it could lead to irritation in the gastrointestinal tract. Inhaling it can result in irritation of the respiratory tract [462].

It is a material that is not flammable, but it can self-ignite when exposed to alcohol or similar organic substances. Re dust could lead to slight to mild irritation in the eyes and skin. Inhaling fine Re dust or fumes can cause irritation in the nasal cavity and respiratory tract [463].

The substance causes physical irritation in the gastrointestinal tract. There have been no reports of toxic effects from Re in humans or animals [464].

Contact between Re oxide and flammable materials can result in fire due to its oxidizing properties [465].

Nine literature reviews discovered concerning the risks associated with the utilization of Pd (Table 9).

Pd is a substance with low toxicity levels and is not easily absorbed by the body if consumed. It can lead to skin, eye, or respiratory irritation, as well as skin sensitization. Liquid can result in skin and eye burns.

All compounds with Pd are extremely toxic and can cause cancer. Palladium chloride, for instance, is poisonous and can be dangerous if ingested, inhaled, or absorbed through the skin. It causes harm to the bone marrow, liver, and kidneys of animals in laboratory experiments. It induces irritation.

Pd shows minimal impact on its surroundings. Low concentrations of palladium salts are lethal to the H_2_O hyacinth, although the majority of plants can withstand levels under 3 ppm [466].

Pd powder is considered a solid that can easily catch fire (H228). It results in irritation to the skin (H315) and serious irritation to the eyes (H319) and can also lead to irritation of the respiratory system (H335) [467].

Contact dermatitis can be induced by Pd alloy [468].

Pd can catch fire when coming into contact with air, especially if it contains absorbed H_2_. It easily triggers the combustion of flammable solvents when in contact with air. Combustible properties are only found in Pd fine powder or dust. Many compounds of palladium act as oxidizing agents, with some reacting violently with organic substances.

Pd has low toxicity in short-term contact but becomes highly toxic over a longer period, especially at the cellular level in the liver and kidney. Increased Pd levels can be harmful and potentially cancer-causing for mammals.

Continual exposure to Pd particles in dust can cause harm to the blood and respiratory systems. With Pd on C catalysts, the small C particles can cause irritation to the mucous membranes and upper respiratory tract.

Pd on C catalysts become highly flammable when dry and at high temperatures, especially with adsorbed H_2_ gas. Catalysts made by reducing formaldehyde are not as flammable as those reduced with H_2_. C particles that are broken down into smaller pieces have the potential to cause a dust explosion.

Catalysts created on supports with a large surface area exhibit high activity and readily initiate ignition of mixtures of H_2_/air and solvent/air. Because of its high volatility, CH_3_OH readily catches fire. The use of a catalyst in a H_4_B solution can trigger the ignition of released H_2_ [469].

Only two instances of dangers have been discovered in the literature concerning the utilization of Ru and Ir (Table 9).

All Ru compounds are extremely dangerous and can cause cancer. They cause significant staining on the skin. Ru consumed is firmly retained in bones. Ruthenium oxide, also known as RuO_4_, is extremely dangerous and easily evaporates, so it should be steered clear of [470].

Ru is a solid that can easily catch fire (H228) [471]. 

Ir is extremely combustible. It has the potential to induce irritation in the eyes. It poses a minimal risk to the skin for standard industrial handling. Ingesting it can lead to irritation of the digestive tract [472].

Extracting and processing Ir has negative environmental impacts. Extracting and processing Ir has a negative impact on ecosystems and adds to carbon emissions [473].

Three literature reviews were discovered concerning the dangers associated with the use of Co and its compounds (Table 9).

Being exposed to Co dust can cause irritation to the skin, eyes, nose, and throat. Co can potentially trigger an allergic reaction similar to asthma. In the event of the latter, being exposed to the future can lead to asthma attacks that result in shortness of breath, wheezing, coughing, and/or chest tightness. Co might lead to problems in the heart, thyroid, liver, and kidneys [474].

Co is a vital cofactor present in the body, commonly found in nutrients like vitamin B12. In large quantities and in its non-organic form, Co can be very harmful. Excessive nutritional supplementation can lead to rare cases of acute toxicity [475].

Soluble Co salts negatively impact cell division, permanently attach to nucleic acids in the cell nucleus, cause chromosome abnormalities in plants, and exhibit weak mutagenic effects in certain in vitro tests involving cultured animal cells, bacteria, and yeast [476].

Eight bits of data were discovered in the literature concerning the dangers associated with the utilization of Cu (Table 9).

Consuming excessive daily doses of Cu for an extended period can result in serious health issues, like kidney and liver damage. Breathing in Cu dust, sprays, or crystals may lead to nose and throat irritation, as well as dizziness and headaches [477].

Cu that is finely divided can ignite or detonate when exposed to air. Toxic fumes are created during a fire.

Exposure to Cu powder may cause skin and eye irritation and burns.

Inhaling Cu can cause irritation in the nose and throat, as well as potentially creating a sore or hole in the septum of the inner nose. Copper can cause headaches, nausea, vomiting, diarrhea, and abdominal pain. Exposure to copper can result in sickness similar to the flu, known as “metal fume fever”. Cu can lead to a skin allergic reaction and impact the function of the liver and kidneys.

As per OSHA, the allowable airborne exposure limit (PEL) is 1 mg/m_3_ (as Cu dust and mists) and 0.1 mg/m^3^ (as Cu fume) over an 8 h work shift [478].

Being exposed to elevated levels of copper can have negative effects. Prolonged exposure to copper dust may lead to nose, mouth, and eye irritation, as well as headaches, dizziness, nausea, and diarrhea [479].

Excessive Cu consumption can lead to liver damage, stomach pain, cramps, nausea, diarrhea, and vomiting. Toxic levels of Cu can develop in individuals diagnosed with Wilson’s disease, an uncommon inherited condition [480].

Cu does not degrade in the environment, leading to its accumulation in plants and animals from soil. Only a small number of plants are able to live in soil rich in Cu. Hence, the number of plant species is restricted in the vicinity of Cu-disposing factories [481].

The literature contained two pieces of information about the risks associated with the use of Ni (Table 9).

The most widely used catalyst in reforming reactions is the Ni-based catalyst due to its ready availability, cost-effective nature, and strong catalytic activity. Ni also showed high intrinsic reactivity and was easily dispersed on support materials. Over the years, various methods of production and catalyst design have been discussed in the literature, but none have specifically addressed the advantages, disadvantages, limitations, and difficulties encountered in C_3_H_8_O_3_ reforming reactions catalyzed by Ni-based catalysts.

The activated catalyst Raney Nickel, a 50% slurry in H_2_O, is capable of self-heating and may potentially ignite (H251). It has the potential to induce an allergic skin response (H317). It is believed to cause cancer (H351). It can cause harm to the organs when there is prolonged or repeated exposure (H372). It is detrimental to under-H_2_O organisms with enduring consequences (H412) [482].

Carrero et al. [483] reviewed numerous studies comparing catalysts featuring various active phases for H_2_ generation via SR. It was found that, despite extensive research on Ni catalysts, the exploration of Co catalysts has been relatively limited, even though Co-based catalysts offer excellent catalytic activity at moderate temperatures, enhancing H_2_ production through the favored WGS reaction. Nonetheless, the primary disadvantage of Co catalysts is their deactivation due to surface oxidation and the sintering of Co metal species. Such a final point is significantly influenced by the selection of the catalytic support since it is noted that cobalt interacts vigorously with Al_2_O_3_ and TiO_2_ carriers, resulting in a high dispersion of Co species; however, the formation of cobalt aluminates and titanates leads to a reduction in the amount of available Co species. In contrast, a less strong interaction between Co and a Si support enhances the reducibility of cobalt oxides but encourages the agglomeration of Co particles throughout the calcination and reduction processes.

As the interaction between the metal and support is crucial for the dispersion and reducibility of metallic species, the large-surface-area feature of mesostructured materials such as SBA-15 should enable achieving superior dispersions in comparison to traditional amorphous silica. Moreover, having a consistent pore-size distribution in SBA-15 might prevent the creation of large Co clusters and the deactivation of catalysts due to metal sintering. 

#### 4.3.2. Promotors

Various effects on catalytic performance are induced by diverse promoters (K, Ca, Sr, Ce, La, Cr, Fe) and process conditions. Specifically, Ce, Mg, and La significantly affect catalytic performance when used as promoters. Furthermore, GSR using hydrotalcite- and perovskite-based catalysts showed excellent catalytic activity, along with enhanced thermal stability and resistance to coke formation. Specifically, Ni/LaNi_0.9_Cu_0.1_O_3_ synthesized with perovskite-type supports has displayed high C_3_H_8_O_3_ conversion and adequate H_2_ selectivity at reduced temperatures. Catalysts similar to hydrotalcite have demonstrated increased stability in catalysis because of their strong resistance to high temperatures and minimal formation of coke [449].

The use of K as a promotor is accompanied by several hazards, as shown in Table 9. 

Coming into contact with solid potassium can lead to serious burns. Inhaling potassium fumes can cause irritation in the nose, throat, and lungs, leading to symptoms like sneezing and coughing. Continuous inhalation of K fumes can lead to lesions in the nasal passages. K is a chemical that is both flammable and reactive, which poses risks of fires and explosions [484]. 

Total K intake should not exceed the recommended amounts. In some cases, too much potassium may cause muscle weakness, confusion, irregular heartbeat, or difficulty breathing [485].

Signs related to neuromuscular dysfunction, such as weakness, paralysis, nausea, vomiting, and diarrhea, have been linked to the excessive intake of K. These symptoms do not always appear before dangerous cardiac arrhythmias occur [486].

The use of Ca as a promotor is accompanied by several hazards (Table 9).

Ca can react violently with H_2_O, steam, moisture, and strong acids, including hydrochloric, sulfuric, and nitric, to form flammable and explosive H_2_ gas. Finely divided calcium can ignite in air or in contact with halogens, including chlorine and fluorine [487].

Elevated levels of C in the bloodstream and urine can lead to weak muscle tone, impaired kidney function, decreased phosphate levels, constipation, nausea, weight loss, excessive fatigue, frequent urination, irregular heartbeats, and an increased likelihood of dying from heart issues [488].

The human body requires a large amount of C in CaCO_3_, not in its pure metallic state. Pure C is highly reactive with H_2_O and has the potential to cause damage to the tongue and esophagus [489].

CaCO_3_ is not highly toxic. On the other hand, elevated levels of Ca can lead to severe disruptions in heart rhythm, as well as the formation of kidney stones and harm to kidney function. Long-term excessive usage is typically more severe than a one-time overdose [490].

Three studies in the literature discussed the dangers linked to the utilization of Sr (Table 9).

Inhaling Sr can harm people. Skin and eye irritation may result from it. Extended contact with Sr can potentially impact the heart. High levels of Sr exposure can lead to accumulation in the bones and potentially impact their function [491].

Being exposed to high levels of radioactive Sr can lead to the development of cancer. Leukemia was present in individuals who were exposed to large doses of radioactive strontium. Lab animals also developed leukemia and cancers in their bones, nose, lungs, and skin [492].

Sr is non-combustible but produces flammable gas when exposed to H_2_O or moist air [493].

Four bits of data were found concerning the dangers associated with the use of Ce and its compounds (Table 9).

Ce is a solid substance that can easily catch fire (H228). It ignites due to friction and combusts in fiery situations. Under conditions of fire, it could potentially react with H_2_O and release H_2_ gas. Acid exposure can also produce H_2_ gas [494].

Exposure to CeO_2_ can lead to irritation of the eyes and damaged skin. It can also lead to lung irritation. These compounds have varying levels of toxicity, ranging from mild to moderate. In a study on animals, Ce_2_(CO_3_)_3_, CeF_3_, and CeO_2_ were found to not be immediately toxic, did not cause skin irritation, and caused only minimal irritation to the eyes. CeCl_3_ was found to be more toxic in terms of acute effects and was also a strong irritant to the skin [495].

A workplace exposed to Ce is mainly hazardous because of the presence of damps and gases that can be breathed in along with the air. This can result in pulmonary embolisms, particularly with prolonged exposure. Accumulation of Ce in the human body can pose a threat to the liver [496].

Ce NPs showed high levels of toxicity in all the toxicity tests performed (inhibition above 80% at low concentrations in the bioluminescence test and LC50 = 0.012 mg/mL in *Daphnia magna* assays) [497].

Four studies on the risks associated with utilizing La and its compounds were found (Table 9).

La can ignite when in contact with heat, sparks, or flames. It reacts strongly with acids and can also react with H_2_O during fire situations, producing flammable H_2_ gas in both scenarios. Normal handling and the use of solid forms of this material pose minimal health risks. Operations like grinding, melting, or welding can create dangerous dust or fumes that may be inhaled or come into contact with the skin or eyes [498].

La does not pass through the undamaged blood–brain barrier. Common side effects include mild to moderate feelings of sickness, frequent bowel movements, and gas [499].

The use of Fosrenol (La_2_(CO_3_)_3_) medication may cause serious stomach or bowel problems, including blockage or perforation (tear or hole) or severe constipation [500].

Fosrenol can cause blockage of the stomach, intestines, or rectum, which can be very dangerous. The risk is higher in persons with a history of changes to the digestive tract’s anatomy or constipation problems or who are taking medications that can also cause blockage [501].

Six studies in the literature discussed the risks linked to the use of Cr (Table 9).

Being exposed to Cr(VI) can lead to the development of work-related asthma, irritation and harm to the eyes, perforated eardrums, irritation of the respiratory system, damage to the kidneys and liver, congestion and swelling of the lungs, pain in the upper abdomen, irritation and harm to the nose, cancer of the respiratory system, irritation of the skin, and erosion and staining of the teeth. Certain employees may experience an allergic skin response known as allergic contact dermatitis. This is evident when working with liquids or solids that have Cr(VI), such as Portland cement. This type of dermatitis becomes prolonged and worsens with continued contact with the skin. Coming into contact with skin that is not whole can lead to the development of skin ulcers, also known as chrome ulcers. Crusted ulcers in chrome are lesions that are painless and have a pitted ulcer covered with fluid [502].

Continued exposure to Cr(VI) compounds via breathing raises the likelihood of developing lung, nasal, and sinus cancer. Contact with Cr(VI) compounds can lead to severe dermatitis and typically painless skin ulcers. Cr compounds can act as both sensitizers and irritants [503].

Exposure to different oxidation states of Cr results in varying levels of health hazards, with the metal form being less toxic and the hexavalent form being more toxic [504].

The process of Cr plating is linked to various dangers. Cr contains Cr(VI), which is a proven cancer-causing agent. Lead is used in the plating process and can be absorbed through the skin, potentially leading to liver, organ, and brain harm. Cyanide is extremely poisonous. It is utilized during Cr plating and has the potential to be lethal to humans. Additionally, the Cd utilized in the procedure has the potential to result in cancer as well as kidney and lung damage [505].

Cr is a significant pollutant found in many dangerous waste locations globally, such as the Superfund sites in the US [506].

Employees in sectors that utilize Cr have an increased likelihood of experiencing the negative health impacts associated with Cr [507].

Two bits of information were discovered in the literature regarding the utilization of Fe (Table 9).

Fe powder or dust is a solid material that can easily catch fire. It could result in irritation in the eyes and skin due to mechanical reasons. It could lead to abnormalities in the blood. It can also lead to injury in the lungs. Breathing in fumes can result in metal fume fever. It could lead to heart complications and harm the liver. Fe powder, if consumed, can lead to irritation in the gastrointestinal tract accompanied by symptoms such as nausea, vomiting, and diarrhea. Pancreatic damage, diabetes, and cardiac abnormalities can be caused by frequent exposure [508].

Excessive intake of Fe by humans can result in severe symptoms, liver damage, and potentially death. Symptoms progress through different stages, starting with vomiting, diarrhea, and abdominal pain. Liver failure may occur several days later [509].

**Table 9:** Hazards related to GSR.

Material Category	Substance	Information on Hazards	Refs.
Substrate	C_3_H_8_O_3_	Coming into contact with it can result in skin and eye irritation. Inhaling Glycerol-alpha-Monochlorohydrin may lead to irritation in the nose and throat, resulting in coughing and wheezing. Exposure to Glycerol-alpha-Monochlorohydrin may lead to nausea, vomiting, dizziness, lack of coordination, and potentially coma. The mitigation of hazards comprises the following measures: Avoid skin contact and eye contact with such material. Wear protective clothing, gloves, and goggles. Use a proper respirator during enhanced exposure to such a material.	[445]
		It is flammable (H228) and emits irritating or toxic fumes (or gases) when exposed to fire. The mitigation of hazards comprises the following measures: It should not be used in the presence of open flames. Wear protective clothing, gloves, and goggles. Provide enough ventilation.	[446]
		When taken by mouth, headaches, dizziness, bloating, nausea, and diarrhea might occur. When applied to the skin, it might cause redness, itching, and burning.	[447]
Catalyst	Pt	Highly fragmented Pt powder can ignite easily, explode, and emit toxic fumes when burned. Coming into contact with it may cause irritation to the skin and eyes. Inhaling it may cause irritation to the nose and throat. It could lead to a skin rash. The mitigation of hazards comprises the following measures: Avoid skin contact and eye contact with Pt. Wear protective clothing, gloves, and goggles.	[454,455]
		Pt powder is a flammable solid (H228). Pt powder may react violently or explosively upon contact with H_2_O. It may be ignited by friction, heat, sparks, or flames. Its dust or fumes may form explosive mixtures in the air. The mitigation of hazards comprises the following measures: Wear protective clothing, gloves, and goggles. Provide enough ventilation. Eliminate all ignition sources (no smoking, flares, sparks, or flames). Do not touch or walk through spilled material. Stop the leak if you can do so without risk.	[456]
		Pt coordination complexes are carcinogenic and genotoxic in mammalian and bacterial cells.	[457]
	Rh	It is flammable when in dust or powder form, and toxic gases are generated during a fire. Breathing in Rh powder can have an impact on humans. Exposure to Rh powder may cause irritation to the skin and eyes. It has the potential to lead to a skin allergic reaction. If an allergy develops, even minimal future exposure can lead to itching and a skin rash. The mitigation of hazards comprises the following measures: Avoid skin contact and eye contact with Rh. Wear protective clothing, gloves, and goggles. Eliminate all ignition sources (no smoking, flares, sparks, or flames). Provide enough ventilation.	[458]
		All Rh compounds are highly toxic and carcinogenic. They stain the skin very strongly. They can be flammable. A dust explosion can occur if the Rh powder or granular form is mixed with air. Mitigation of hazards: In addition to those previously mentioned, do not allow the material to be released into the environment.	[459]
		Rh, when not managed effectively, can have negative effects on both human health and the environment. Rh solutions, which include Rh_2_(SO_4_)_3_, can be harmful, causing skin and eye irritation and respiratory issues if breathed in.	[460]
		Rh is a catalyst and can result in a combustible and explosive danger when exposed to various organic and inorganic substances. Mitigation of hazards: In addition to those previously mentioned, use local exhaust or breathing protection. Remove inappropriate substances from places with Rh present.	[461]
	Re	Re can lead to skin irritation. Its liquid state has the potential to inflict burns on the skin and eyes. Following consumption, it can lead to irritation in the digestive system. Inhaling it could result in irritation of the respiratory tract.	[462]
		If exposed to alcohol or similar organic substances, it could spontaneously combust. The Re dust can result in minor to moderate eye and skin irritation. Breathing in fine Re dust or fumes can cause irritation in the nose and lungs. The mitigation of hazards comprises the following measures: Avoid skin contact and eye contact with Rh. Wear protective clothing, gloves, and goggles. Provide enough ventilation. Use proper respiratory protective equipment. If used as a catalyst, keep the spent catalyst away from combustibles, as they can ignite.	[463]
		Re is a flammable solid. It is a physical irritant to the gastrointestinal tract. The mitigation of hazards comprises the following measures: Keeping away from heat, hot surfaces, sparks, open flames, and other ignition sources. No smoking is allowed (P210) Grounding/bonding container and receiving equipment (P240). Using explosion-proof electrical/ventilating/lighting/intrinsically safe equipment (P241). Wearing protective gloves/protective clothing/eye protection/face protection (P280).	[464]
		Re_2_O_7_ is an oxidizer, and its contact with combustible material may cause fire. Its inhalation may lead to irritation of the respiratory tract. It may lead to irritation of the nose, throat, and lungs and potential chemical burns to mucous membranes. If swallowed, it may lead to chemical burns in the mouth, throat, and digestive system. When in contact with skin, it is a strong irritant and corrosive. It may result in intense irritation and chemical burns. In contact with the eye, it is a corrosive substance and a powerful irritant. It may result in chemical burns to eye tissue and visual disturbances, including blindness. The mitigation of hazards comprises the following measures: Wear protective clothing, gloves, and goggles. Provide enough ventilation. Use proper respiratory protective equipment. Remove combustible materials from contact with Re.	[465]
	Pd	When consumed, the body does not absorb Pd well. It can lead to irritation of the skin, eyes, or respiratory tract and can also cause skin sensitization. Liquid has the potential to inflict burns on the skin and eyes. All compounds containing Pd are extremely harmful and can cause cancer. PdCl_2_ is poisonous and can cause harm if ingested, breathed in, or taken in through the skin. It results in harm to the bone marrow, liver, and kidneys of lab animals. It is irritating. Low concentrations of Pd salts can eliminate the H_2_O hyacinth, while most plants can withstand levels under 3 ppm.	[466]
		Powdered Pd is a solid that can catch fire easily (H228). It results in skin irritation (H315) and severe eye irritation (H319), possibly leading to respiratory irritation (H335). The mitigation of hazards comprises the following measures: P210: Avoid heat, sparks, open flames, and hot surfaces. Smoking is not allowed. P280: Use protective gloves and eye protection. P302+P352: If in contact with skin, rinse thoroughly with ample H_2_O. P305+P351+P338: If in eyes, rinse carefully with H_2_O for a few minutes. Take out contact lenses if they are in place and simple to remove. Keep rinsing. P312: Contact a poison center/doctor if you feel sick.	[467]
		Solid-state Pd alloys are typically considered non-hazardous. Nonetheless, if the procedure includes grinding, melting, cutting, or any other method that results in the emission of dust or fumes, dangerous quantities of airborne particulates may be produced. Contact dermatitis can be caused by Pd alloy. In case of dust or fume generation, the following measures should be taken: Wear protective clothing, gloves, and goggles. Use a proper respirator during enhanced exposure to such a material. Provide enough ventilation.	[468]
		Pd powder can catch fire when coming into contact with air, especially if there is adsorbed H_2_ present. It easily ignites flammable solvents when they are exposed to air. Pd can catch fire easily in the form of fine powder or dust. Many of Pd’s compounds act as oxidizing agents, and others can react explosively with organic materials. Pd exhibits high toxicity over an extended period and at the cellular level in the liver and kidney. Elevated levels of Pd can be toxic and potentially cancer-causing for mammals. Long-term exposure to Pd particles found in dust can cause harmful impacts on the blood and respiratory systems. With Pd/C catalysts, the finely divided carbon can cause irritation to mucous membranes and the upper respiratory tract. Pd/C catalysts with absorbed H_2_ can ignite easily, especially when devoid of moisture and exposed to high temperatures. Catalysts prepared by reducing CH_2_O are not as pyrophoric as those reduced with H_2_. C in a fine powder form has the potential to cause a dust explosion. Catalysts made on supports with a large surface area are very effective and easily ignite mixtures of H_2_/air and solvent/air. CH_3_OH is highly volatile, which makes it prone to easily catch fire. The introduction of a catalyst into a H_4_B solution could lead to the combustion of the released H_2._	[469]
	Ru	All Ru substances are extremely poisonous and can cause cancer. They leave a very intense stain on the skin. Ru that is consumed is firmly held in bones. RuO4 is extremely dangerous and easily evaporates, so it should be stayed away from. Mitigation of hazards: Avoid the ingestion of Ru and its contact with skin and eyes.	[470]
		Ruthenium is a solid substance that can easily catch fire (H228). The mitigation of hazards comprises the following measures: P210: Avoid heat/sparks/open flames/hot surfaces. Prohibit smoking. P240: Ground/bond the container and receiving apparatus. P241: Employ explosion-proof electrical, ventilation, lighting, and equipment. P280: Use protective gloves/protective attire/eye safety/facial protection. P370 + P378: In the event of a fire, utilize dry sand, dry chemicals, or alcohol-resistant foam for extinguishing.	[471]
	Ir	Ir is very combustible. It might lead to irritation in the eyes. When consumed, it can lead to irritation of the gastrointestinal system. The mitigation of hazards comprises the following measures: P210: Avoid heat, hot surfaces, sparks, open flames, and other sources of ignition. No tobacco use. P305 + P351 + P338: In case of contact with eyes, gently rinse with H_2_O for several minutes. Take out contact lenses if they are in place and simple to remove. Keep rinsing.	[472,510]
		Extracting and processing Ir has negative impacts on the environment. Ir extraction and processing have a negative impact on ecosystems and add to carbon emissions. The mitigation of hazards comprises the following measures: Recycling is a method to reduce the effects of Ir shortage. As the catalyst employed in electrolysis does not get exhausted, when the electrolyzer has completed its lifespan, the Ir can be retrieved and utilized in future uses. It is essential to reduce or fully remove the use of Ir in electrolyzers by choosing/studying alternative catalyst materials that offer comparable performance to Ir but at a reduced cost and greater availability to substitute Ir in traditional electrolyzers.	[473]
	Co	Coming into contact with Co dust can cause irritation to the skin, eyes, nose, and throat. Co may trigger an asthma-like allergic reaction, leading to asthma attacks characterized by difficulty breathing, wheezing, coughing, and chest tightness upon subsequent exposure. Co may impact the heart, thyroid, liver, and kidneys. The mitigation of hazards comprises the following measures: Limit or avoid contact with Co in all forms. In case of Co dust generation, take the following measures: Wear protective clothing, gloves, and goggles. Provide enough ventilation. Use proper respiratory protective equipment.	[474]
		Excessive amounts of inorganic Co can lead to considerable toxicity. Infrequent occurrence of acute toxicity can be attributed to the excessive intake of nutrients. Mitigation of hazards: Avoid excessive intake of nutrients with Co.	[475]
		Soluble Co salts have a negative impact on cell division, permanently attach to nucleic acids in the nucleus, cause chromosome abnormalities in plants, and show mild mutagenic effects in certain in vitro tests with cultured animal cells, bacteria, and yeast.	[476]
	Cu	Consuming high levels of Cu can result in serious health issues, including kidney and liver damage. Inhaling Cu dust, sprays, or crystals can result in nasal and throat irritation, as well as lead to dizziness and headaches. The mitigation of hazards comprises the following measures: Consistently clean or flush the Cu pipe system to reduce the amount of Cu that enters the H_2_O. Avoid using acidic H_2_O. Test whether H_2_O is acidic or whether copper concentrations in H_2_O are elevated. Keep copper powders, crystals, or dusts securely stored away from children, pets, or other adults. Keep an eye on Cu consumption if an intake via dietary supplements with Cu is increased to ensure that the recommended amount is not exceeded. When handling Cu, wear the required protective clothing and equipment and consistently adhere to safety protocols.	[477]
		Highly fragmented Cu can ignite or burst in the presence of O_2_. Toxic fumes are generated during a fire. Exposure to Cu powder may cause skin and eye irritation and burns. Breathing Cu can lead to irritation in the nose and throat, possibly resulting in a sore or perforation in the septum of the inner nose. Cu can lead to headaches, nausea, vomiting, diarrhea, and abdominal pain. Exposure to Cu can result in a flu-like sickness known as “metal fume fever”. Cu has the potential to trigger a skin allergy and impact the functioning of the liver and kidneys. The mitigation of hazards comprises the following measures: Avoid contact with Cu fumes and oxides. Remove oxidizing agents from contact with Cu powder. Provide enough ventilation. When handling Cu powder, wear the required protective clothing and equipment and consistently adhere to safety protocols.	[478]
		Being exposed to increased levels of Cu can be damaging. Prolonged exposure to Cu dust can lead to irritation of the nose, mouth, and eyes, as well as headaches, dizziness, nausea, and diarrhea. It is necessary to avoid prolonged exposure to Cu dust.	[479]
		Excessive consumption of Cu regularly can lead to liver damage, abdominal pain, cramps, nausea, diarrhea, and vomiting. Cu toxicity may develop in individuals with Wilson’s disease, an uncommon genetic condition. The level of Cu in diet should be controlled and Cu overdosing is prohibited.	[480]
		Cu does not degrade in the environment, leading to its accumulation in plants and animals from the soil. Only a few plants can thrive on Cu-rich soil. Hence, the presence of Cu-disposing factories restricts plant diversity in the surrounding area. It is necessary to systematically control the Cu level in the soil.	[481]
	Ni	The activated catalyst Raney Nickel in a 50% slurry of H_2_O can spontaneously combust, leading to a potential fire risk (H251). It can result in a skin allergy (H317). There are suspicions that it may lead to cancer (H351). It can also harm organs with long-term or repeated contact (H372). Harming aquatic organisms with lasting consequences (H412) is detrimental. The mitigation of hazards comprises the following measures: P201: Obtain special instructions before use. P235: Keep cool. P280: Wear protective gloves/protective clothing/eye protection/face protection. P302 + P352: In case of contact with skin, wash with plenty of soap and H_2_O. P308 + P313: If exposed or concerned, seek medical advice/attention. P420: Store separately.	[482]
Promotor	K	Coming into contact with solid K can result in serious burns. Inhaling K fumes can cause irritation to the nose, throat, and lungs, resulting in sneezing and coughing. Extended exposure to K fumes can lead to ulcers in the inner nasal passages. K is a chemical that is both flammable and reactive, posing risks of fire and explosions. The mitigation of hazards comprises the following measures: Remove oxidizing agents and H_2_O from contact with K. Provide enough ventilation. Avoid skin contact and eye contact with K. When handling Cu powder, wear the required protective clothing and equipment and consistently adhere to safety protocols.	[484]
		In some cases, too much K may cause muscle weakness, confusion, irregular heartbeat, or difficulty breathing. The K level in the diet should be controlled.	[485]
		Excessive consumption of K led to issues with neuromuscular functioning such as weakness, paralysis, nausea, vomiting, and diarrhea. These symptoms do not always appear before life-threatening heart rhythm disturbances. K overdosing is prohibited.	[486]
	Ca	Ca can have an explosive reaction with H_2_O, steam, moisture, and potent acids like hydrochloric, sulfuric, and nitric, resulting in the production of flammable H_2_ gas. Finely powdered Ca can catch fire when exposed to air or when coming into contact with halogens such as Cl and F. The mitigation of hazards comprises the following measures: Avoid Ca powder contact with air, Cl, and F. Avoid Ca contact with H_2_O, steam, moisture, and potent acids. When handling Ca powder, wear the required protective clothing and equipment and consistently adhere to safety protocols. Provide enough ventilation.	[487]
		High levels of Ca in the bloodstream and urine can lead to weak muscle tone, impaired kidney function, decreased phosphate levels, constipation, nausea, weight loss, severe fatigue, frequent urination, irregular heartbeats, and a significant risk of heart disease-related death.	[488]
		The human body requires an abundance of Ca in CaCO_3_, rather than in its pure metallic state. Ca reacts vigorously with H_2_O and can be harmful to the tongue and esophagus due to its corrosive nature. It is necessary to avoid direct Ca contact with the tongue and esophagus.	[489]
		Elevated levels of Ca can result in severe disruptions in heart rhythm, as well as the formation of kidney stones and the impairment of kidney function. Repeatedly using something for a long time can have worse consequences than taking too much of it at once. It is necessary to avoid the overconsumption of Ca and CaCO_3_.	[490]
	Sr	Inhaling Sr can have an impact on humans. It has the potential to irritate the skin and eyes. Extensive exposure to strontium could impact the heart. High levels of Sr exposure can lead to accumulation in the bones and potentially impact their functionality. The mitigation of hazards comprises the following measures: Avoid elevated levels of Sr exposure. Avoid contact between Sr and H_2_O. When handling Sr, wear the required protective clothing and equipment and consistently adhere to safety protocols.	[491]
		Exposure to significant levels of radioactive Sr may cause cancer. Leukemia has occurred in humans exposed to substantial amounts of radioactive Sr. Leukemia and cancers of the bone, nose, lung, and skin also occurred in laboratory animals. It is necessary to avoid elevated levels of radioactive Sr.	[492]
		Sr does not burn but produces flammable gas when exposed to H_2_O or moist air. It is necessary to avoid Sr contact with H_2_O or moist air.	[493]
	Ce	Ce is a solid that can easily catch fire (H228). It ignites via friction and combusts in fire situations. It could potentially release H_2_ gas when exposed to H_2_O during a fire situation. Exposure to acids can produce H_2_ gas. The mitigation of hazards comprises the following measures: Do not allow material to be released into the environment. Avoid heat, sparks, flames, and exposure to H_2_O or moist air. Use suitable respiratory and protective gear. Steer clear of touching skin and eyes. Steer clear of inhaling dust or smoke. Remove all sources of fire. Isolate the spill zone.	[494]
		Contact with CeO_2_ can lead to irritation of the eyes and abraded skin. It can also lead to irritation of the lungs. Compounds of Ce_2_(CO_3_)_3_ display varying degrees of toxicity, ranging from mild to moderate depending on the specific compound. In an animal study, Ce_2_(CO_3_)_3_, CeF_3_, and CeO_2_ did not show acute toxicity, exhibited no dermal irritation, and caused minimal eye irritation. CeCl_3_ showed higher acute toxicity and caused intense irritation to the skin. The mitigation of hazards comprises the following measures: Use suitable respiratory and protective gear. Keep away from skin and eyes. Avoid inhaling dust or fumes. Remove all sources of flames. Avoid sparks, heat, and fire. Isolate and contain the spill zone. Utilize an appropriate respirator in the presence of elevated concentrations. Wear safety goggles, impermeable gloves, and appropriate protective clothing as required. Manage under a dry inert gas like argon. Ce metal must be kept in well-sealed containers within argon or mineral oil.	[495]
		Ce is particularly hazardous in the workplace because of the presence of vapors and gases that can be breathed in along with air. This could lead to pulmonary embolisms, particularly with prolonged exposure. Accumulation in the human body can pose a threat to the liver. It is necessary to provide enough ventilation and avoid prolonged exposure to Ce and contact Ce with H_2_O.	[496]
		Ce NPs showed high toxicity in all toxicity tests performed (more than 80% inhibition at low concentrations in the bioluminescence test and an LC_50_ of 0.012 mg/mL in *Daphnia magna* assays). It is necessary to avoid the intake of CE NPs.	[497]
	La	La can become inflammable when exposed to heat, sparks, or flames. It reacts vigorously with acids and may react with H_2_O under fire conditions, in each case releasing flammable H_2_ gas. In regular use and handling, solid forms of this substance pose minimal health risks. Additional actions like grinding, melting, or welding can create dangerous dust or fumes that may be breathed in or contact the skin or eyes. The mitigation of hazards comprises the following measures: Use suitable respiratory and protective gear. Steer clear of contact with skin and eyes. Steer clear of inhaling dust or smoke. Remove all ignition sources. Secure the area affected by the spill. Do not permit entry into drains or discharge into the environment.	[498]
		La cannot pass through the unbroken blood–brain barrier. The most frequent negative reactions include slight to moderate nausea, diarrhea, and flatulence. The La amount in the human body should be controlled, and La overdosing and excessive exposure to La are prohibited.	[499]
		The use of Fosrenol (La_2_(CO_3_)_3_) medicine may cause serious stomach or bowel problems, including blockage or perforation (tear or hole) or severe constipation. The use of Fosrenol should be controlled, and its overdosing is prohibited.	[500]
		La compound (Fosrenol) can cause blockage of the stomach, intestines, or rectum, which can be very dangerous. The risk is higher in people with a history of changes to the digestive tract’s anatomy or constipation problems or who are taking medications that can also cause blockage. The use of Fosrenol should be controlled and carefully selected for each individual.	[501]
	Cr	Exposure to Cr(VI) can lead to occupational asthma, eye irritation and damage, perforated eardrums, respiratory irritation, kidney damage, liver damage, pulmonary congestion and edema, upper abdominal pain, nose irritation and damage, respiratory cancer, skin irritation, and erosion and discoloration of the teeth. Some employees may experience an allergic skin reaction known as allergic contact dermatitis. This is evident when dealing with liquids or solids that contain Cr(VI), such as Portland cement. This dermatitis persists for a long time and becomes more intense with frequent contact with the skin. Skin ulcers (chrome ulcers) can be caused by contact with damaged skin. Ulcers on the skin caused by chromium are characterized by crusty, painless sores with a depressed center filled with fluid. The mitigation of hazards comprises the following measures: Provide enough ventilation. When handling Cr(VI), wear the required protective clothing and equipment and consistently adhere to safety protocols. Limit or avoid exposure to liquids or solids that contain Cr(VI).	[495]
		Long-term exposure to Cr(VI) compounds increases the likelihood of developing cancer in the lungs, nasal passages, and sinus cavities. Contact with Cr(VI) compounds can lead to severe dermatitis and typically painless skin ulcers. Cr compounds can act as both sensitizers and irritants. It is necessary to avoid long-term exposure to Cr(VI) compounds.	[503]
		Exposure to different oxidation states of Cr results in varying levels of health hazards, with the metal form being less toxic and the hexavalent form being highly toxic.	[504]
		The process of Cr plating involves certain risks. Cr contains carcinogenic Cr(VI). The plating process involves Pb, which has the potential to be soaked up through the skin and leads to harm to the liver, organs, and brain. Cyanide is extremely poisonous. It is utilized in the process of Cr plating and has the potential to be lethal to humans. Additionally, Cd utilized during the procedure has the potential to induce cancer as well as issues with kidney and lung functionality. The mitigation of hazards comprises the following measures: Provide enough ventilation. Use proper protecting respiratory equipment. When dealing with Cr plating, wear the required protective clothing and equipment and consistently adhere to safety protocols. Avoid exposure to toxic cyanide.	[505]
		Cr is a significant pollutant found in numerous toxic waste locations globally, such as the Superfund sites in America.	[506]
		Employees in sectors that utilize Cr are more likely to experience the negative health impacts of the element. It is necessary to avoid excessive exposure to Cr(VI) compounds	[507]
	Fe	Fe powder or dust is a solid that can catch fire easily. It can lead to irritation of the eyes and skin by mechanical means. It can result in abnormalities in the blood. It can also lead to harm in the lungs. Breathing in Fe fumes can lead to metal fume fever. It can lead to heart issues and harm the liver. The ingestion of Fe powder can result in gastrointestinal irritation accompanied by symptoms like nausea, vomiting, and diarrhea. Continuous exposure can lead to damage to the pancreas, diabetes, and abnormal heart function. The mitigation of hazards comprises the following measures: Avoid heat, sparks, open flames, and hot surfaces. Smoking is prohibited. Ground/bond the container and receiving devices. Utilize electrical, ventilating, and lighting equipment that is explosion-proof. Use protective gloves/protective attire/eye safety gear/face shields.	[508]
		Excessive intake of Fe by humans can lead to serious symptoms, liver damage, and possibly death. Signs progress via different stages, starting with vomiting, diarrhea, and abdominal pain. Liver failure may manifest itself several days after the initial onset. It is necessary to control Fe levels in the diet, and Fe overdosing is prohibited.	[509]

Most information about hazards accompanying GSR is related to Pt, Rh, Re, Pd, and Cu catalysts and Cr promotors. Surprisingly, although Ni-based catalysts have been reported as the ones most often applied in reforming reactions, little information was found about hazards related to their application.

Silva et al. [444] noticed that the GSR technique can effectively transform C_3_H_8_O_3_ into H_2_, primarily because its scaling up necessitates minimal alterations to existing industrial methods of H_2_ generation from fossil fuels, which predominantly rely on SR. To improve H_2_ production through the GSR process, selecting a suitable catalyst is essential. Ni and precious metals such as Pt and Ru have been extensively researched. Although Ni catalysts are less expensive, noble metals exhibit higher activity and stability, enabling operations at reduced temperatures. However, many efforts have been dedicated to finding Ni-based catalysts that match the performance of noble metal materials. The impact of catalyst supports should also be acknowledged, as neutral supports (e.g., SiO_2_) exhibit greater stability and reduced C deposition. Employing promoters might also be essential to enhance the stability of the catalyst, which is a crucial concern in this area. Therefore, additional studies need to be conducted in this field, particularly focusing on the quest for stable and low-temperature active Ni-based catalysts.

Various mechanisms have been suggested for the GSR reaction (e.g., the Langmuir–Hinshelwood dual-site mechanism involving the molecular adsorption of C_3_H_8_O_3_ and the molecular or dissociative adsorption of H_2_O). Nonetheless, an agreement on this issue has not been achieved; thus, additional research is necessary. Additionally, because the GSR reaction significantly encourages the creation of carbonaceous deposits, the investigation of coke deposition kinetics has been conducted, with the authors proposing that a Langmuir–Hinshelwood single-site mechanism with the dissociative adsorption of C_3_H_8_O_3_ and molecular adsorption of H_2_O can explain the coke deposition on a Co-Ni/Al_2_O_3_ catalyst. Nevertheless, greater attention on this topic is necessary, as well, to reveal the potential presence of various mechanisms of coke deposition on different types of catalysts.

Even when an effective catalyst and advantageous operating conditions are employed in catalytic GSR within a traditional reactor (e.g., fixed-bed reactor), thermodynamic constraints regarding C_3_H_8_O_3_ conversion and H_2_ yield remain. To circumvent these limitations, enhanced processes that merge the GSR reaction with the selective removal of CO_2_ or H_2_ in a single physical device have emerged as a superb alternative. It has been noted that eliminating CO_2_ or H_2_ from the reaction medium moves the thermodynamic equilibrium toward increased C_3_H_8_O_3_ conversions and elevated H_2_ yields. Furthermore, these processes enable operation at reduced temperatures while achieving comparable or even superior performance compared to traditional reactors at elevated temperatures. Nevertheless, for these processes to be effectively implemented, it is essential to utilize CO_2_ sorbents that possess strong sorption capacity, stability, and low sorption and regeneration temperatures (e.g., 300–500 °C), alongside H_2_ perm-selective membranes that demonstrate high H_2_ selectivity and permeability, as well as significant resistance to embrittlement and poisoning. Currently, hydrotalcite-derived CO_2_ sorbents (which need reduced sorption and regeneration temperatures) and Pd-based membranes are regarded as potential systems for Sorption-Enhanced Glycerol Steam Reforming (SEGSR) and GSR in MRs, respectively, at lower temperatures (300–400 °C). Furthermore, suitable operating conditions (temperature, H_2_O/C_3_H_8_O_3_ Feed Ratio (molar) (WGFR), pressure, and Weight Hourly Space Velocity (WHVS)) must be meticulously selected. CO_2_ emissions can be readily mitigated through SEGSR, but MRs utilizing H_2_ perm-selective membranes are ineffective in this regard. 

Carrero et al. [483] noticed in some reports that adding promoters such as Zr, Ce, and La to Ni-based catalysts can alter the size and distribution of metallic particles, thus affecting their catalytic efficiency in ethanol SR. La_2_O_3_ might inhibit metal sintering and reduce coke buildup because oxycarbonate compounds such as La_2_O_2_CO_3_ can interact with C deposits, releasing CO and regenerating La_2_O_3_. CeO_2_ is utilized as a promoter because of its redox characteristics and significant O_2_ mobility, which can diminish C deposits in SR processes [19]. The inclusion of ZrO_2_ in the Ni/Al_2_O_3_ catalyst demonstrated its ability to prevent metal sintering when exposed to H_2_O at elevated temperatures; furthermore, ZrO_2_ enhances steam adsorption, facilitating the transfer of steam from the support to the active metallic sites, which improves the GA of surface hydrocarbons and/or C deposits. Due to the reasons stated above, numerous studies have documented the addition of Ce, Zr, and La to Ni-based catalysts applied in ethanol SR. Nonetheless, the promotional effects of Ce, Zr, and La on Co/SBA-15 samples have been rarely examined. These altered cobalt catalysts have been utilized before in Fischer-Tropsch synthesis, CH_3_OH decomposition, and benzene oxidation, yet there are no citations regarding their application in GSR. 

Carrero et al. [483] examined GSR on Co/SBA-15 and enhanced Co/M/SBA-15 (M: Ce, Zr, La). They found that the addition of promoters such as Zr, Ce, and La on an SBA-15 support, followed by Co impregnation, resulted in smaller Co crystallites, enhancing metal dispersion. In addition, more robust interactions between Co species and M/SBA-15 supports were noted. Due to the inclusion of Zr, La, and primarily Ce, the enhanced catalysts exhibit greater C_3_H_8_O_3_ conversion compared to Co/SBA-15 over 5 h of operation. Moreover, at 600 °C, Co/M/SBA-15 (M: Zr, Ce, or La) catalysts generate greater quantities of H_2_ compared to Co/SBA-15.

Ghasemi et al. [32] stated that further investigation is required to minimize CO_2_ emissions and production expenses in the SR process. Economic challenges to SR involve expenses related to the process and catalysts. The enhanced efficiency and prolonged lifespan of the precious metal catalyst must offset the higher cost of each catalyst to tackle these challenges. Additionally, increasing the size of the plant will lead to greater Capital Expenditures (CAPEX) but significantly reduced H_2_ production expenses. 

### 4.4. Electrochemical Methods

Various methods for transforming biomass into H_2_ through low-temperature electrochemical conversion have been established [511]. H_2_ production through electrochemistry from biomass can be accomplished by electro-reforming oxygenated organic compounds [65].

Zhao et al. [512] have shown that merging green H_2_ production with raw biomass electro-reforming is an effective and scalable approach to resource recovery. H_2_ can be generated electrochemically from various biomass sources, including agricultural crop remains, forest byproducts, organic municipal solid waste, and animal byproducts [513].

#### 4.4.1. Water Electrolysis

As biomass is composed of H_2_O in different percentages, H_2_O can be considered an instance of biomass. Water electrolysis (WE) is a technique for generating H_2_ from biomass at lower temperatures. It is among the most thoroughly studied techniques [282]. The transformation of biomass into H_2_ via low-temperature electrolysis has recently attracted interest as an expanding area of research. WE qualifies as a low-C H_2_ production technique only when most of the electricity powering the electrolyzer is sourced from renewable energy, including hydro, wind, or solar power plants, with related GHG emissions not exceeding approximately 60 g CO_2_eq/kWh [514].

According to [515], there are two techniques for pure H_2_O, including alkaline water electrolysis and solid oxide electrolysis. 

Alkaline water electrolysis, operating at a commercial scale, is a well-recognized technique where the dimensions of the plant and its specific traits dictate the investment expenses for this process. In 2012, the estimated production expense for such a method was 3.48 USD/kg in Germany and 5.56 USD/kg in the UK. Throughout this procedure, asbestos diaphragms and nickel components serve as electrodes. Consequently, additional research is required to substitute harmful asbestos with alternative environmentally friendly materials [515].

Solid oxide electrolysis presents a highly promising technological approach for effective and widespread H_2_ production at a large scale. This method works at temperatures ranging from 500 to 850 °C, using H_2_O as steam. Water splitting is an endothermic reaction, and as the temperature increases, reduced power and voltage are required for dissociation. This method demonstrates great efficacy, lasting stability, low emissions, and an affordable operating cost. The elevated operating temperature is the primary disadvantage, as it leads to longer startup periods and issues with mechanical and chemical compatibility. Additionally, the degradation of electrode polarization resistance presents another challenge at elevated current densities. Investigations must be conducted to reduce these problems in this procedure [515].

During WE (both in alkaline water electrolysis and solid oxide electrolysis), H_2_O is converted to O_2_ and H_2_ at a molar ratio of 1:2 [515].

Excessive O_2_ can reduce human breathing and heart rate to perilous levels. Excessive O_2_ may result in O_2_ toxicity or O_2_ poisoning [516].

Inhaling pure O_2_ under high pressure may lead to symptoms like nausea, dizziness, muscle spasms, vision impairment, convulsions, and unconsciousness [517].

O_2_ can initiate or enhance a fire since it acts as an oxidizing agent.

O_2_ stored in a tank under pressure can explode if heated.

Exposure to quickly expanding gas can lead to burns or frostbite.

The mitigation of hazards comprises the following measures [518]:

- Minimize contact with pure O_2_.
- Stay away from gas that is expanding quickly. Proper protective gear and attire are essential in the event of exposure.
- Attempt to heat the frozen areas and obtain medical assistance.
- Avoid contact with garments and other flammable substances. Ensure that reduction valves, valves, and fittings are kept clean and free of oil and grease.
- If it is safe, stop the leak in the event of a fire.
- Shield storage devices from direct sunlight. Keep in a location with good air circulation.

WE is in the commercialized stage, and its cost is 10.3 USD/kg. The process drawback is high capital cost, where the electricity cost is about 80%. A remedy for this is electricity generation from renewable sources. The process efficiency is in the range of 60–80% [519,520,521].

Also, according to [67], biomass can undergo electrochemical conversion. However, the anode reaction is what sets WE apart from biomass electrolysis. Rather than producing gaseous O_2_ from H_2_O, the raw material is oxidized. MECs and proton exchange membrane electrolysis cells (PEMECs) are two methods that can be employed to electrolyze biomass [67].

For bio-derived substances like ethanol and C_3_H_8_O_3_, both proton exchange membrane electrolysis cells (PEMECs) and microbial electrolysis cells (MECs) are commonly employed. Electrolysis cannot directly convert polymeric materials like cellulose or wood sawdust. These systems require one or two chambers with an anode/cathode connection. At the anode, organic material undergoes an oxidation process that produces protons (H^+^). At the cathode, a reduction reaction takes place, allowing H_2_ generation [67]. 

#### 4.4.2. Microbial Electrolysis Cells

Microbial electrolysis cells (MECs) consist of an anode and cathode, separated by an ion exchange membrane. In comparison to other electrolysis techniques, it requires significantly less energy and generates a high H_2_ yield [515].

This method merely involves the generation of H_2_ via the catalytic activity of microorganisms. Active bacteria facilitate the liberation of protons, electrons, and CO_2_ from organic substances throughout this process [193]. This technology operates within microbial electrolysis cells (MECs). By utilizing this technology, the soluble organic substances in waste H_2_O can be converted into storable chemical energy, such as H_2_ [522]. Microbial electrolysis cells (MECs) utilize a low extra electric current to generate H_2_, alongside the energy and protons that microbes release when they decompose organic substances [513]. Exo-electrogenic microbes are capable of converting basic acetate and glucose into pure H_2_ gas. To surpass the thermodynamic obstacle of the electrolysis process, an external voltage needs to be applied to the electrolysis cell. Furthermore, the rate of H_2_ production continues to be restricted [523]. Improving the rates and outputs of H_2_ production from fermentation processes by employing different methods, including upgrading microbial strains, optimizing reactor systems, and identifying the most advantageous types of microorganisms to use, are significant fields of research and development [513]. Liu et al. [524] tackled the issue of enhancing rates and yields of H_2_ production from fermentation processes using several methods, including the optimization of reactor systems by incorporating a dynamic membrane into the anode electrode of the microbial electrochemical system (MES). In contrast to systems lacking dynamic membrane filtration, the incorporation of the dynamic membrane led to enhanced current densities, reduced effluent turbidity, better treatment efficiency, and energy production.

Microbial electrolysis can be accompanied by Cl generation. However, due to the anode’s capacity to generate Cl at an extremely low potential, there is no need to isolate the electrolyte from the Cl, which makes this process more energy-efficient compared to other H_2_O electrolysis techniques. Additionally, this procedure enhances safety since the pH level remains neutral and O_2_ production does not occur [525].

Cl is a non-flammable gas; in contact with H_2_O, it forms a HCl solution. Contact with Cl can cause severe irritation and burns of the skin and eyes and irritate the nose and throat. Inhaling it can irritate the lungs. Contact with liquid Cl can induce frostbite. Cl can trigger an allergy similar to asthma. Being exposed to Cl may result in headaches, dizziness, nausea, and vomiting. Continuous exposure might result in lasting lung harm [526].

The mitigation of hazards comprises the following measures [526]:

- Avoiding areas with increased Cl concentration.
- Controlling the Cl concentration in the air.
- Ensuring the adequate ventilation of rooms.
- Wearing appropriate protective clothing, gloves, respiratory masks, and goggles, especially in areas with increased Cl concentration.

Pt-, Ni-, and C-based catalysts are applied in microbial electrolysis units to enhance reaction kinetics, H_2_ production, and cleanliness [515].

Microbial electrolysis is in the developing stage. This process has an efficiency of 78 % [275]. 

#### 4.4.3. Proton Exchange Membrane Electrolysis Cells

In the realm of proton exchange membrane water electrolysis cells (PEMECs), a durable polysulfonated membrane is utilized. The membrane electrode assembly is an essential part of the cell, comprising a cathode, an anode, and a membrane. Nafion^®^ membranes are the most commonly utilized ones due to their mechanical strength, proton conductivity, and suitable gas permeability [515].

The H_2_ evolution reaction takes place at the cathode, whereas the O_2_ evolution reaction happens at the anode. Electrocatalysts for H_2_ evolution reactions are typically composed of Pt or Pt-based catalysts, whereas electrocatalysts for O_2_ evolution reactions are generally made of catalysts based on Ir or Ru. This method is regarded as the most favorable approach for generating clean H_2_, as a 1 MW PEM H_2_O electrolyzer can yield up to 6 m^3^/h of H_2_. Among its advantages are high efficiency, low operating temperatures, affordable maintenance expenses, high current density, straightforward design, and reduced gas permeability [515].

Nevertheless, due to the membranes’ reduced efficiency at elevated temperatures and their limited longevity, this method incurs significant catalyst expenses. Consequently, it is essential to identify membranes that are cost-effective and possess enhanced longevity by utilizing materials suitable for ongoing use [515].

Various catalysts can support various electrolysis techniques. Pt and Ir catalysts are utilized in proton exchange membrane (PEM) electrolysis to improve H_2_ production at the cathode and O_2_ generation at the anode reactions. Pt-based catalysts can enhance the efficiency of H_2_ production to 90.8% in PEMs. Catalysts reduce the energy needed to break H_2_O apart, thus enhancing the efficiency of H_2_ production. Ni-Mo-, Ni-Co-, and Fe-based catalysts are utilized in the electrolysis of alkaline H_2_O. Perovskite-based catalysts are currently under development for this approach, which provides excellent stability and performance. Ni- and perovskite-based catalysts are likewise utilized in solid oxide electrolysis, decreasing energy consumption and enhancing longevity. Co catalysts diminish the operating temperature ranges from 800 °C down to 500–600 °C in solid oxide electrolysis. These are also economical in comparison to the catalyst needed in the PEM procedure [515].

#### 4.4.4. Projects on Electrochemical Techniques

Since biomass components can be transformed into H_2_ gas via electrochemical cells, choosing the right electrode materials is essential because they directly interact with the biomass-derived fuel and improve electrochemical reactions. Catalysts like Pt, Ni, or other metal compounds can enhance the speed and effectiveness of H_2_ evolution reactions. The kind and strength of an electrolyte solution influence electrochemical performance, impacting ion conductivity, reaction rates, and the stability of electrodes. Maintaining an optimal pH level is essential for maximizing the efficiency of bio-H_2_ production. A new chemical electrolytic conversion (CEC) method was created utilizing aqueous polyoxometalate (POM) as a catalyst. This method facilitates the direct release of H_2_ at low temperatures from natural biomass sources like cellulose, lignin, wood, and grass powder. The unprocessed biomass undergoes oxidation, and electrons move to POM molecules via heat or light exposure. Protons from biomass move to the cathode and get reduced to H_2_. The energy usage is merely 0.69 kWh per cubic meter of H_2_ at 0.2 A cm^−2^ [527]. Considering sustainability, this technology is highly energy-efficient as it operates at low temperatures, allows for various biomass sources, and requires minimal energy consumption. The selected catalyst, polyoxometalate in its H_2_O-soluble form, is an effective catalyst that can be investigated for additional methods of H_2_ production [265].

Another study investigated the GA of biomass in a fluidized-bed reactor utilizing Fe/CaO catalysts. The catalysts were created with different mass ratios to enhance the concentrations and yields of syngas. The Fe load notably influenced the composition, textural characteristics, and CO_2_ absorption abilities. The peak syngas production was obtained with an optimized mass ratio of Fe/CaO at 5%, 26.40 mol/kg of biomass, an 8.69 MJ/kg LHV, and a gasification efficiency of 49.15%. The characterization results also showed the emergence of a Ca_2_Fe_2_O_5_ phase, which was anticipated to enhance tar cracking by inhibiting CaO deactivation [528]. Nevertheless, this stage did not influence gasification and led to a decreased ability to absorb CO_2_. The gasification process examined in this study is not particularly efficient, although the Ca_2_Fe_2_O_5_ phase could effectively minimize undesired byproducts through tar cracking [183]. 

Hibino et al. [529] carried out a study to decrease energy usage by directly electrolyzing bread, sawdust, and rice husk at 150 °C. They produced H_2_ at the anode, designed without precious metals and featuring O_2_-functionalizing C. The catalytic activity was similar to that of conventional Pt/C anodes. Upon the addition of PO_4_, waste biomass underwent hydrolysis, enhancing electrolysis. The average yield was 0.25 mg per mg of feedstock. This indicates that traditional noble metal anodes might be substituted with alternative anodes that can be self-produced, as commonly used noble metals such as platinum are costly. The constraints on the non-noble metal utilized could be mitigated by adding phosphoric acid to hydrolyze the biomass waste. The significant H_2_ output and economical approach suggest the feasibility of this process pathway.

## 5. Hazards Related to Separation and Purification of Hydrogen

Numerous research investigations have been conducted on H_2_ purification systems and continue to take place [530,531,532]. H_2_ purification methods can be physical, such as adsorption and membrane separation, as well as chemical, including catalysis and metal hydride separation [533]. Certain research indicated that absorption is a favored method because it operates at low temperatures and pressure [66,534]. Nonetheless, the expense associated with the purification process continues to be a challenge that needs to be tackled to utilize affordable membrane materials, for example. Research continues, for instance, with new adsorption materials and non-toxic metal hydrides to ensure cost-effectiveness and enhance application tolerance for H_2_ purification.

Shahbaz et al. [535] discussed biomass processing and conversion methods for H_2_ generation and analyzed membrane H_2_ separation systems. They reported that membranes, absorption, and adsorption such as pressure swing adsorption (PSA), temperature swing adsorption (TSA), electrical swing adsorption (ESA), and cryogenic technologies are often applied at an industrial scale for the separation and purification of H_2_ extracted from biomass.

Amin et al. [536] analyzed the problems and difficulties in separation technologies. Four key technologies, namely, membranes, adsorption processes, metal hydride (MH), and cryogenic processes, were analyzed and examined. 

The membrane technology comprised polymeric, mixed-matrix membranes (MMMs), ceramic, zeolite, metallic, and carbon membranes. High performance requires an exceptional blend of permeability and selectivity. Additionally, stronger membranes that are thermally, chemically, and mechanically stable are needed for industrial separation processes. Therefore, polymeric membranes are not the ideal selection based on this criterion because of their inadequate thermal stability. MMMs face challenges with interfacial defects and filler agglomeration that must be resolved before their commercialization. The high production cost of ceramic membranes restricts their use on a commercial level. In the manufacturing of metal membranes, there is an issue related to the reduction in their permeability resulting from the elevated solubility of contaminants. The optimization of the metallic membrane (such as microstructure, heat treatment, and activation techniques) is a present concern that must be considered. Zeolite membranes, like carbon membranes, are fragile and challenging to manufacture. In every kind of membrane, the presence of impurities and multicomponent gas mixtures negatively impacts membrane efficiency, representing a significant constraint on membrane technology for purification [536]. 

Likewise, the adsorbents employed in adsorption processes possess a greater attraction for impurities (CO_2_, H_2_O, NH_3_, and light and heavy hydrocarbons) and can eliminate nearly all of them. Nonetheless, the primary difficulty lies in the elimination of CO and inert gases like N_2_ and Ar because of the limited adsorption ability of traditional adsorbents (zeolites and activated carbons). TSA is viewed as a promising method compared to PSA and VSA for enhancing recovery and purity; however, it requires more energy owing to the extra expense of the heating and cooling processes. In the MH process, it is essential to create catalyst-free systems to lower the expenses involved. Moreover, it demands significant heat transfer that diminishes the system’s efficiency. The cryogenic method is a well-established technology that has been functioning for numerous decades. It also includes challenges that require improvement, such as high capital costs and a low H_2_ purity of 95–98% [536]. 

Metal hydride reactors, utilizing various metal alloys, play a crucial role in the absorption and desorption processes for purifying H_2_ from contaminants, essential for maximizing reaction efficiency and attaining high purity levels. The requirements for heat and mass transfer are crucial factors to consider when designing a metal hydride reactor. The separation relies on various factors, such as the reaction kinetics, temperature, pressure of the hydride bed, enthalpy, and cycle time, among others. To address the challenge related to reaction kinetics, incorporating amorphous Ni-Li-B catalysts enhances the reaction kinetics of Al-based alloys [536].

The cryogenic technique is eco-friendly since it avoids chemicals, thus eliminating the generation of secondary pollutants. A significant disadvantage of this approach is its large capital and operational expenses [533,536].

## 6. Techno-Economic and Environmental Aspects of Renewable-H_2_ Production Technologies

The evaluation of various technologies used for renewable-H_2_ extraction from biomass necessitates the consideration of techno-economic aspects such as capital costs, H_2_ costs, production efficiencies, and technology readiness levels (TRLs) presented in Table 10 and of environmental impacts. For the latter, the Life Cycle Assessment (LCA) methodology for assessing the environmental effects of a product throughout its entire life cycle is commonly used.

Buffi et al. [537] evaluated various methods of H_2_ generation from biomass, including thermochemical ones (such as PY, liquefaction, and GA) and biological ones (including DbP and i-DbP, the biological WGS reaction, as well as PF and DF). They found that although certain pathways exhibit low TRLs, others, such as steam bio-CH_4_ and biomass GA, are ready for immediate market adoption.

**Table 10:** Comparison of different H_2_ production technologies.

Process	Feedstock	Capital Costs (M-EUR)	H_2_ Cost (EUR/kg)	Efficiency (%)	TRL	Ref.
Biomass PY	Biomass + Heat + Steam	53.4–3.1	1.3–2.2	17–33	4–5	[538]
Biomass GA	Biomass + H_2_O	149.3–6.4	1.8–2.1	35–50	7–8	[539]
HT GA	Biomass + Heat + Steam	-	1.5–3.2	70	2–3	[540]
DbP	Sun + H_2_O + Algae	50 USD/m^2^	2.13	12.2	2–3	[541]
i-DbP	H_2_O + Algae	135 USD/m^2^	1.42	4.1	2–3	
DF	Biomass + Anaerobic bacteria	-	2.57	12	4–5	
PF	Sunlight + Biomass	-	2.83	8.5	4–5	
Electrolysis	H_2_O + Electricity	-	10.3	60–80	9	[542]
MEC	Waste H_2_O + Electricity	-	-	67–90	<5	[542]
PEMEC	Waste H_2_O + Electricity	-	-	70–80	7–8 (9)	[542]

### 6.1. Techno-Economical Aspects of Renewable-H_2_ Production Technologies

The cost of renewable H_2_ is linked to the technology used for production. In 2021, the levelized cost of H_2_ (LCOH) produced via NG SR was between 0.92 and 2.8 EUR/kg H_2_ [543]. According to [543], this method remains the most cost-effective way to produce H_2_ in many areas globally. The costs of producing green H_2_ are also connected to the renewable energy source. The estimated LCOH from CH_4_ SR in the Eurozone for 2022 was 5.7 EUR/kg H_2_. Various techno-economical aspects of renewable-H_2_ production technologies, including biological ones and thermochemical ones, have been evaluated in several studies. Hosseinzadeh et al. [544] performed a techno-economic and environmental impact evaluation of key H_2_ production methods, including dark fermentation, photo-fermentation, solid-state fermentation, microbial electrolysis cells (MECs), gasification, pyrolysis, and plasma processes. From a technological perspective, DF has demonstrated superior performance to other methods. Nonetheless, the combination of DF with PF and MECs has demonstrated superior performance, yielding approximately 1 L H_2_/g organic waste. In terms of the economic factor, the least expensive H_2_ production methods are GA and fermentation, costing roughly 2 USD/g and 2.3 USD/g, respectively, followed by plasma at 2.4 USD/g, PY at 2.6 USD/g, MECs at 2.8 USD/g, and PF at 3.5 USD/g. Regarding possible environmental effects, the fermentation method exhibited the least GHG output, measuring 15 kg CO_2_-eq/kg H_2_, followed by GA, MECs, and plasma. In terms of possible commercial uses, GA stands out as the most advanced, boasting the highest attainable technology readiness at level 9.

#### 6.1.1. Biological Technologies

Han et al. [545] conducted a techno-economic evaluation of a H_2_ production method integrating solid-state fermentation with DF. They confirmed the economic viability of the method with a H_2_ production cost of 2.1 EUR/m^3^ H_2_, achieving a payback period of 5 years and an internal rate of return (IRR) of 20.2%. 

Hosseinzadeh et al. [544] evaluated the mean H_2_ production from a DF pilot facility with a processing capability of 8-45 g H_2_/kg biomass, calculating it at 2.1 EUR/kg H_2_. 

Alam and Nayan [546] introduced a simulation model for renewable-H_2_ production through the DF of waste-H_2_O sludge. Their research indicated that H_2_ can be generated from DF for 10.5 EUR/kg H_2_ for a biomass processing capacity of 23 t/day, which might be significantly reduced to 5.4 EUR/kg H_2_ by increasing the capacity to 500 t/day.

Anaerobic digestion (AD) for generating BG/bio-CH_4_ can facilitate the production of renewable H_2_ via the reforming of the resultant BG/bio-CH_4_. Szima et al. [547] calculated an LCOH of 0.15 EUR/Nm^3^ for a flexible H_2_ and electricity co-generation facility utilizing the dry reforming of bio-CH_4_ with minimal CO_2_ emissions.

A related technological approach to the joint generation of H_2_ and electricity was examined by Cormos et al. [548], confirming an LCOH expense of 58 EUR/MWh. The authors determined that capital expenses significantly affect LCOH, with BG expenses and plant availability factors following, whereas operating expenses have a lesser effect.

Hajizadeh et al. [549] investigated H_2_ generation from cow dung by combining psychrophilic AD and dry CH_4_ reforming. The procedure was refined to achieve the maximum CH_4_ conversion and the minimum energy usage. The authors’ economic analysis revealed that the cost of H_2_ production is influenced by the rate of H_2_ production, where increased production rates lead to reduced production costs. The optimal scenario was identified for a plant capacity of 45.5 kg/h H_2_, resulting in a H_2_ production expense of 1.28 EUR/kg H_2_. 

Byun and Han [550] assessed the financial feasibility of employing the AD of food waste combined with bio-CH_4_ SR to produce renewable H2. The primary cost factor of the biodigester is its capital expense. This cohesive approach enabled a H_2_ minimum selling price (MSP) of 24.2 EUR/kg H_2_, influenced by the plant’s capacity. In the optimal scenario of a 2000 t H_2_/d processing capacity, H_2_ could be produced at an MSP of 5.5 EUR/kg H_2_, which is on par with fossil-based H_2_ production methods.

#### 6.1.2. Thermochemical Technologies

The economic aspects of different biomass-to-biofuel scenarios were analyzed by Anex et al. [551], who concluded that H_2_ generated through biomass PY possesses the lowest production expense owing to its minimal capital operating cost of 0.53 EUR/kg H_2_.

Brown et al. [552] analyzed the techno-economics of fast PY of corn stover for H_2_ production and found that the costs of H_2_ production (1.9–2.8 EUR/kg H_2_) were higher than those in earlier studies.

Tan et al. [553] reported that H_2_ from biomass PY had a cost of 1.64 EUR/kg H_2_. 

Lepage et al. [282] assessed the economic viability of various biomass-derived methods for H_2_ generation. As stated by these authors, conventional SR of NG seems to be the least expensive choice, with H_2_ production costs under 0.92 EUR/kg H_2_, while biomass PY could result in renewable-H_2_ production costs ranging from 1.17 to 2.37 EUR/kg H_2_. Generally, elevated H_2_ production costs (1.1–3.2 EUR/kg H_2_) occur. 

Li et al. [554] examined the generation of H_2_ and biochar through the pyrolysis of corn straw. The writers indicated that expenses associated with biomass feedstock were the greatest (68%), followed by the costs of catalysts (12%). Employee wages and benefits made up 8% of the overall expenses, whereas electricity constituted 4% of the total costs. With an annual processing capacity of 40,000 tons of corn straw, a potential maximum profit of EUR 805 million and an IRR of 22% over 7.3 years were projected.

Salkuyeh et al. [555] examined the economic factors related to H_2_ production from biomass GA using two reactor setups: a fluidized bed (FB) and an entrained flow (EF). The authors additionally examined the impact of introducing C capture and liquefaction systems on the expenses associated with H_2_ production. FB gasification technology enabled a more economical process for H_2_ production, achieving a H_2_ MSP of 2.8 EUR/kg H_2_. The elevated H_2_ cost for the EF process (3.1 EUR/kg H_2_) resulted mainly from the increased CAPEX associated with this gasifier type. The incorporation of carbon capture and liquefaction systems caused H_2_ production expenses to rise by 3% and 11% for the FB and EF processes, respectively.

Shaikh et al. [556] employed an Aspen Plus simulation to evaluate the TEA of a biomass Ca looping GA combined cycle for the simultaneous production of H_2_ and electricity. The estimated cost of producing H_2_ was 2.2 EUR/kg H_2_, with an IRR of 17.43% and a payback duration of 7.35 years. The integrated H_2_–H_2_-electricity method seemed to be more financially viable than generating electricity.

Additional studies also examined the techno-economic viability of H_2_ produced from biomass gasification. For example, Wang et al. [557] indicated production cost rates of 0.9 EUR/kg H_2_. 

Ellofy et al. [67] estimated that a common route for biomass GA and SR and/or WGS with a pressure swing adsorption system (PSA) needs 2.4 TJ of primary energy input per TJ of H_2_, and for a plant with a H_2_ output of 139–700 kg/day and a biomass cost of USD 46–80 /dry-ton, the H_2_ generation cost is in the range of USD 1.77–2.05 /kg.

Al-Qahtani et al. [558] analyzed the economic aspects of H_2_ generation from biomass GA, assessing the impact of C capture and storage (CCS) technology both in its presence and absence. The authors discovered that incorporating CCS led to a rise in LCOH from 2.2 to 3.4 EUR/kg H_2_.

Concerning HT processes, the financial assessment of H_2_ generated from the HTG of soybean straw found an MSP of 1.79 EUR/kg H_2_ [559].

Cook and Hagen [560] evaluated the economic aspects of three case studies (plants) for H_2_ production through biomass GA in the United States. The costs of biomass supply and the distance for H_2_ transportation were identified as the primary factors influencing total production expenses. The optimal situation indicated a minimum H_2_ transportation cost of 3.19 EUR/kg H_2_, potentially rising to 3.78 EUR/kg H_2_ in a less advantageous scenario. 

#### 6.1.3. Electrochemical Technologies

Tanyi et al. [561] evaluated the potential to develop a green H_2_ market in Ghana. The evaluation was focused on biomass GA and photovoltaic-driven WE. The authors estimated that distributed and centralized polymer electrolyte membrane (PEM) electrolysis gave levelized costs of USD 5.56/kg and USD 4.35/kg for H_2_, respectively, while centralized biomass gasification yields the cheapest H_2_ cost at USD 2.68/kg.

Shin et al. [562] examined, in an economic assessment, the combinations of three renewable power plant types, including offshore wind, onshore wind, and onshore photovoltaics, alongside two H_2_O electrolysis types: alkaline water electrolysis (AWE) and polymer electrolyte membrane water electrolysis (PEMWE). The LCOH calculation revealed that the onshore wind-power–AWE scenario presents the lowest LCOH at 7.25 USD/kg, whereas the PV-PEMWE scenario shows the highest LCOH at 13.44 USD/kg.

Vives et al. [563] examined the techno-economic viability of recovering waste heat from multi-MW-scale green hydrogen generation. A 10 MW proton exchange membrane electrolysis system was designed with a heat recovery mechanism integrated with an organic Rankine cycle (ORC) to facilitate the mechanical compression of hydrogen. The technical outcomes show that by utilizing waste heat recovery alongside an ORC, the first-law efficiency of the electrolyzer rises from 71.4% to 98%. The ORC can produce enough energy to compress hydrogen from the electrolyzer’s outlet pressure of 30 bars to 200 bars. A financial analysis was performed to determine the levelized cost of hydrogen (LCOH) of the system and evaluate the practicality of integrating the waste heat recovery with the ORC. The findings indicate that electricity costs are predominant in the LCOH. When electricity costs are low (e.g., dedicated offshore wind energy), the LCOH increases when applying heat recovery. The extra capital and operating expenses related to the ORC raise the LCOH, and these extra costs surpass the savings from eliminating electricity purchases for compression. Conversely, heat recovery and the ORC become appealing and viable when the prices of grid electricity rise.

### 6.2. Barrier to Commercialization of Renewable-H_2_ Production Technologies

The H_2_ economy faces several obstacles to its progress and successful commercialization. Recent information validates the enhanced efficiency, cost-effectiveness, and scalability of technologies for producing green H_2_. Additionally, the cost of green H_2_ has decreased significantly [564]. However, according to [565], the global expansion of such an economy faces multiple significant obstacles, such as the lack of a value chain for clean H_2_, challenges in H_2_ storage and transport, expensive production, the absence of international standards, and investment risks. H_2_ generated on a large scale via different methods faces specific techno-economic challenges. Table 11 (based on [32]) presents the obstacles of various H_2_ conversion TH methods limiting their commercialization. Table 12 (based on [32]) shows such obstacles for biological methods.

#### 6.2.1. Technical Challenges

Barriers to the commercialization of H_2_ production include lower efficiency and increased costs relative to alternative methods [566]. The production of H_2_ via fermentation is steadily rising from its early phases as technology advances [567]. To boost the quantity and speed of H_2_ production, various strategies should be employed at both the genetic and fermentation stages. Through genetic engineering, strains specifically created for H_2_ production might be developed. The incorporation of H_2_ production processes enhances the overall efficiency of the entire procedure.

Inoculum pretreatments and increased energy use hinder the commercialization of the DF process. Before DF is brought to market, it is essential to research economic and technical viability to avert significant process hindrances [568]. The drawback of DF is its ability to produce only 4 mol H_2_/mol of glucose, which continues to pose a major challenge [201]. To ensure that a H_2_ process is commercially feasible, it is essential to enhance the complete process.

The main issues in thermochemical conversion for H_2_ production revolve around handling the raw materials and making them ready for subsequent processing. Table 11 illustrates the technological and financial challenges associated with the practical application of H_2_ TH conversion methods. The main objective of low-temperature GA systems is to produce tar through PY and GA.

The main obstacle in the commercial GA process is due to organic impurities in the produced syngas, resulting in considerable operational difficulties [569]. Moreover, external conditions and the requirement to sustain ideal temperatures within the gasifier, along with the efficient extraction of tar, present significant challenges. Additional challenges comprise inefficient feed delivery, excess metabolites present in reactors, and the reduced conversion efficiency of substrates when dealing with mixed organic waste. These difficulties arise either from the complex characteristics of the substrates or from the lack of microbial communities that can efficiently hydrolyze them [570].

**Table 11:** Obstacles of various H_2_ conversion TH methods limiting their commercialization. Based on [32].

Thermochemical Conversion	Technical Obstacles	Financial Obstacles	Potential Strategies for Overcoming These Barriers	Ref.
GA	Energy usage	Expense of CO_2_		[571]
SR	-	-	The improved efficiency and longer life of a precious metal catalyst balance the higher cost per catalyst unit experienced in the catalytic process.	[572]
GA	Problems like corrosion, fouling, and catalyst deactivation, along with the absence of broad industrial acceptance and standardization of the product, can impede the effectiveness of catalyst applications.	The requirement for high temperatures leads to substantial capital and operational costs when executing certain procedures.	Membrane reactors can improve the efficiency of thermochemical processes by using different H_2_-production methods.	[573]
SCWG	-	The viability of a project depends on the financial factors related to obtaining algal biomass and the yield produced.	Optimization plays a vital role in research to improve fuel production. When a payment is made from a carbon dioxide emitter to an algal conversion plant, the expense of H_2_ reduces.	[413]

#### 6.2.2. Financial Hurdles

The DF process is notably costly in biological H_2_ production, and much of the research takes place in laboratory settings. Table 12 emphasizes the challenges associated with both the technical and economic facets of various biochemical conversion methods for H_2_, especially regarding their advancement toward commercial feasibility. Addressing economic obstacles tied to creating affordable photobioreactors and enhancing photosynthesis in DbP and i-DbP are essential goals. A research investigation explored bio-H_2_ generation using an anaerobic membrane bioreactor, identifying that the main economic challenges are related to the high operational and installation costs, which in turn lead to reduced H_2_ outputs [574]. The economic assessment of photobiological H_2_ production is largely conjectural due to the inherently expensive nature of biological H_2_-production methods [575]. The rate of H_2_ production in photobiological processes is quite low, rendering it inappropriate for widespread applications. The total costs for H_2_ production via DF and PF methods were estimated to be between 2.5 and 2.8 USD/kg [180]. A study conducted by Sharma and Kaushik in 2017 indicated that USD 3.70 and USD 18.70 were spent on H_2_ production in DF and PF, respectively. DF H_2_ production is less economically feasible than NG reforming due to its higher costs [567].

Challenges in TC conversion processes arise from PSA (pressure swing adsorption) and expensive catalysts, resulting in higher costs for H_2_ production. While the purification of H_2_ adds extra costs to the process, it simultaneously lowers biomass expenses and enhances efficiency, resulting in a reduction in the total production costs for H_2_ [571].

**Table 12:** Technical and financial obstacles for various biological processes for H_2_ conversion limiting their commercialization. Based on [32].

Biochemical Process	Technical Obstacles	Financial Obstacles	Potential Strategies for Overcoming Such Barriers	Ref.
DF	Establishing, constructing, operating, and managing an appropriate bioreactor.	The primary factor impacting the expense of bio-H_2_ is the cost of substrates.	Feedback inhibition decreases when dark fermentation and photo-fermentation are integrated.	[566]
DF	Because pretreatment methods differ based on the feedstock, conducting pretreatment before fermentation poses a major challenge.	Expensive process.	Extensive, high-level research to overcome financial and technological challenges.	[576]
AD	Variations in H_2_ yield result from differences in biomass, process inhibition, bacteria that utilize H_2_, high levels of heavy metal ions, optimization challenges, and H_2_ storage issues.	Cost of H_2_ storing as a liquid.	The effectiveness of H_2_ production can be enhanced by incorporating chemical additives.	[577]
DF	Thermodynamic limitations exist on the quantity of H_2_ generated through microbial fermentation, in addition to the design and operation of active bioreactors. The primary technological obstacle to the application of DF in practice is its restricted H_2_ production of 4 mol H_2_/mol of glucose.	Elevated expenses associated with the raw materials.	The extraction of energy from the substrate is enhanced when DF is combined with other energy-producing systems.	[269]
The process of integrated DF and PF techniques	A key challenge in the pretreatment process is the presence of inhibitory chemicals. The substrate restricts one or both of the processes.	Due to the harmful nature of waste-H_2_O treatment effluents, the costs of processing increase. The expenses of the procedure are raised in a sequential reactor due to the reactor’s operation and upkeep. The treatment of DF waste H_2_O leads to a rise in operating expenses.	Selecting appropriate H_2_ producers enhances the efficiency of H_2_ production through genetic or metabolic engineering in the integrated DF and PF process.	[578]
PF	-	Increased production at a higher energy expense.	The notable progress in the bio-H_2_ process can be counterbalanced by metabolic engineering. By investigating the effects of nutrient restriction and substrate use, scientists discovered the chromosomal genes in microalgae that play a role in boosting H_2_ production. Advancements in photobioreactor design should be performed with maximum efficacy.	[579]

### 6.3. Environmental Impacts of Renewable-H_2_ Production Technologies

Using the Life Cycle Assessment (LCA) methodology assessing the environmental effect of a product throughout its entire life cycle, an ICCT White Paper [580] assessed the GHG intensity of eight H_2_ production methods, encompassing biological processes using different feedstocks, along with TC and electrochemical approaches. The research emphasizes that H_2_ generated from the GA of forestry biomass and H_2_O electrolysis using renewable energy leads to reduced GHG emissions. On the other hand, H_2_ obtained from the reforming of bio-CH_4_, which comes from the AD of waste-H_2_O sludge or manure, can greatly lower GHG emissions, contingent on CH_4_ leaks throughout the bio-CH_4_ creation process. As anticipated, H_2_ derived from fossil fuels exhibits the greatest GHG intensities among the examined technological pathways, with values surpassing those of the fossil reference.

Camacho et al. [581] conducted an exhaustive LCA of a joint DF/AD waste H_2_O method for producing H_2_. The researchers investigated various feedstocks, such as wine molasses combined with wastewater treatment plant (WWTP) sludge, cheese whey, and sugar beet molasses. To establish the system boundaries, cradle-to-gate and gate-to-gate methodologies were utilized, and the subsequent impact categories were examined: CC—climate change; FE—fresh H_2_O eutrophication; FRS—scarcity of fossil resources; LU—land use; ME—marine eutrophication; TA—terrestrial acidification; TE—terrestrial environment toxicity; and WC—H_2_O usage. The ecological effect was influenced by the feedstock employed to produce H_2_. In the cradle-to-gate method and a Midpoint analysis, sugar beet molasses showed the most favorable environmental profile among the feedstocks evaluated, having a reduced impact in four of the eight categories examined. In comparison, cheese whey demonstrated the poorest relative environmental profile. H_2_ derived from wine molasses and WWTP sludge (9.13 kg CO_2_/Nm^3^ H_2_) and sugar beet molasses (3.56 kg CO_2_/Nm^3^ H_2_) resulted in a smaller C footprint compared to H_2_ from fossil fuels (12.08 kg CO_2_/Nm^3^ H_2_).

Barghash et al. [582] highlighted the advantages of utilizing solar energy to lessen the environmental impact of H_2_ production via DF. The GHG emissions for the procedure conducted with solar energy and without it were −1.12 × 10^4^ kg CO_2_-eq and 3.13 × 10^4^ kg CO_2_-eq, respectively. 

Zheng et al. [583] employed LCA to examine the ecological effects of generating H_2_ via the fermentation of food waste. Electricity, H_2_ compression, and the transportation of food waste were the primary environmental factors. The H_2_ production method examined demonstrated reduced GHG emissions (10.1 kg CO_2_-eq) in comparison to traditional CH_4_ SR (10.6 kg CO_2_-eq) and H_2_O electrolysis (28.4 kg CO_2_-eq). The C footprint was greater compared to the gasification of poplar residues (1.5 kg CO_2_-eq). The LCA for biomass GA does not consider the emissions related to H_2_ compression. 

Ma et al. [584] confirmed the beneficial impact of integrating CCS technologies with H_2_ production from gasifying corn straw, potentially resulting in negative GHG emissions.

Chen et al. [585] utilized LCA to assess the environmental efficiency of the solar-assisted hydrothermal GA of biomass in a H_2_ production pilot plant. The primary environmental contributor was the system operation, while the design of the solar concentrator accounted for 78% of the total global warming potential (GWP). Utilizing solar energy for heating rather than electricity led to a 90% decrease in environmental impact. 

The environmental impact of biomass GA integrated with chemical looping for H_2_ production was assessed by Wu et al. [586]. Such a combined procedure was a viable solution to reduce GHG emissions associated with renewable-H_2_ production, supported by their negative GPW of −15.13 and −17.00 kg CO_2_-eq when employing air and O_2_ as gasifying agents, respectively.

Cormos et al. [587] assessed the generation of decarbonized green H_2_ using Ca-based sorption-enhanced biomass (sawdust) gasification. They discovered that the production of decarbonized green H_2_ through sorption-enhanced biomass gasification exhibits promising results, such as high overall energy efficiency (around 50%), minimized energy and economic penalties for nearly complete decarbonization (up to 8 net efficiency points), low specific carbon emissions at the system level (below 7 kg/MWh), and negative CO_2_ emissions for the entire biomass value chain (approximately −518.40 kg/MWh).

Biomass-based raw materials are often used to produce bio-H_2_ using DF and PF microorganisms. However, they often require purification, and while the impact of individual components present in a combination on the achieved process efficiency is at least initially recognized, the impact of the entire combination is not. Therefore, further studies are needed on this topic [588].

One of the substrates that was exanimated by Polish researchers to produce bio-H_2_ was tannery shavings, which were converted via DF and are giving promising results [589,590,591]. Other valorization processes like biomass torrefaction under superheated steam combined with the GA process and, for example, coupled with pressurized swing adsorption (PSA) to produce green H_2_ are the processes currently examined by researchers [592,593,594,595,596]. 

In summary, these investigations highlight the significance of choosing feedstocks, energy sources, and technological integration for minimizing the environmental effects of H_2_ production from biomass. Biological techniques, such as DF, differ considerably depending on the feedstock, with certain ones exhibiting reduced impacts. Thermochemical techniques, including biomass GA, significantly benefit from advancements like C capture and solar support, capable of lowering or eliminating GHG emissions. Enhancing these elements is essential for reducing the environmental impact of renewable-H_2_ generation. 

## 7. Summary

Evaluating various biomass-derived H_2_ production methods is challenging, primarily due to the significant variation in technologies, feedstocks, operational conditions, scale, and maturity levels, along with associated risks. The hydraulic retention time (HRT), pH, temperature, and eventual substrate pretreatment all significantly influence the optimal route for generating bio-H_2_.

Both TC methods, including PY, liquefaction, and GA, along with biological methods, can generate H_2_ from biomass (i.e., DbP or i-DbP, as well as PF and DF). Among the most promising biomass-to-H_2_ conversion pathways identified, biological methods exhibited superior environmental performance relative to TC methods. The predominant methods for biological H_2_ production involve DF and PH carried out by strict anaerobes, facultative anaerobes, microalgae, cyanobacteria, and bacteria. 

The microorganisms and reactor materials used during bio-photolysis exhibit no or little toxicity. Only Anabaena spp. can produce several kinds of toxins. The expensive ZN_0_, being a complex medium, is often replaced by media with diverse cheaper substitutes as ingredients instead of the original ones. The resulting toxicity level of the mixtures obtained needs further research.

Hazards accompanying PF utilized for H_2_ extraction from biomass are more numerous and more complex than for the PF process. Their interaction with the fermentation conditions is influenced by the type of inoculum and substrate. Thus, further research is required for this group.

Combining PF and DF processes enhances the efficiency and sustainability of H_2_ production. The implementation of such systems is capital-intensive and requires specialized infrastructure.

Biological methods allow for producing renewable H_2_ with minimal energy use, as they take place at normal pressure and temperature while also assisting in the recycling of organic waste. Biological H_2_ production methods are less influenced by the size of the plant compared to TC processes, yet they encounter different constraints. The primary disadvantages of biological methods are low H_2_ production rates and stringent operating conditions, including light intensity, temperature, H_2_O, pH, O_2_ exclusion, substrate concentration, and type. According to [39], incorporating metal-based nanoparticles, immobilizing microbes, genetically modifying microorganisms, and optimizing bioreactor design are among the strategies being explored to address these constraints and boost H_2_ production in biological processes. Even with extensive study in this area, scaling up biological technologies for H_2_ production largely hinges on creating novel solutions to enhance the efficiency and output of these processes.

The biological methods are currently at a low level of development, mainly on a laboratory scale. The TC ones can already provide a higher yield of produced H_2_. 

Supercritical water GA is more adaptable to various biomass types, including wet biomass (moisture > 35%), like carbohydrates and wood.

A key factor affecting the profitability of a TC plant is the kind of biomass utilized, as its composition affects the energy content and directly influences the H_2_ production generated. A greater energy content leads to a reduced breakeven point for the facility. The overall expenses of biomass also influence the costs associated with H_2_ production. Methods for lowering expenses and enhancing profitability and sustainability involve utilizing locally sourced and readily available residual biomass and waste, as well as recognizing synergies with various sectors (such as agro-industry, forestry, and waste management). TC biomass-to-H_2_ initiatives must also handle economic limitations directly connected to the plant’s processing capabilities. Each technology is linked to a maximum-size plant, beneath which the project is financially infeasible. Generally, economies of scale are relevant, making larger biomass TC processing plants more economical. These processes are context-dependent, so conducting case studies is necessary to obtain a more accurate view of the project’s techno-economic viability.

Considering only the economic aspects, the level of technological advancement, and H_2_ generation, conventional GA stands out as the best method for producing renewable H_2_ from biomass so far. HT GA is expected to be the optimal technology for generating H_2_ from biomass (especially high-moisture feedstocks) soon, as it provides the advantages of efficient and competitively priced H_2_ production. In general, TC methods provide efficient options for large-scale centralized H_2_ production, whereas biological processes are better suited for small-scale local generation.

Though only electrolysis and biomass GA have demonstrated effectiveness for the commercial production of H_2_, they remain inadequate to fulfill the worldwide demand, which is in agreement with the findings of [515]. 

The most information about hazards related to the implementation of TC processes of biomass was found for GA, then PY, and the least for HT processes. 

In case of hazards related to the gases released during H_2_ extraction from the biomass, catalysts, and promotors used, it is necessary to wear protective clothing, gloves, goggles, and sufficient respiratory protective equipment. It is necessary to avoid zones with elevated levels of toxic gases or material dust. It is necessary to prevent various materials (metals) from contacting an atmosphere containing active gases or vapors or reactive organic and inorganic substances. These depend on the types of considered materials, gases and other substances, and the levels of their tendency to undergo mutual chemical reactions.

The metallic materials used in catalysts in solid form are rather safe; however, their dust or fumes can be created during potential repairing, usually with various grinding operations. Then, proper protective equipment should be provided for workers.

In the case of the use of toxic materials, e.g., Co or Cr(VI), strict requirements must be maintained regarding their limited amounts in catalysts. Their use should be optimized, especially considering costs and catalytic efficacy. In addition, guidelines and good practices should be developed in each case to limit the impact of such materials on workers and other people in the vicinity of biomass processing plants, as well as on the environment. 

Most of the hazards found in the literature concerned pure metals used in catalysts. If they are used as compounds with other elements, for example, their oxides, or if hybrid catalysts using several metals are used, then in addition to the hazards characteristic of pure metals, other hazards may also arise, e.g., resulting from metal synergy. All such hazards must be identified early, hence the need for further studies focused on this.

To decrease the environmental impact of metals applied in various catalysts, it is recommended to widely use recycling for spent catalysts, if possible. However, for active materials, such as K, recycling is more difficult and requires the use of more advanced and expensive technologies.

Employees whose diet or medicine taken causes the risk of an excessive number of metals in their body, which can occur in catalysts, should not be exposed to air containing the vapors or dust of such metals. If they must stay in such conditions, they should use protective equipment with a high degree of protection. 

GSR is still being developed; however, although Ni-based catalysts are the most popular ones applied in reforming reactions, only a little information was found about hazards related to their application. Many other catalysts and promotors can be potentially utilized; however, their development is accompanied by various emerging risks and hazards, so further studies are needed on these.

TC methods, including GA and GSR, are efficient for H_2_ generation. Steam gasification is ideal for both wet and dry biomass without the presence of an oxidizing agent. DF is more effective for biological conversion, as it needs less energy. TC treatment is notably more developed than biological treatment regarding scaling possibilities when comparing existing processes. This is in agreement with observations made by [32].

Providing the high-quality H_2_ needed by an efficient power system requires the development of various efficient separation technologies. The most promising ones amount to four: membranes, adsorption processes, metal hydride (MH), and cryogenic processes. Further studies are needed on this topic (device design, hybrid material arrangement), also relating to other materials.

The determination of hazards related to H_2_ extraction from biomass methods is fragmentary. The determination of risk matrices (containing the probability of hazard occurrence) and the determination of prevention strategies require additional studies depending not only on the extraction method but also on the size of the plant, its equipment, and the level of training of employees.

## Figures and Tables

**Table 5:** Pros and cons of biological methods used for H_2_ extraction from biomass.

Methods	Pros	Cons
DbP	High efficacy, renewable, and sustainable	O_2_ sensitivity of H_2_ase, low catalyst availability
i-DbP	Separation of H_2_ and O_2_ generation, utilization of both H_2_ase and N_2_ase	Low efficacy, environmental sensitivity
PF	Uses a wide range of organic substrates, sustainable	Great energy expense, O_2_ sensitivity of N_2_ase
DF	Endless generation, wide substrate availability, cheap	Low substrate conversion efficacy, occurrence of H_2_ consumers, volatile fatty acid drainage
IS	Increased biomass conversion	Capital-intensive, needs specialized framework

**Table 7:** Comparison of GA techniques based on the used oxidizing agent. Based on [67].

Oxidizing Agent in GY Technique	O_2_	Air	Steam
**Products**	CO, H_2_, LHC (CH_4_, C_2_H_4_), CO_2_	N_2_, CO, H_2_, CO_2_, LHC (CH_4_, C_2_H_4_), H_2_O	H_2_, CO, CO_2_, LHC (CH_4_, C_2_H_4_)
**Tar [g/kg]**	2.2–46	3.7–61.9	60–95
**Average H_2_/(steam–O_2_ mixture) ratio**	40%	15%	40%
**H_2_/CO ratio**	1	0.75	1.6
**Heating Value [MJ/Nm^3^]**	12–28	4–7	10–18
**Pros**	Exothermic oxidation generates heat for GA. Higher syngas quality Higher heating value Higher Cold-Gas efficacy Less tar and char	Simple Availability Least expense Exothermic oxidation generates heat for GA. Less tar and char	Higher syngas quality Higher heating value Higher Cold-Gas efficacy Higher H_2_ content Less CO_2_
**Cons**	High capital and operating expenses for air separation unit Energy demand for air separation unit High CO_2_ generation	High N_2_ content Gas dilution with N_2_ Lowest syngas quality Lowest heating value Lowest Cold-Gas-Efficiency Inappropriate to some applications High CO_2_ production	External heat supply needed to maintain the temperature Relatively high tar and char Energy and expense of steam generation Excess steam is favorable but lowers the reactor operating temperature Expense of dual separate reactors

## Data Availability

No new data were created.
